# Broadening *INPP5E* phenotypic spectrum*:* detection of rare variants in syndromic and non-syndromic IRD

**DOI:** 10.1038/s41525-021-00214-8

**Published:** 2021-06-29

**Authors:** Riccardo Sangermano, Iris Deitch, Virginie G. Peter, Rola Ba-Abbad, Emily M. Place, Erin Zampaglione, Naomi E. Wagner, Anne B. Fulton, Luisa Coutinho-Santos, Boris Rosin, Vincent Dunet, Ala’a AlTalbishi, Eyal Banin, Ana Berta Sousa, Mariana Neves, Anna Larson, Mathieu Quinodoz, Michel Michaelides, Tamar Ben-Yosef, Eric A. Pierce, Carlo Rivolta, Andrew R. Webster, Gavin Arno, Dror Sharon, Rachel M. Huckfeldt, Kinga M. Bujakowska

**Affiliations:** 1 Ocular Genomics Institute, Massachusetts Eye and Ear Infirmary, Department of Ophthalmology, Harvard Medical School, Boston, MA USA; 2 Retina Service, Department of Ophthalmology, Massachusetts Eye and Ear, Harvard Medical School, Boston, MA USA; 3 Institute of Molecular and Clinical Ophthalmology Basel (IOB), Basel, Switzerland; 4 Department of Ophthalmology, University of Basel, Basel, Switzerland; 5 Experimental Pathology, Institute of Pathology, Lausanne University Hospital, Lausanne, Switzerland; 6 Genetics Service, Moorfields Eye Hospital, London, UK; 7 UCL Institute of Ophthalmology, University College London, London, UK; 8 Department of Ophthalmology, Boston Children’s Hospital and Harvard Medical School, Boston, MA USA; 9 Department of Ophthalmology, Instituto de Oftalmologia Dr. Gama Pinto, Lisbon, Portugal; 10 Department of Ophthalmology, Hadassah Medical Center, Faculty of Medicine, The Hebrew University of Jerusalem, Jerusalem, Israel; 11 Department of Diagnostic and Interventional Radiology, Lausanne University Hospital and University of Lausanne, Lausanne, Switzerland; 12 St. John Eye Hospital, Jerusalem, Israel; 13 Department of Medical Genetics, Hospital Santa Maria, Centro Hospitalar Universitário Lisboa Norte (CHULN), Lisbon Academic Medical Center (CAML), Lisbon, Portugal; 14 Department of Genetics and Genome Biology, University of Leicester, Leicester, UK; 15 Ruth and Bruce Rappaport Faculty of Medicine, Technion-Israel Institute of Technology, Haifa, Israel

**Keywords:** Hereditary eye disease, Disease genetics

## Abstract

Pathogenic variants in *INPP5E* cause Joubert syndrome (JBTS), a ciliopathy with retinal involvement. However, despite sporadic cases in large cohort sequencing studies, a clear association with non-syndromic inherited retinal degenerations (IRDs) has not been made. We validate this association by reporting 16 non-syndromic IRD patients from ten families with bi-allelic mutations in *INPP5E*. Additional two patients showed early onset IRD with limited JBTS features. Detailed phenotypic description for all probands is presented. We report 14 rare *INPP5E* variants, 12 of which have not been reported in previous studies. We present tertiary protein modeling and analyze all *INPP5E* variants for deleteriousness and phenotypic correlation. We observe that the combined impact of *INPP5E* variants in JBTS and non-syndromic IRD patients does not reveal a clear genotype–phenotype correlation, suggesting the involvement of genetic modifiers. Our study cements the wide phenotypic spectrum of *INPP5E* disease, adding proof that sequence defects in this gene can lead to early-onset non-syndromic IRD.

## Introduction

Inherited retinal degenerations (IRDs) are a group of genetically and clinically heterogeneous disorders characterized by progressive photoreceptor loss due to genetic defects in ~270 genes, inherited in all Mendelian modes^1^. IRDs manifesting as an isolated phenotype, or non-syndromic IRDs, can be further classified based on their onset and degeneration patterns. Syndromic IRDs manifest as a clinical feature of a syndrome, such as ciliopathies that involve multiple organs and tissues, including the central nervous system, skeletal and reproductive system, kidney, liver, pancreas, lung, and neuroretina^2^.

Pathogenic variants leading to ciliopathies occur in genes playing either a structural or a functional role in the primary cilium, a specialized organelle protruding from most post-mitotic cells. Cilia act as antennae that “sense” the physical and biochemical stimuli of the cellular environment to promptly initiate the signaling cascades in response to those changes^3^. Primary cilia play an important role during embryogenesis and organ development and, therefore, ciliary dysfunction often leads to congenital or early-onset disease^2^. The photoreceptor outer segment is regarded as a specialized primary cilium detecting light stimuli and thus multi-organ ciliopathies often involve retina^4^. A broad phenotypic spectrum of ciliopathies, ranging from isolated tissues (e.g., retina) to multiple organ involvement have been described for many genes^5^.

Joubert syndrome (JBTS, OMIM #PS213300) is an example of ciliopathy with retinal involvement. JBTS is a genetically heterogeneous autosomal or X-linked recessive disorder, with currently 36 associated genes (https://www.omim.org/phenotypicSeries/PS213300). The diagnostic hallmark of JBTS is the abnormal development of the mid-hindbrain known as the “molar tooth sign,” a radiological finding detectable on axial magnetic resonance imaging (MRI) of the brain^6^. JBTS manifests with hypotonia, ataxia, developmental delay, irregular breathing patterns, abnormal eye movements, oculomotor apraxia, and intellectual disability. Extra-neurological findings such as retinal degeneration, coloboma, skeletal abnormalities, cystic kidney disease, liver fibrosis, endocrinological disorders may also be present^7^.

Pathogenic variants in the *Inositol Polyphosphate-5-Phosphatase E* gene (*INPP5E*) on chromosome 9 are a known cause of JBTS (OMIM #213300). *INPP5E* is a widely expressed ciliary gene^8^, encoding a 72-kDa (644 amino acid) phosphatase that plays a critical role in controlling ciliary growth and stability via the phosphoinositide 3-kinase signaling pathway^9^. To date, a total of 34 pathogenic *INPP5E* variants have been reported, 28 of which in patients with JBTS or MORM (Mental retardation, truncal obesity, retinal dystrophy, and micropenis) syndrome (OMIM #610156)^9–23^. These JBTS cases include eleven patients with no signs of IRD^10,15,16,18,20,21,23^. In addition, large mutational screening studies identified five early-onset non-syndromic IRD cases, harboring six *INPP5E* variants^11,24–26^. Since these studies lacked detailed phenotypic descriptions of patients, a clear association between *INPP5E* and non-syndromic IRD has not been established. Here, we report 16 non-syndromic IRD patients and two cases with an IRD and some JBTS clinical features from 12 unrelated families with pathogenic variants in *INPP5E*. Our study thus substantiates the involvement of *INPP5E* variants in non-syndromic retinal disease.

## Results

### Rare *INPP5E* variants associated with non-syndromic early-onset IRD

**Fig. 1:**
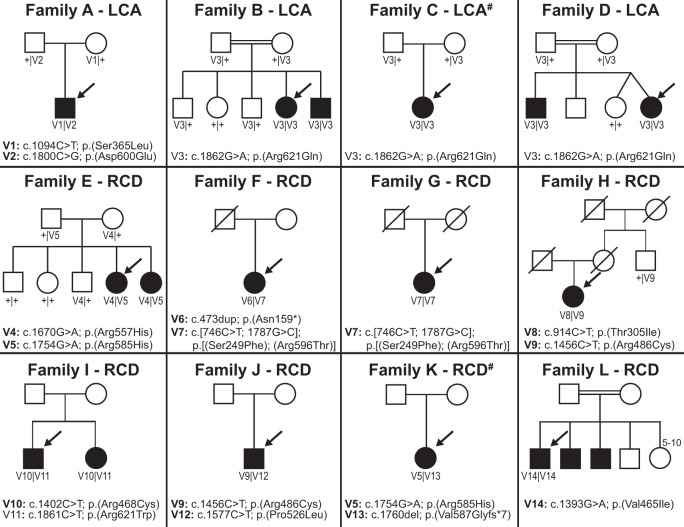
Pedigrees of the 12 *INPP5E* families described in this study. For each family, the specific IRD phenotype diagnosed is mentioned above each pedigree (LCA, Leber Congenital Amaurosis; RCD, Rod Cone Degeneration). Mildly syndromic families C and K are indicated with a hashtag (#). Affected male and female subjects are represented with black squares or circles, respectively. Probands are indicated by a black arrow. The five unaffected sisters in family L are indicated with the superscript 5–10. Novel variants are indicated in bold. When performed, segregation of the *INPP5E* variants in other family members is shown. First cousin marriage is indicated by a double line. All presented variants refer to the *INPP5E* transcript NM_019892.5.

**Fig. 2:**
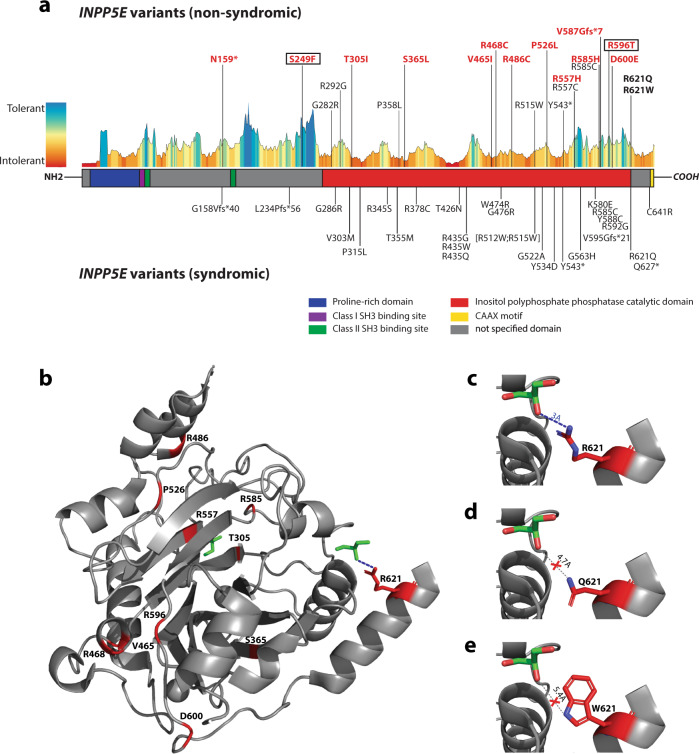
INPP5E structure and protein variants. **a** INPP5E secondary structure, tolerance landscape calculated using MetaDome, and distribution of known causal variants. Multiple sequence alignment-derived INPP5E motifs and catalytic domain were highlighted using different colors, while variants were divided into two groups, depending on whether they were found in syndromic or non-syndromic IRD patients. Variants found in our patients are in bold, while novel variants described in this study are additionally highlighted in red. Variants p.(Ser249Phe) and p.(Arg596Thr), found to be part of the same complex allele, are indicated by boxes. Variant Y543*, present in both syndromic and non-syndromic patients, results from two different nucleotide changes: c.1629C>G (JBTS) and c.1629C>A (IRD). **b** INPP5E tertiary structure. The tridimensional structure was predicted only for C-terminal 349 amino acids (residues 275–623) available on PDB (ID: 2XSW), as the N-terminal half was classified as a disordered region. Two glycerol molecules, acting as a proxy for the larger ligand of this protein (i.e., phosphatidylinositol polyphosphate), are shown in green. Amino acid residues for which missense variants in our patients were found are highlighted in red, except for Serine 249 located in the un-modeled region. **c–e** Predicted effect of missense variants p.(Arg621Gln) and p.(Arg621Trp) on ligand binding. In the wild-type protein model, Arginine 621 is located in close proximity (3 Å) to one glycerol molecule, which uniquely interacts by establishing one ion bond, indicated by a blue dashed line (**c**). Missense variants introducing Glutamine (**d**) or Tryptophan (**e**) are predicted to increase distance with the glycerol of 4.7 Å and 5.4 Å, respectively, thus disrupting the ion bond.

Sequencing analysis of ten recessive non-syndromic IRD families and two unrelated subjects with some JBTS clinical features uncovered ten likely pathogenic variants and five rare variants of unknown significance in *INPP5E* (see Fig. 1 and Table 1). Thirteen variants were novel, including two present as a complex allele p.[(Ser249Phe);(Arg596Thr)]. All *INPP5E* variants were rare (AF ≤ 0.0001 in gnomAD), had high CADD Phred scores (>20), and were predicted to be deleterious by several in silico prediction algorithms (see Fig. 2a, Table 1 and Supplementary Table 1)^27–31^. No other likely pathogenic variants in currently known IRD genes^1^ (see Supplementary Table 2) segregating with the phenotype were found.

**Table 1:** Genotypes of non-syndromic IRD probands carrying likely pathogenic alleles in *INPP5E*.

Family_ Proband	Proband_ Research_ID	Ethnicity	*INPP5E*_c. (NM_019892.5)	INPP5E_p. (NP_063945.2)	Segregation confirmed	gnomAD	CADD Phred score	ACMG
A.II-1	OGI3559_ 5164	Asian	c.1094C>T	p.(Ser365Leu)	PCR	absent	24.5	**VUS** (PM1, PM2, PP3)
			c.1800C>G	p.(Asp600Glu)		absent	24.6	**VUS** (PM1, PM2, PP3)
B.II-4	MOL0641-1	Arab	c.1862G>A	p.(Arg621Gln)	PCR	absent	27	**LP** (PM2, PM3, PM5, PP1)
			c.1862G>A	p.(Arg621Gln)		absent	27	**LP** (PM2, PM3, PM5, PP1)
C.II-1	LL135	White (Portuguese)	c.1862G>A	p.(Arg621Gln)	PCR	absent	27	**LP** (PM2, PM3, PM5, PP1)
			c.1862G>A	p.(Arg621Gln)		absent	27	**LP** (PM2, PM3, PM5, PP1)
D.II-4	LL105	White (Portuguese)	c.1862G>A	p.(Arg621Gln)	PCR	absent	27	**LP** (PM2, PM3, PM5, PP1)
			c.1862G>A	p.(Arg621Gln)		absent	27	**LP** (PM2, PM3, PM5, PP1)
E.II-4	OGI2307_ 3818	White	c.1670G>A	p.(Arg557His)	PCR	0.000012	25.2	**LP** (PM2, PM3, PM5,PS4_S)
			c.1754G>A	p.(Arg585His)		0.000064	28.4	**LP** (PM2, PM3, PP1, PP3)
F.II-1	OGI1819_ 3159	White	c.473dup	p.(Asn159*)	Cloning	0.000006	21.8	**LP** (PVS1, PM2)
			c.[746C>T; 1787G>C]	p.[(Ser249Phe); (Arg596Thr)]		0.000067; 0.000006	29.2; 25.9	**LP** (PM1, PM2, PP3, PP1)
G.II-1	OGI2386_ 3945	White	c.[746C>T; 1787G>C]	p.[(Ser249Phe); (Arg596Thr)]	n.t. (homo)	0.000067; 0.000006	29.2; 25.9	**LP** (PM1, PM2, PP3, PP1)
			c.[746C>T; 1787G>C]	p.[(Ser249Phe); (Arg596Thr)]		0.000067; 0.000006	29.2; 25.9	**LP** (PM1, PM2, PP3, PP1)
H.III-1	LL235	White (Portuguese)	c.914C>T	p.(Thr305Ile)	PCR	n.a.	27.1	**VUS** (PM1, PM2, PP3)
			c.1456C>T	p.(Arg486Cys)		0.000081	26.6	**LP** (PM1, PM2, PP3, PP1)
I.II-1	GC19652	White (British)	c.1402C>T	p.(Arg468Cys)	PCR	0.000012	32	**LP** (PM1, PM2, PP3, PP1)
			c.1861C>T	p.(Arg621Trp)		0.000016	29.8	**LP** (PM2, PM3, PP1, PM5)
J.II-1	GC16358	White (British)	c.1456C>T	p.(Arg486Cys)	PCR	0.000081	26.6	**LP** (PM1, PM2, PP3, PP1)
			c.1577C>T	p.(Pro526Leu)		0.000028	26.9	**VUS** (PM1, PM2, PP3)
K.II-1	GC22740	White (British)	c.1754G>A	p.(Arg585His)	PCR	0.000064	28.4	**LP** (PM2, PM3, PP1, PP3)
			c.1760delT	p.(Val587Glyfs*7)		0.000013	33	**LP** (PVS1, PM2)
L.II-1	TB315_R693	Arab	c.1393G>A	p.(Val465Ile)	n.t. (homo)	0.000018	23.4	**VUS** (PM1, PM2, PP3)
			c.1393G>A	p.(Val465Ile)		0.000018	23.4	**VUS** (PM1, PM2, PP3)

For complex alleles (in square brackets), gnomAD allele frequency was calculated independently for both variants. *n.t.*, not tested, CADD Phred score v1.6, *ACMG* American College of Medical Genetics, *PVS* pathogenic very strong, *PS4_S* pathogenic strong 4_supporting, *PM* pathogenic moderate, *PP* pathogenic supporting, *LP* likely pathogenic, *VUS* variant of unknown significance.

Two of the novel *INPP5E* variants were protein-truncating: (p.(Asn159*) and p.(Val587Glyfs*7)), while the remaining were missense. Most of the missense variants clustered in the highly conserved inositol 5-phosphatase catalytic domain (residues 273-621) and seven of them affected arginine residues (see Fig. 2a), which are known to have an important function in the catalysis of the phosphoryl group transfer^32^.

Affected subjects from five families carried homozygous variants: p.(Arg621Gln) in three unrelated families (families B-D), p.(Val465Ile) in family L, and a complex allele p.[(Ser249Phe); (Arg596Thr)] in family G (see Fig. 1 and Table 1). In three of these families, parents were first cousins (see Fig. 1).

The p.[(Ser249Phe);(Arg596Thr)] complex allele was identified in two unrelated patients, homozygous and compound heterozygous in family G and F, respectively. At present, it is not possible to determine which of the variants or both contribute to disease. The frequency of the p.(Ser249Phe) change is 11-times higher (AF = 0.000067) than of p.(Arg596Thr) (AF = 0.000006) in the gnomAD database (see Table 1), however, both are sufficiently rare to be potentially causal. Ser249 was predicted to be a phosphorylation site for the Protein Kinase C (NetPhos score = 0.84, intervals 0–1, see Supplementary Table 1), whereas Arg596 lies in the catalytic domain, though no specific effect of the p.(Arg596Thr) change was predicted (see Supplementary Table 1).

Apart from p.(Arg621Gln) and the p.[(Ser249Phe);(Arg596Thr)] complex allele, other recurrent missense variants were p.(Arg585His) in families E and K, p.(Arg486Cys) in families H and J (see Fig. 1 and Table 1).

All but one proband carried at least one likely pathologic *INPP5E* variant (see Fig. 1 and Table 1). The A.II-1 carried two variants of unknown significance (p.(Ser365Leu) and p.(Asp600Glu)). However, given the low mutational tolerance of these two residues and their localization at the catalytic domain, it is likely that they are causal (see Fig. 2a).

Alignment of INPP5E protein sequence in 100 species revealed that most of the missense changes were affecting highly conserved amino acids (identical in ≥98 of species), while variants p.(Ser249Phe), p.(Ser365Leu), p.(Pro526Leu) affected less conserved residues that were identical in 84, 70, and 46 species, respectively (see Supplementary Fig. 1).

### Protein modeling and prediction of missense variants at catalytic sites

Modeling of the tertiary structure of INPP5E (Protein Data Bank (PDB) ID: 2XSW; Tresagues et al., unpublished) predicted two sites of potential interaction with a ligand (glycerol molecule used as a proxy of inositol-3-phosphate) (see Fig. 2b). The first interaction site resides in the known catalytic domain where the ligand is predicted to form polar bonds with residues His424, Asn479, Asp477, and His584 (see Supplementary Fig. 2). Three of the likely pathogenic variants identified in this study are located either within the catalytic pocket: p.(Arg557His) and p.(Arg585His) or in its proximity: p.(Thr305Ile) (see Fig. 2b and Supplementary Fig. 2). They have the highest score for deleteriousness according to SuSPect^33^ (see Supplementary Table 1). The p.(Thr305Ile) change leads to the disruption of the hydrogen bond connecting Gln339 and Thr305 residues, which is thought to result in the alteration of the INPP5E structure (see Supplementary Fig. 3). None of these new or published likely pathogenic INPP5E changes directly affected the residues predicted to bind the IP3 ligand.

The second potential ligand interaction site resides outside of the known catalytic domain and exclusively involves the Arg621 residue. Two of the INPP5E variants detected in our patients (p.(Arg621Gln) and p.(Arg621Trp)) targeted the Arg621 residue. Modeling of the structural changes induced by these two variants showed that both lead to disruption of the polar bond connecting the second glycerol molecules to INPP5E protein (see Fig. 2c–e).

### Clinical phenotypes

**Fig. 3:**
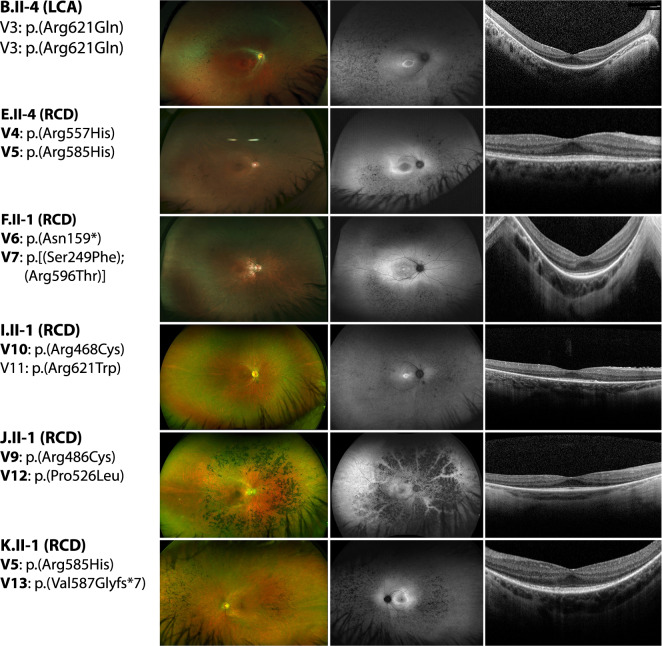
Clinical phenotypes of *INPP5E*-IRD patients. Images show fundus photos (left column), fundus autofluorescence (middle column), and OCTs (right column) for a representative subset of individuals. The specific IRD phenotype of each patient is given in brackets (LCA–Leber congenital amaurosis; RCD–Rod-cone degeneration). Novel *INPP5E* variants are highlighted in bold.

Eight females and four males with *INPP5E*-associated disease demonstrated features of IRDs that could be separated into two clinical categories. Probands of families A-D had a severe retinal degeneration manifested during early infancy (LCA) whereas the remaining eight had a milder juvenile-onset rod-cone degeneration (RCD) (see Supplementary Table 3). All individuals with LCA had nystagmus as a shared early feature. All four subjects had reduced visual acuity with severely constricted visual fields and undetectable or severely reduced electroretinograms (ERGs). Fundus examination and imaging showed macular and peripheral retinal atrophy. At least two LCA probands (B.II-4 and C.II-1) had structure-function dissociation based on the better foveal structure on optical coherence tomography (OCT) than would be expected from their visual acuities (see Fig. 3 and Supplementary Table 3).

Individuals with RCD first experienced nyctalopia and impaired dark adaptation beginning typically in childhood and their teens. None of the RCD subjects had nystagmus. Subjects had generally high visual acuities (see Supplementary Table 3). Goldmann perimetry showed mild constriction when available (*n* = 3). Full-field ERGs were performed in seven of eight subjects with RCD. Scotopic responses were undetectable in all but proband E.II-4 (at age 24) whereas 30 Hz flicker (photopic) responses were present and relatively preserved in five probands (E.II-4, F.II-1, I.II-1, K.II-1, and L.II-1) (see Supplementary Table 3). Fundus examination and wide-field fundus autofluorescence (FAF) imaging showed typical features of RCD in all individuals (see Fig. 3, left and middle panel). Macular OCT imaging showed central ellipsoid zone (EZ) preservation in most patients, and in proband E.II-4 the EZ was robust and identifiable through most of the scanned macula (see Fig. 3). Bilateral cystoid macular edema was present in proband H.III-1. Most individuals in both groups for whom information about refraction was available were myopic.

Two subjects showed extra-ocular features: subject K.II-1 presented with oculomotor apraxia and hypotonia at an early age which resolved, and the individual did not show any neurological or cognitive disability as an adult. Subject C.II-1 was found to have hypoplasia of the inferior cerebellar vermis on brain MRI at the age of 18 during an investigation of headaches (see Supplementary Fig. 4). She had a motor delay in infancy specifically delayed head control and sitting, as well as “lack of strength”, frequent falls, and learning difficulties in childhood. Despite this clinical history, other milestones including speech and walking were met at the appropriate age. This subject completed secondary education at age 18. On a recent neurological examination performed at age 22, mild ataxia and tandem gait disequilibrium was noted. Renal ultrasound performed at age 15 showed no renal anomalies. The remaining ten subjects in this cohort did not have other extra-ocular features.

### Meta-analysis of all pathogenic *INPP5E* variants and their phenotypic correlation

**Fig. 4:**
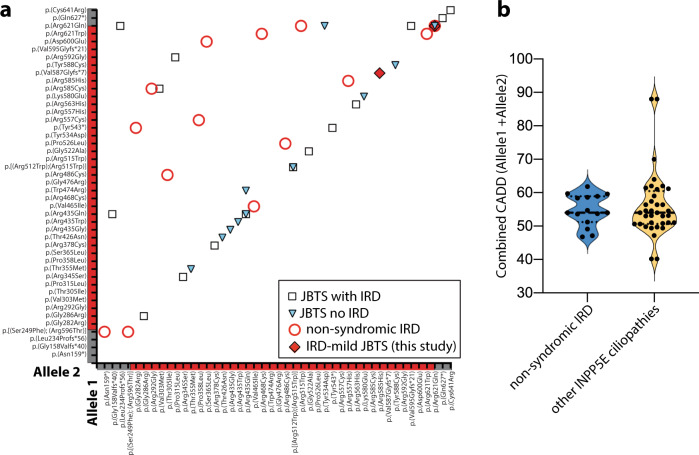
Meta-analysis of all pathogenic *INPP5E* variants and their phenotypic correlation. **a** Distribution of the *INPP5E* variants in all reported INPP5E patients. Four different phenotypes of increasing severity were marked by circles (non-syndromic IRD), diamonds (IRD-mild JBTS, reported in this study), squares (JBTS with IRD), and triangles (JTBS without IRD). **b** Violin plot of the cumulative CADD Phred scores for the *INPP5E* variants in syndromic (JBTS) and non-syndromic IRD cases.

Pathogenic variants in *INPP5E* can lead to a broad phenotypic spectrum ranging from severe ciliopathies to non-syndromic IRD^9–11,17,24–26^. We hypothesized that differences in disease severity are caused by more deleterious variants present in syndromic versus non-syndromic patients. Therefore, we gathered all published pathogenic variants in *INPP5E* (*n* = 47) in 34 syndromic (JBTS and MORM)^9–13,15–23,34^ and 17 non-syndromic IRD cases (our families and previously reported cases^11,24–26^) and analyzed their potential effect on protein function (see Figs. 2, 4, and Supplementary Table 4). First, we considered all alleles present in the syndromic cases (68 alleles from 34 patients) and alleles from non-syndromic IRD cases (34 alleles from 17 patients). We noticed that the difference in severity was not the result of a significantly higher frequency of loss-of-function (LoF) alleles in syndromic patients (9/68 alleles) compared to non-syndromic patients (3/34 alleles) (*χ*^2^ test, *p* = 0.5) and only three of the syndromic patients and none of the IRD patients were homozygous for an LoF variant (see Supplementary Table 4 and Fig. 4a). The remaining changes were missense variants, mostly within the inositol polyphosphate catalytic domain, with no apparent clustering based on the disease severity (see Fig. 2a). Only four variants were shared between syndromic and non-syndromic cases: p.(Arg515Trp)^10,11^, p.(Tyr543*)^16,26^, p.(Arg585Cys)^11,13,16^, and p.(Arg621Gln)^11,12,15–17^ (see Supplementary Table 4 and Fig. 4a). Using protein modeling and deleteriousness prediction algorithms we determined the potential impact of each variant on *INPP5E* function and for each patient added the impact of both variants. Overall, we did not find significant differences in conservation or deleteriousness scores of variants between the syndromic and non-syndromic cases (see Supplementary Tables 1 and 4 and Fig. 4b, Mann-Whitney test *p*-value > 0.05). One extreme example is the p.(Arg621Gln) variant, which has been observed as a homozygote in two non-syndromic and one mildly syndromic IRD cases in this study and recently reported in one subject with JBTS with no retinal degeneration^15^. These observations indicate that other genetic factors may play a role in the *INPP5E* disease manifestation.

## Discussion

We report non-syndromic IRD patients from ten families and two mildly syndromic JBTS cases with rare variants in *INPP5E*. Pathogenic variants in *INPP5E* are mainly known to cause systemic disorders, JBTS^10^ and MORM^9^, characterized by severe neurological manifestations and extra-neurological symptoms which may differ both quantitatively and qualitatively in each affected individual, even within the same family^10,12,16,21,34^. Therefore, the mostly non-syndromic patients described in this study are on a milder spectrum of the *INPP5E* disease. Although non-syndromic IRD due to *INPP5E* variants has been reported before in large mutational screening studies^11,24–26^, our report provides further evidence of the involvement of *INPP5E* variants in isolated retinal disease. We describe in detail retinal phenotypes of all the probands and report extraocular symptoms present in two patients. In addition, we present a thorough analysis of the functional impact that the identified variants may have on the INPP5E protein, using in-silico protein modeling tools. Our meta-analysis of all the published *INPP5E* variants and their potential phenotypic correlation illustrates the broad spectrum of phenotypes caused by the *INPP5E*-associated disease.

All probands described here were initially referred to as IRD patients at the time of their first visit. Ten of 12 presented with retinal degeneration with no other extra-ocular symptoms thus there was no indication for further clinical investigation other than ophthalmological. In probands, C.II-1 and K.II-1 mild ciliopathy features were identified during childhood. These symptoms, however, resolved during development and no cognitive or major neurological disability was present in adulthood. Hypoplasia of the inferior cerebellar vermis in C.II-1 was a secondary finding discovered by MRI performed to investigate the source of persistent headaches. Although this anatomical finding is less pronounced than the molar tooth sign usually detected in JBTS (see Supplementary Fig. 4), it is likely due to the homozygous p.(Arg621Gln) change in INPP5E and possibly additional genomic variability carried by this subject. A review of the exome sequencing (ES) data for this proband revealed a known pathogenic splicing variant (c.3290-2A>G) in *CPLANE1*, a gene associated with JBTS (OMIM # 614615) and Orofaciodigital syndrome VI (OFD, OMIM # 277170) that share some clinical features such as the molar tooth sign, vermis hypoplasia, and developmental delay^35^. The presence of this single allele in a recessive ciliopathy gene is however not sufficient to consider it as a modifier of the phenotype in patient C.II-1. A review of the genome sequencing data of proband K.II-1, with childhood oculomotor apraxia and hypotonia, did not return any rare coding variants (MAF < 0.001) nor CNVs in any of the Joubert or ataxia with oculomotor apraxia (OMIM# 208920, *APTX*)^36,37^ genes. Since brain MRI was not performed on the remaining cases we cannot rule out subclinical anatomical changes in these patients.

Of the 14 variants described in this study, 12 were novel and mainly resulting in missense changes of conserved amino acid residues in the phosphatase catalytic domain. Eight patients carried two likely pathogenic variants, two patients carried one likely pathogenic and one variant of unknown significance, and two cases carried two rare variants of unknown significance. Two variants in our study, p.(Arg621Gln) and p.(Arg621Trp), affected the same residue. Both variants were predicted to disrupt a unique polar bond between Arginine 621 and a potential ligand. Homozygous p.(Arg621Gln) and p.(Arg621Trp) changes were found in five patients, three non-syndromic LCA patients (this study and^24^), one mildly syndromic LCA case (C.II-1, this study), and one JBTS case without apparent retinal involvement^15^. The p.(Arg621Gln) change has also been associated with non-syndromic IRD cases and with JBTS without retinal involvement in a compound heterozygous scenario^11,16^. Unfortunately, at present, the paucity of genotyped *INPP5E* patients makes it impossible to explain the phenotypic discrepancies in patients carrying the p.(Arg621Gln) variant. Nevertheless, the frequency at which Arginine 621 is mutated suggests that this amino acid constitutes a critical residue for the INPP5E function and together with the putative ligand binding by Arg621, warrants expansion of the catalytic domain of INPP5E to this position. Of the 19 known pathogenic variants present in a homozygous state only three (p.[(Ser249Phe); (Arg596Thr)], p.(Val465Ile), p.(Arg621Trp)) resulted in a non-syndromic retinal degeneration, which may imply a hypomorphic or photoreceptor-specific impact of these variants on INPP5E function. These residues may also be important for photoreceptor-specific interactions with other ciliary proteins. Further functional studies will be needed to understand the impact of the identified *INPP5E* variants on the phosphatase activity or interactions with other proteins.

In order to understand the broad phenotypic spectrum of *INPP5E*-associated disease, we used several deleteriousness prediction algorithms and protein modeling to uncover the impact of each variant on the protein function. We have not found significant differences between the syndromic and non-syndromic cases or between the LCA and RCD cases, analyzing the combined impact of both *INPP5E* variants in each patient. The lack of a clear correlation of predicted variant impact on phenotype indicates that other genetic factors may play a role. Previous studies have shown that the INPP5E function in the cilium is dependent on other ciliary proteins, such as ARL3 and TULP3, and defects in those proteins lead to reduced or absent INPP5E localization to primary cilia^38,39^. Moreover, genetic modifiers in *cis* or *trans* to the primary disease variant(s) have been reported in many IRD studies where they influence disease penetrance, severity, and progression^40^. For example, the AHI1 variant p.(Arg830Trp) modifies the relative risk of retinal degeneration greater than seven-fold within a nephronophthisis cohort^41^. Similarly, resequencing of the *TTC21B* gene in a large group of clinically diverse ciliopathies showed that variants in this gene account as severity modifiers in ~5% of ciliopathy patients^42^. Although the number of genotyped samples with specific disease phenotypes is not large enough to support an unquestionable genotype-phenotype association, the rapid increase of high-throughput exome and genome sequencing in standard diagnostic protocols will help to validate some of these associations in the near future.

In conclusion, we provided further evidence for the involvement of *INPP5E* variants in non-syndromic IRD and demonstrated that these variants also account for previously underdiagnosed retinal degeneration patients.

## Methods

### Ethics statement

The study was approved by the institutional review board of all participating institutions (Partners HealthCare System for families E-G, the Boston Children’s Hospital Committee on Clinical Investigation for family A, Instituto de Oftalmologia Dr. Gama Pinto for families C, D, and H, the Institutional Review Boards and ethics committees of Moorfields Eye Hospital for families I-K, the institutional review board at Hadassah-Hebrew University Medical Center for family B, the Ethics Committee at Rambam Health Care Campus for family L) and adhered to the Declaration of Helsinki. Informed consent was obtained from all individuals on whom genetic testing and further molecular evaluations were performed.

### Clinical evaluation

Twelve probands with autosomal recessive retinal degeneration, who were part of larger IRD cohorts from five medical centers, were enrolled in this study. Four probands were ascertained from two different medical centers in Boston, USA (Massachusetts Eye and Ear and Boston Children’s Hospital), three in the United Kingdom (Moorfields Eye Hospital), three in Portugal (Instituto de Oftalmologia Dr. Gama Pinto), and two in Israel (Hadassah-Hebrew University Medical Center, Rambam Health Care Campus).

Clinical evaluation was performed by experienced ophthalmologists according to previously published protocols and included functional and structural assessments^43–46^.

For proband C.II-1, brain MRI was performed using a GE Signa HDxt 1.5T scanner (GE Medical Systems, Milwaukee, WI). The JBTS and control cases were scanned on a 3T scanner (Verio and Vida, Siemens Healthcare, Erlangen, Germany). Scanning protocols included unenhanced 3D T1 weighted Imaging and T2 spin-echo weighted imaging, which were sufficient to make the first diagnosis.

### Genetic analysis

Blood samples were obtained from probands, and when possible their parents, affected, and unaffected siblings. DNA was isolated from peripheral blood lymphocytes by standard procedures. Four probands (E.II-4, F.II-1, G.II-1, and A.II-1) were sequenced using the Genetic Eye Disease (GEDi) panel, described previously^47^. The GEDi version used in this study (v6) targeted exons of 278 known IRD genes (see Supplementary Table 2)^1^. The NGS data from the GEDi panel was analyzed using Genome Analysis Toolkit (GATK) version 3^48^ and annotated using the Variant Effect Predictor (VEP) tool^49^ with additional annotations taken from the Genome Aggregation Database (gnomAD)^27^, the Genomic Evolutionary Rate Profiling (GERP)^28^, SIFT^29^, PolyPhen2^30^, CADD Phred^31^, and retinal expression^50^. To detect possible copy number variations gCNV software was used as before^51^. The relatedness of the families sequenced with GEDi panel was excluded using Peddy^52^. Exome sequencing (ES) for five probands was performed at different facilities (B.II-4, Pronto Diagnostics Ltd; C.II-1, D.II-4 and H.III-1, Novogene (HK); L.II-1, Otogenetics Corporation), as previously described^45,53,54^. Finally, three patients (I.II-1, J.II-1, K.II-1) underwent genome sequencing (GS, Genomics England) according to previously published protocols^27,55^. In these patients, CNVs were interrogated by MANTA^56^ and CANVAS^57^ algorithms and direct inspection of the read data using IGV.

### Variant validation and phasing

All presented variants refer to the *INPP5E* transcript NM_019892.5. Variant segregation was performed by Sanger sequencing (primers in Supplementary Table 5) or analysis of NGS reads. For F.II-1, the three *INPP5E* variants detected were phased by cloning and Sanger sequencing (see Supplementary Fig. 5). Briefly, genomic DNA from the proband was amplified using Takara-LA (Takara Bio USA, Inc.) and primers spanning the region containing all variants. The amplified fragment was then cloned into the pCR2.1 plasmid, TA cloning kit (Invitrogen) and Sanger sequenced. Sanger sequencing was performed on ABI 3730xl (Applied Biosystems) using BigDye Terminator v3.1 kits (Life Technologies). Sequence analysis was done using SeqManPro (Lasergene 11, DNAStar Madison, WI, USA), in which variants were considered to be in *trans* when they were never present on the same clone.

### Multiple sequence alignment, protein modeling, and prediction of missense variants

Multiple sequence alignment of the human INPP5E protein and 99 orthologues was generated using Clustal Omega (https://www.ebi.ac.uk/Tools/msa/clustalo/)^58^ and sequences were retrieved from the UniProt Knowledgebase (UniProtKB, https://www.uniprot.org/help/uniprotkb)^59^. Tridimensional structure of the INPP5E protein, its putative catalytic sites, and mutated residues was generated with a protein modeling software (PyMOL Molecular Graphics System, Version 1.2r3pre, Schrödinger, LLC) using the crystal structure of human INPP5E as an input (Protein Data Bank (PDB) ID: 2XSW). The mutation tolerance at INPP5E protein residues was analyzed using MetaDome (https://stuart.radboudumc.nl/metadome/)^60^, while the impact of specific missense variants on INPP5E structure and function, was predicted by using four prediction algorithms: SIFT (https://sift.bii.a-star.edu.sg/)^29^, PolyPhen-2 (http://genetics.bwh.harvard.edu/pph2/)^30^, Missense3D (http://www.sbg.bio.ic.ac.uk/~missense3d/)^61^ and SuSPect (http://www.sbg.bio.ic.ac.uk/suspect)^33^. NetPhos 3.1 Server (http://www.cbs.dtu.dk/services/NetPhos/)^62^ was used to predict phosphorylation sites.

### Preprints

An earlier version of this manuscript has been published on MedRxiv.

### Reporting summary

Further information on research design is available in the Nature Research Reporting Summary linked to this article.

## Supplementary information

Supplementary Information

Reporting Summary

## Data Availability

Variants are available through ClinVar (Accession codes SCV001573305.1; SCV001573306.1; SCV001573392.1; SCV001573393.1; SCV001573394.1; SCV001573395.1; SCV001573396.1; SCV001573397.1; SCV001573398.1; SCV001573399.1; SCV001573400.1; SCV001573477.1; SCV001573478.1; SCV001573513.1; SCV001573569.1; SCV001573570.1; SCV001573637.1; SCV001573638.1; SCV001573767.1) and in Supplemental Materials.

## Code availability

Software used in this study: PyMOL Molecular Graphics System, Version 1.2r3pre; GraphPad Prism, version 9.0.2; SeqManPro (Lasergene v11).
