# Supplementary material for: Broadening INPP5E phenotypic spectrum: detection of rare variants in syndromic and non-syndromic IRD
Source: NPJ Genom Med. 2021 Jun 29;6:53. doi: 10.1038/s41525-021-00214-8 (PMC8242099; doi:10.1038/s41525-021-00214-8)
Supplement: Supplementary file 1 — Supplementary Information [file 41525_2021_214_MOESM1_ESM.pdf]

```
INP5E_Homo_sapiens                          -----------------------------------MPSKAEN-------LRPSEPA---- 14
INP5E_Pan_troglodytes                       -----------------------------------MPSKAEN-------LRPSEPA---- 14
A0A2K5K2A0_Colobus_angolensis_palliatus     ------------------------------------------------------------
A0A2K6PY13_Rhinopithecus_roxellana          -----------------------------------MPSKAES-------LRPSEPA---- 14
H2PTZ0_Pongo_abelii                         -----------------------------------MPSKADN-------LRPSEPA---- 14
A0A2K6U2B2_Saimiri_boliviensis_boliviensis  ------------------------------------------------------------
A0A2K6F2I5_Propithecus_coquereli            -----------------------------------MPSESVC-------LRP-------- 10
A0A2K6KCW3_Rhinopithecus_bieti              --------------------------------------QTET-------LSP-------- 7
A0A2K5MDJ3_Cercocebus_atys                  -----------------------------------MPSKAES-------LRPSEPA---- 14
A0A2K5XR75_Mandrillus_leucophaeus           ------------------------------------------------------------
A0A0D9RTF7_Chlorocebus_sabaeus              -----------------------------------MPSKAES-------LRPSEPA---- 14
A0A096NRS4_Papio_anubis                     -----------------------------------MPSKAES-------LRPSEPA---- 14
A0A2K5U0T2_Macaca_fascicularis              -----------------------------------MPSKAES-------LRPSEPA---- 14
F7DRP8_Macaca_mulatta                       -----------------------------------MPSKAES-------LRPSEPA---- 14
A0A2K5PHS4_Cebus_capucinus_imitator         -----------------------------------MPSKAAS-------LRPPEPA---- 14
A0A2K5CN95_Aotus_nancymaae                  -----------------------------------MPSKAAS-------LRPSEPA---- 14
A0A3Q0DNV4_Tarsius_syrichta                 ------------------------------------------------------------
H0XE73_Otolemur_garnettii                   -----------------------------------MPSESLC-------LRP-------- 10
I3N2L4_Ictidomys_tridecemlineatus           -----------------------------------MPTKATC-------MRHTE-A---- 13
F7BT66_Callithrix_jacchus                   -----------------------------------MPSKAAS-------LRPSEPA---- 14
A0A250Y3V1_Castor_canadensis                -----------------------------------MPSKSAC-------LRHTEA----- 13
A0A1S3G993Dipodomys_ordii                   ------------------------------------------------------------
H0VEZ3_Cavia_porcellus                      -----------------------------------MRSKAVC-------LRHTTAP---- 14
G5B6B1_Heterocephalus_glaber                -----------------------------------MPSKAVC-------LRHTAAP---- 14
A0A2Y9E841_Trichechus_manatus_latirostris   -----------------------------------MPSTSVS-------VPHAEGP---- 14
L5KA82_Pteropus_alecto                      ------------------------------------------------------------
A0A5N4EAQ5_Camelus_dromedarius              -----------------------------------MPSELAC-------VGHSEAA---- 14
A0A3Q2HCU7_Equus_caballus                   -----------------------------------MPSKPAC-------LGHSEAA---- 14
A0A1S2ZM74_Erinaceus_europaeus              -----------------------------------MPARLWS-------LGYSEPA---- 14
A0A3Q0DCZ7_Mesocricetus_auratus             -----------------------------------MPSKSAC-------LRHMEAP---- 14
G3I6Z4_Cricetulus_griseus                   -----------------------------------MPSKSAC-------LRHTEAP---- 14
INP5E_Rattus_norvegicus                     -----------------------------------MPSKSAC-------LRHTEAP---- 14
INP5E_Mus_musculus                          -----------------------------------MPSKSAC-------LRHTEAP---- 14
A0A2Y9NRK2_Delphinapterus_leucas            ------------------------------------------------------------
A0A2Y9SXS0_Physeter_macrocephalus           ------------------------------------------------------------
E1BAU6_Bos_taurus                           -----------------------------------MPSKLVS-------LGHSETA---- 14
A0A4W2BWU9_Bos_indicus_x_Bos_taurus         -----------------------------------MPSKLVS-------LGHSETA---- 14
L8IYW6_Bos_mutus                            -----------------------------------MPSKLVS-------LGHSETT---- 14
A0A452DQV4_Capra_hircus                     -----------------------------------MPSKLAS-------LGHSETA---- 14
A0A5J5MJZ6_Muntiacus_reevesi                -----------------------------------MPSKLAS-------LGHSEAA---- 14
A0A5N3WU40_Muntiacus_muntjak                -----------------------------------MPSKLAS-------LGHSEAA---- 14
A0A3Q7V225_Ursus_arctos_horribilis          -----------------------------------MPSKLVG-------LGHSEAS---- 14
A0A452Q8J6_Ursus_americanus                 -----------------------------------MPSKLAG-------LGHSEAS---- 14
A0A452VEA9_Ursus_maritimus                  -----------------------------------MPSKLVG-------LGHSEAS---- 14
A0A2U3ZR12_Odobenus_rosmarus_divergens      ------------------------------------------------------------
A0A3Q7N7C7_Callorhinus_ursinus              ------------------------------------------------------------
A0A2Y9HBQ8_Neomonachus_schauinslandi        ------------------------------------------------------------
A0A485NC41_Lynx_pardinus                    -----------------------------------MPSKLAC-------LGHSEAS---- 14
A0A3Q7QUW1_Vulpes_vulpes                    ------------------------------------------------------------
F1PDA7_Canis_lupus_familiaris               ------------------------------------------------------------
A0A2Y9JFT0_Enhydra_lutris_kenyoni           -----------------------------------MPSRLAG-------VGRSGAS---- 14
M3YJ90_Mustela_putorius_furo                -----------------------------------MPSRLEG-------VGCSGAS---- 14
I3JX61_Oreochromis_niloticus                ------------------------------------------------------------
A0A3Q4H5I7_Neolamprologus_brichardi         ------------------------------------------------------------
A0A3B4FRA1_Pundamilia_nyererei              ------------------------------------------------------------
A0A3P8QQM7_Astatotilapia_calliptera         ------------------------------------------------------------
W5M6X5_Lepisosteus_oculatus                 ------------------------------------------------------------
A0A3B3RRU9_Paramormyrops_kingsleyae         -----------------------------------MRMN--------------------- 4
A0A4W4HDW3_Electrophorus_electricus         ------------------------------------------------------------
A0A3Q3IZN0_Monopterus_albus                 ------------------------------------------------------------
A0A3P8VEE4_Cynoglossus_semilaevis           ------------------------------------------------------------
A0A3Q1H6D9_Anabas_testudineus               ------------------------------------------------------------
A0A4W6CEK3_Lates_calcarifer                 ------------------------------------------------------------
A0A3B4VK37_Seriola_dumerili                 ------------------------------------------------------------
A0A3Q3GNS7_Labrus_bergylta                  ------------------------------------------------------------
A0A3Q3RRU7_9TELE_Mastacembelus_armatus      ------------------------------------------------------------
A0A4U5UTG8_Collichthys_lucidus              ------------------------------------------------------------
A0A3B4ZI99_Stegastes_partitus               ------------------------------------------------------------
A0A3Q1H1R6_Acanthochromis_polyacanthus      ------------------------------------------------------------
A0A3Q1CXU7_Amphiprion_ocellaris             ------------------------------------------------------------
A0A3P8SMU2_Amphiprion_percula               ------------------------------------------------------------
A0A5J5DH05_Etheostoma_spectabile            ------------------------------------------------------------
A0A484DDW2_Perca_flavescens                 ------------------------------------------------------------
A0A4Z2H351_Liparis_tanakae                  ------------------------------------------------------------
W5UJJ6_Ictalurus_punctatus                  ------------------------------------------------------------
A0A5N5KDW8_Pangasianodon_hypophthalmus      ------------------------------------------------------------
A0A3P8ZCA3_Esox_lucius                      ------------------------------------------------------------
A0A4W5L9K3_Hucho_hucho                      ------------------------------------------------------------
A0A1S3NKU9_Salmo_salar                      ------------------------------------------------------------
W5L4Z3_Astyanax_mexicanus                   ------------------------------------------------------------
A0A3B4C5P1_Pygocentrus_nattereri            ------------------------------------------------------------
```

```
H3BEM7_Latimeria_chalumnae               ---------------------------------------MVC-------LWHMRIINDDV 14
A0A1L8F610_Xenopus_laevis                -------------------------------------------------MPL-------- 3
K7FPA9_Pelodiscus_sinensis               ----------------------------------------MN-------SSN-------- 5
A0A452GIT0_Gopherus_agassizii            ----------------------------------------MN-------PPN-------- 5
G3VXX0_Sarcophilus_harrisii              -----------------------------------MPSKPVM-------MDP-------- 10
A0A4X2KFU4_Vombatus_ursinus              -----------------------------------MPTKPVI-------MDP-------- 10
G1N6K2_Meleagris_gallopavo               ------------------------------------------------------------
F1P5J6_Gallus_gallus                     ----------------------------------------MS-------SLN-------- 5
U3IH52_Anas_platyrhynchos_platyrhynchos  ---------------------------------------------------L-------- 1
A0A151MFV8_Alligator_mississippiensis    ------------------------------------------------------------
A0A1U7RBH0_Alligator_sinensis            ------------------------------------------------------------
U3JGA7_Ficedula_albicollis               ----------------------------------------MS-------TPN-------- 5
A0A3L8SD89_Chloebia_gouldiae             ------------------------------------------------------------
H0Z776_Taeniopygia_guttata               ------------------------------------------------------------
A0A218V5X6_Lonchura_striata_domestica    ----------------------------------------MS-------TPN-------- 5
A0A0Q3MLL4_Amazona_aestiva               ----------------------------------------MS-------TPN-------- 5
A0A1V4J6C2_Patagioenas_fasciata_monilis  ----------------------------------------MS-------TPN-------- 5
A0A2I0M3Z8_Columba_livia                 ----------------------------------------MS-------TPN-------- 5
INP5E_Drosophila_melanogaster            MQHSSAGSEERITSSTSSPRSGHKTASSLFGLLSKRPSKVEPHPVTPESPRLQQRPVSAC 60


INP5E_Homo_sapiens                       ---------------------------PQP---------PEGRTLQGQLPGA----P--- 31
INP5E_Pan_troglodytes                    ---------------------------PQP---------PEGRTLQGQLPGA----P--- 31
A0A2K5K2A0_Colobus_angolensis_palliatus  ------------------------------------------------------------
A0A2K6PY13_Rhinopithecus_roxellana       ---------------------------PQL---------PEGRTLQGQLPGA----P--- 31
H2PTZ0_Pongo_abelii                      ---------------------------PQP---------PEGRTLQGQLPGA----P--- 31
A0A2K6U2B2_Saimiri_boliviensis_boliviensis ----------------------------MG---------TRPRWDLPGLPAA----A--- 16
A0A2K6F2I5_Propithecus_coquereli         ---------------------------PQQ---------PEGRMLKGQLPST----E--- 27
A0A2K6KCW3_Rhinopithecus_bieti           ---------------------------------------PETKTSK-------------- 14
A0A2K5MDJ3_Cercocebus_atys               ---------------------------PQP---------PEGRTLQGQLPGA----P--- 31
A0A2K5XR75_Mandrillus_leucophaeus        ------------------------------------------------------------
A0A0D9RTF7_Chlorocebus_sabaeus           ---------------------------PQP---------PEGRTLQGQLPGA----P--- 31
A0A096NRS4_Papio_anubis                  ---------------------------PQP---------PEGRTLQGQLPGA----P--- 31
A0A2K5U0T2_Macaca_fascicularis           ---------------------------PQP---------PEGRTLQGQLPGA----P--- 31
F7DRP8_Macaca_mulatta                    ---------------------------PQP---------PEGRTLQGQLPGA----P--- 31
A0A2K5PHS4_Cebus_capucinus_imitator      ---------------------------PQP---------PGGRTLQGPLPGA--DGA--- 33
A0A2K5CN95_Aotus_nancymaae               ---------------------------PQP---------PEGRTLQGPLPGA--DGA--- 33
A0A3Q0DNV4_Tarsius_syrichta              ------------------------------------------------------------
H0XE73_Otolemur_garnettii                ---------------------------PQQ---------SEGRMLKGQLPST--DKD--- 29
I3N2L4_Ictidomys_tridecemlineatus        ---------------------------PGQ---------LEARMLKGRLPGA--NKD--- 32
F7BT66_Callithrix_jacchus                ---------------------------PRP---------PEGRMLQGPLPGA--DGA--- 33
A0A250Y3V1_Castor_canadensis             -----------------------------------------PGQLQGQLPNT--DKD--- 27
A0A1S3G993Dipodomys_ordii                ------------------------------------------------------------
H0VEZ3_Cavia_porcellus                   ---------------------------G-Q---------LEAGMLKGQLPTP--AQE--- 32
G5B6B1_Heterocephalus_glaber             ---------------------------GQP---------EAGMMRKAQLPTP--AQD--- 33
A0A2Y9E841_Trichechus_manatus_latirostris ---------------------------PQ----------PGGTMLKEQLLPG--DKG--- 32
L5KA82_Pteropus_alecto                   ---------------------------------------------------M--DKD--- 4
A0A5N4EAQ5_Camelus_dromedarius           ---------------------------PQQPAA-QGHLQPEVRMLKGQLPNT--EED--- 41
A0A3Q2HCU7_Equus_caballus                ---------------------------PEQPTA-HGHLHPEGKMLKGQLPGT--DKD--- 41
A0A1S2ZM74_Erinaceus_europaeus           ---------------------------PQQ---------PEDTMFKGWLPNT--DKD--- 33
A0A3Q0DCZ7_Mesocricetus_auratus          ---------------------------GQ----------LEGRMLQGQLPNT--DKN--- 32
G3I6Z4_Cricetulus_griseus                ---------------------------GQ----------LEGRMLQGQLPNT--DKN--- 32
INP5E_Rattus_norvegicus                  ---------------------------GQ----------LEGRMLQGQLSNP--EKK--- 32
INP5E_Mus_musculus                       ---------------------------GQ----------LEGRMLQGQPPNT--EKK--- 32
A0A2Y9NRK2_Delphinapterus_leucas         -------------------------------------------MLKGRLPNT--DED--- 12
A0A2Y9SXS0_Physeter_macrocephalus        -------------------------------------------MLKGQLPNT--DED--- 12
E1BAU6_Bos_taurus                        ---------------------------SQQPTA-QGCLCPEGSMLKGLLPNS--DEE--- 41
A0A4W2BWU9_Bos_indicus_x_Bos_taurus      ---------------------------SQQPTA-QGCLCPEGSMLKGLLPNS--DEE--- 41
L8IYW6_Bos_mutus                         ---------------------------SQQPTA-QGCLCPEGSMLKGLLPNS--DEE--- 41
A0A452DQV4_Capra_hircus                  ---------------------------SQQPTV-QGCLRPEGSMLKGLLPNS--VEE--- 41
A0A5J5MJZ6_Muntiacus_reevesi             ---------------------------SQQPTP-QRCLHPEGSMLKGLLPSS--DEE--- 41
A0A5N3WU40_Muntiacus_muntjak             ---------------------------SQQPTP-QRCLHPEGSMLKGLLPSS--DEE--- 41
A0A3Q7V225_Ursus_arctos_horribilis       ---------------------------PPQPGA-QGHPQPEDRMLKGQLPNA--GKD--- 41
A0A452Q8J6_Ursus_americanus              ---------------------------PPQPGA-QGHPQPEDRMLKGQLPNA--GKD--- 41
A0A452VEA9_Ursus_maritimus               ---------------------------PPQPGA-QGHPQPEDRMLKGQLPNA--GKD--- 41
A0A2U3ZR12_Odobenus_rosmarus_divergens   -------------------------------------------MLKGQLPNT--GRD--- 12
A0A3Q7N7C7_Callorhinus_ursinus           -------------------------------------------MLKGQLPNT--GRG--- 12
A0A2Y9HBQ8_Neomonachus_schauinslandi     -------------------------------------------MLKGQLPNT--GRD--- 12
A0A485NC41_Lynx_pardinus                 ---------------------------PPQPTA-QGHLQPEGRMLKGQLPHA--GQD--- 41
A0A3Q7QUW1_Vulpes_vulpes                 -------------------------------------------MLKGQLPST--GKD--- 12
F1PDA7_Canis_lupus_familiaris            ----------------------------------MPSKLPEDRMLKGQLPST--GKD--- 21
A0A2Y9JFT0_Enhydra_lutris_kenyoni        ---------------------------PPQPAA-QGHLQPEDRMLKGQLPNM--GKD--- 41
M3YJ90_Mustela_putorius_furo             ---------------------------PPQPAA-QGHLQPEDRMLKGQLPNT--GKD--- 41
I3JX61_Oreochromis_niloticus             ------------------MTEDGEDGPLHHSCE---------------APGK--G----- 20
A0A3Q4H5I7_Neolamprologus_brichardi      ------------------MTEDGEDGPLHHSCE---------------APGK--G----- 20
A0A3B4FRA1_Pundamilia_nyererei           ------------------MTEDGEDGPLHHSCE---------------APGK--G----- 20
A0A3P8QQM7_Astatotilapia_calliptera      ------------------MTEDGEDGPLHHSCE---------------APGK--G----- 20
W5M6X5_Lepisosteus_oculatus              ------------------------------------------------------------
A0A3B3RRU9_Paramormyrops_kingsleyae      ---------------------------------------TLNGVWSGGTPEG--EGT--- 20
A0A4W4HDW3_Electrophorus_electricus      ------------------MNENGDAIVPFHPAE---------PPSRK----D--TR---- 23
A0A3Q3IZN0_Monopterus_albus              ------------------MTENGEDSSLHWSRE---------------APGK--G----- 20
```

```
A0A3P8VEE4_Cynoglossus_semilaevis          ------------------MTANGEDGSLHL------------------------------ 12
A0A3Q1H6D9_Anabas_testudineus              ------------------MTMNGEDINLHQSFE---------------APGK--G----- 20
A0A4W6CEK3_Lates_calcarifer                ------------------MTENGEDSSLHQNCQ---------------APDK--G----- 20
A0A3B4VK37_Seriola_dumerili                ------------------MTENGEDSSIHQYYE---------------APDK--G----- 20
A0A3Q3GNS7_Labrus_bergylta                 ----------------MAGHERMGDSLLRQYAE---------------APGQ--G----- 22
A0A3Q3RRU7_9TELE_Mastacembelus_armatus     ------------------MTDNGEDSRLHHVCE---------------APGK--E----- 20
A0A4U5UTG8_Collichthys_lucidus             ------------------MTENGEDSPLHQSCE---------------APDK--G----- 20
A0A3B4ZI99_Stegastes_partitus              ------------------MTENGEECTLHQSGE---------------APAK--G----- 20
A0A3Q1H1R6_Acanthochromis_polyacanthus     ------------------MTENGEDCPLHQSDE---------------APGK--G----- 20
A0A3Q1CXU7_Amphiprion_ocellaris            ------------------MTENGEDCPLHQSDE---------------APGK--G----- 20
A0A3P8SMU2_Amphiprion_percula              ------------------MTENGEDCPLHQSDE---------------APGK--G----- 20
A0A5J5DH05_Etheostoma_spectabile           ------------------MSENGEDSLLHQSCE---------------APAK--G----- 20
A0A484DDW2_Perca_flavescens                ------------------MTENGEDSLLHQSCE---------------APAK--G----- 20
A0A4Z2H351_Liparis_tanakae                 ------------------MTENGAHGPIHQSRQ---------------APSK--G----- 20
W5UJJ6_Ictalurus_punctatus                 ------------------MNENEDLIVPFQPQS---------EEALCRN--D--AA---- 25
A0A5N5KDW8_Pangasianodon_hypophthalmus     ------------------------------------------------------------
A0A3P8ZCA3_Esox_lucius                     ----------------MRMTENGEDSNPRQPVG---------GGLPGGAPDG--KDT--- 30
A0A4W5L9K3_Hucho_hucho                     ----------------MRMTENGEDSSPSQPD----------GVLPRGAPGG--KDP--- 29
A0A1S3NKU9_Salmo_salar                     ----------------MRMTENGEDSSPSQPD----------GVLPRGAPGG--KDP--- 29
W5L4Z3_Astyanax_mexicanus                  ------------------MNENGDTIVPFQPED---------EVRSKEPPSS--KT---- 27
A0A3B4C5P1_Pygocentrus_nattereri           ------------------MNENGETIVPFQPED---------EVNPEEPPCG--KD---- 27
H3BEM7_Latimeria_chalumnae                 P----GFETNTLNIELMRMDIQ--NGLSVQPIAGVSHSLTPTKLKHKQVENQ--EAE--- 63
A0A1L8F610_Xenopus_laevis                  -----------------------RME-RQNPGAVFASGED---------------NV--- 21
K7FPA9_Pelodiscus_sinensis                 -----------------------GFP--QHAVACV-TSSTEGRVLQDQQVKK--AGG--- 34
A0A452GIT0_Gopherus_agassizii              -----------------------GFS--PHAVACI-TSSTEGRVLQDQQVKK--AGG--- 34
G3VXX0_Sarcophilus_harrisii                -----------------------KHSVSQQSVACVSPQSDT------------------- 28
A0A4X2KFU4_Vombatus_ursinus                -----------------------KHSVSQQPVACVSPQSDT------------------- 28
G1N6K2_Meleagris_gallopavo                 ------------------------------------------------------------
F1P5J6_Gallus_gallus                       -----------------------GFS--QHSGTCI-TQRAEGGAVQDLQAKK--AGK--- 34
U3IH52_Anas_platyrhynchos_platyrhynchos    -----------------------GLEVSEPPGPLFASGPGPGGALLRPPGGA--PGA--- 33
A0A151MFV8_Alligator_mississippiensis      ------------------------------------------------------------
A0A1U7RBH0_Alligator_sinensis              --------------------------------------HADGRVL------R--EHQ--- 11
U3JGA7_Ficedula_albicollis                 -----------------------GFP--QHSRVCL-TRSMEGGAVQDLQAKK--AGK--- 34
A0A3L8SD89_Chloebia_gouldiae               ---------------------------------------MEGGAVQDLQAKK--AGK--- 16
H0Z776_Taeniopygia_guttata                 ------------------------------------------------------------
A0A218V5X6_Lonchura_striata_domestica      -----------------------GFP--QHSRLCL-TRSMEGGAVQDLQAKK--AGK--- 34
A0A0Q3MLL4_Amazona_aestiva                 -----------------------GFP--QHSGACV-TRSTEGGAVQDLHAKK--AGK--- 34
A0A1V4J6C2_Patagioenas_fasciata_monilis    -----------------------GFP--QHSGACA-TPSTEGGAVQDLHAKK--AGK--- 34
A0A2I0M3Z8_Columba_livia                   -----------------------GFP--QHPGACA-TPSTEGGAVQDLHAKK--AGK--- 34
INP5E_Drosophila_melanogaster              GQYDVSFAKCNADTSANHVNNNGSTLKS-QTLPLEKSHSGLISFHRRAKPSSSKGKNHSH 119


INP5E_Homo_sapiens                         -----PAQR-AG----SP-----PDAPGSESPALACSTPATPSGEDPP----ARAAPIAP 72
INP5E_Pan_troglodytes                      -----LAQR-AG----SP-----PDVPGSESPALACSTPATPSGEDPP----ARAAPIAP 72
A0A2K5K2A0_Colobus_angolensis_palliatus    -----------------------------------------------------------P 1
A0A2K6PY13_Rhinopithecus_roxellana         -----LAPR-AG----SP-----PDAPGSESPALACSTPATPSGEDPP----ARAAPVTP 72
H2PTZ0_Pongo_abelii                        -----LAQR-AG----SP-----PDAPGIESPALACSTPATPSGEDPP----ARAAPITP 72
A0A2K6U2B2_Saimiri_boliviensis_boliviensis -----LAPR-EE----SP-----HDAPSPESPALGGSAPAALSGEEPP----AQAAPIAP 57
A0A2K6F2I5_Propithecus_coquereli           -----KDLA-QS----SS-----PGSLPSTSPVLPFSIPAKLSSADPQ----TEAKPIAP 68
A0A2K6KCW3_Rhinopithecus_bieti             --------PK-TA----SK-----PR-GVKEINAFPCGSPGLPV---------------- 39
A0A2K5MDJ3_Cercocebus_atys                 -----LAPH-AG----SP-----PDAPGSESPALACSTPSTPSGEDPP----ARAAPVTP 72
A0A2K5XR75_Mandrillus_leucophaeus          ------------------------------------------------------------
A0A0D9RTF7_Chlorocebus_sabaeus             -----LAPR-AG----SP-----PDAPGSESPALACSTPATPSGEDPP----ARAAPVTP 72
A0A096NRS4_Papio_anubis                    -----LAPR-AG----SP-----PDAPGSESPALACSTPATPSGEDPP----ARAAPVTP 72
A0A2K5U0T2_Macaca_fascicularis             -----LAPR-AG----SP-----PDAPGSESPALACSTPATPSGEDPP----ARAAPVTP 72
F7DRP8_Macaca_mulatta                      -----LAPR-AG----SP-----PDAPGSESPALACSTPATPSGEDPP----ARAAPVTP 72
A0A2K5PHS4_Cebus_capucinus_imitator        -----LAPR-EG----SP-----PDAPGPESPALG--------GEGPP----AQAAPIAP 66
A0A2K5CN95_Aotus_nancymaae                 -----LAPR-EA----SP-----PDAPGPESPALGCSAPAALSGEEPP----APAAPLTP 74
A0A3Q0DNV4_Tarsius_syrichta                ------------------------------------------------------------
H0XE73_Otolemur_garnettii                  -----LAQS-SG----TGAPTTTCCQGPEKSPVLPFSIPAKLSSGDPQ----AKAKPITP 75
I3N2L4_Ictidomys_tridecemlineatus          -----LLPS-PG----ALP--TPHRQDPEKNPVPSFSDPAKISSEDPQ----ARAKPTTP 76
F7BT66_Callithrix_jacchus                  -----LAPR-EG----SP-----PDAPGPESPALGCSAPATLSGEETP----AQAVPIAP 74
A0A250Y3V1_Castor_canadensis               -----LVPS-TG----SLPV--THCQDPEKSPMPPLSIPAKTSNQDSR----KMAKPLAP 71
A0A1S3G993Dipodomys_ordii                  ------------------------------------------MTQDPQ----AEAKPVTP 14
H0VEZ3_Cavia_porcellus                     -----LCPG-TL----PG----ACYEAPEDGPTLPRSDRSKVDSETSPA---TAAEPLIP 75
G5B6B1_Heterocephalus_glaber               -----LPPT-PG----SPPA--ACCAAPEDGPTLPRDDRAKVDGETPP----ATAKPVTP 77
A0A2Y9E841_Trichechus_manatus_latirostris  -----LAQN-PP----APP---------------PTGSPGKTATKDTQ----AMVKALTP 63
L5KA82_Pteropus_alecto                     -----LALR-PG----SAPT--NTCPGLEKSPGVPLNIPAKISGEGPQ----AQAEPFTP 48
A0A5N4EAQ5_Camelus_dromedarius             -----PVPD-PW----SL-P--DSRQAPEKSPVLPPNIPAQVTDEDPH----AKARAFAP 84
A0A3Q2HCU7_Equus_caballus                  -----LTPC-PE----SLPT--DSCQGPEKSPVLPLNIPAKVSNEDPQ----AKANPFTP 85
A0A1S2ZM74_Erinaceus_europaeus             -----LTSN-PG----SPPT--SNCQGPEKSPALPSNISAKSH-DDPQ----TKAKLFAP 76
A0A3Q0DCZ7_Mesocricetus_auratus            -----LTPA-AG----SLPA--TEPQGSEANPTPPFSIPAKPSNQN-------------P 67
G3I6Z4_Cricetulus_griseus                  -----LTPT-PG----SLPA--TEPQGSETNPMPPFSIPAKPSNQN-------------P 67
INP5E_Rattus_norvegicus                    -----LIPT-SA----SLPA--ADSQSSQTNSMPPLSMPAKPSNQNLQ----AKANLITP 76
INP5E_Mus_musculus                         -----LIPT-PG----FLPA--SDSQGSETNPMPPFSIPAKTSNQNPQ----TKANLITP 76
A0A2Y9NRK2_Delphinapterus_leucas           -----LASR-PG----SAAA--DDRRGPEKSPVRPLDTPAQVGNEDPQ----ARGRPFSP 56
A0A2Y9SXS0_Physeter_macrocephalus          -----PA----------------------ARPVLPRDAPAPVGNEDPR----ATGRPFSP 41
E1BAU6_Bos_taurus                          -----PVPC-LG----SPAA--GGRQGPERSPELPLDTPEQVSNEDPR----AQARPFTP 85
A0A4W2BWU9_Bos_indicus_x_Bos_taurus        -----PVPC-LG----SPAA--GGHQGPERSPELPLDTPEQVSNEDPR----AQARPFTP 85
L8IYW6_Bos_mutus                           -----PVPC-LG----SPAA--GSRQGPERSPELPLDTPEQVSNEDPR----AQARPFTP 85
A0A452DQV4_Capra_hircus                    -----PVPC-LG----SPAA--GDRRGPERSSELPPDTPEQVSNKDPQ----AQARPFTP 85
```

```
A0A5J5MJZ6_Muntiacus_reevesi              -----PVPC-LG----SPAA--GDRQGPERSPELPLDTPEQVSNEDPQ----AQARPFTP 85
A0A5N3WU40_Muntiacus_muntjak              -----PVPC-LG----SPAA--GDRQGPKRSPELPLDTPEQVSNEDPQ----AQARPFTP 85
A0A3Q7V225_Ursus_arctos_horribilis        -----LPAH-PG----SPPT--NIAQGPEKSPVLPLNLPAQISNEDPQ----AKATPFTP 85
A0A452Q8J6_Ursus_americanus               -----LPAH-PG----SPPT--NIAQGPEKSPVLPLNLPAQISNEDPQ----AKATPFTP 85
A0A452VEA9_Ursus_maritimus                -----LPAH-PG----SPPT--NIAQGPEKSPVLPLNLPAQISNEDPQ----AKATPFTP 85
A0A2U3ZR12_Odobenus_rosmarus_divergens    -----LAAH-PG----SPPT--NNGQGPEKSPVLPLNLPAQISNEDPQ----AKATPFTP 56
A0A3Q7N7C7_Callorhinus_ursinus            -----LAAH-PG----SPPT--NNGQGPEKSPVLPLNLPAQISNEDPQ----AKATPFTP 56
A0A2Y9HBQ8_Neomonachus_schauinslandi      -----LEAH-PG----SPPT--NNGQGPEKSPVLPLNLSAQISNEDPQ----AKATPFTP 56
A0A485NC41_Lynx_pardinus                  -----LVPH-PG----SLPS--NGCQGPEKSPVPPLSLPAGIGNEDPQ----AKAKPFTP 85
A0A3Q7QUW1_Vulpes_vulpes                  -----VAPH-LG----PLPT--NHGQDPKRTPVLPLTFPAQISNEDPE----AKAKPFTP 56
F1PDA7_Canis_lupus_familiaris             -----VAPH-LG----PPPT--NHGQDPERTPVLPLTFPAQISNEDPE----AKAKPFTP 65
A0A2Y9JFT0_Enhydra_lutris_kenyoni         -----LAAH-PG----SPPA--NNGEGPEKSPVLPPKLPAQISNEDPQ----AKAASFIP 85
M3YJ90_Mustela_putorius_furo              -----LAAH-PG----SPPA--NSGEGPEKSPVLPPKLPAQISNEDPQ----AKATSFTP 85
I3JX61_Oreochromis_niloticus              -----DSIANDS----SKLKTSIVDVKSTNGHKDDHRL----------RNTGEGISPYQP 61
A0A3Q4H5I7_Neolamprologus_brichardi       -----DSIANDS----SKLNTSIVDVKSTNGHKDDLRL----------RNTGEGISPYQP 61
A0A3B4FRA1_Pundamilia_nyererei            -----DSIANDS----SKLNTSIVDVKSTNGHKDDLRL----------RNTREGISPYQP 61
A0A3P8QQM7_Astatotilapia_calliptera       -----DSIANDS----SKLNTSIVDVKSTNGHKDDLRL----------RNTGEGISPYQP 61
W5M6X5_Lepisosteus_oculatus               ----------------------------------------------M---TKERPHLP 9
A0A3B3RRU9_Paramormyrops_kingsleyae       -----GRLS-SG------QRSEKSCTDDEETFNDTLRFPADEVDRGGNANSSSPLHHHLP 68
A0A4W4HDW3_Electrophorus_electricus       ------SFHIEG----GKDGI-SNEIRSTKKSSEALLNPED--ER-N---KGSTISPYQP 66
A0A3Q3IZN0_Monopterus_albus               -----ESAVNAS----RSCKASLVDAKPCKEYNEALKLTA--------GNAEGEIIPYQP 63
A0A3P8VEE4_Cynoglossus_semilaevis         -----DSVVKDS----RPLKTSLVDGKPGKEHYVAQQKTL---------TNGTDATVYKP 54
A0A3Q1H6D9_Anabas_testudineus             -----DSIVNDS----RPCKTSLVDTNSCKEHNDALKPAV--------VNAQGEINPFQP 63
A0A4W6CEK3_Lates_calcarifer               -----DSVVTDS----RPRKTSLVDVKPCKEHSDALKLTA--------RKGEGEVSPYQP 63
A0A3B4VK37_Seriola_dumerili               -----DSVVNDS----RPLKTSLVDVKPCKEHNDALELAA--------RNREGEISPYQP 63
A0A3Q3GNS7_Labrus_bergylta                -----DPVVVDS----RALKTPLVTAKFSREPKDALKLSA--------TCAEGAVSPYQP 65
A0A3Q3RRU7_9TELE_Mastacembelus_armatus    -----DSVINDS----RPYEISLVDSKSCKEHNDALKLTA--------GNSEGEISPYQP 63
A0A4U5UTG8_Collichthys_lucidus            -----DVIDSIS----RSLKTSLEDAKTCKDHNDAPKLAA--------RNTDGDFGQYQP 63
A0A3B4ZI99_Stegastes_partitus             -----NSVANDS----KPIKTSLVDVKSPSEHNDELKIAAA------DKLAGGEFSPCQP 65
A0A3Q1H1R6_Acanthochromis_polyacanthus    -----NSVGNDS----KPLKTSLVDVTSPSEHNDELKLAAAAAAA-AARNAGGEISPYQP 70
A0A3Q1CXU7_Amphiprion_ocellaris           -----NSVGNDS----KPLKTSLVGVKSASEHNDELKLAA------AARNAGGEISPYQP 65
A0A3P8SMU2_Amphiprion_percula             -----NSVGNDS----KPLKTSLVGVKSASEHNDELKLAAA--AA-AARNAGGEISPYQP 68
A0A5J5DH05_Etheostoma_spectabile          -----DSLVSDS----RTLKTSVVDASSCKEHNDALKLTD--------RNAEGQISLYQP 63
A0A484DDW2_Perca_flavescens               -----DSLVSDS----RALETSLVDAKSCKEHNDALKLTD--------RNAEGAISLYQP 63
A0A4Z2H351_Liparis_tanakae                -----ASHVTDG----RPLTTSLVDPTSCRDHGDVHTLSA--------RNAEGEISPYQP 63
W5UJJ6_Ictalurus_punctatus                ------NFSIEV----ANDRSSKEQLSSKKSSNEVLQTPQD--EQ----REQDKPGPYIP 69
A0A5N5KDW8_Pangasianodon_hypophthalmus    -------MTEPI----KNS---------SLVRSRLMKCPRP--LK-MSKGNKTNAVPTCL 37
A0A3P8ZCA3_Esox_lucius                    -----GGFGGDK----A---------KTNKLLENTLKFPQ------DIGDQNSKTSPYQP 66
A0A4W5L9K3_Hucho_hucho                    -----GGVSSDK----RPVNPPTEDSKPTKLLNDTLKFPQ------DVQDQSSKTSLYQP 74
A0A1S3NKU9_Salmo_salar                    -----EGVSSDK----RPVNLPTEDNKPTKLL-DTLKFPQ------DVPDQSSKTSLYQP 73
W5L4Z3_Astyanax_mexicanus                 ------SFNIEG----GDGTNTE--PRATKKCNEDLHISQD--EQ-I---QETSLNPFQP 69
A0A3B4C5P1_Pygocentrus_nattereri          ------SFNVDG----VNKRTKKEQLKGTNNSSEVLQIPQD--ER-I---KETNISPYQP 71
H3BEM7_Latimeria_chalumnae                -----ERKKEE----IVESAEEEDYNKETTLNDTLKFPPEEVGHGIK----GAHKHFIP 110
A0A1L8F610_Xenopus_laevis                 -----SERKYSGIREWIEMQVD---QTLTHSMNESVRRGSTENPGA--VPSDVVPRPPCK 71
K7FPA9_Pelodiscus_sinensis                -----AMRKEAGDSRVLALKDH---PDIESFLNDTLRF----PPDE--IKPNMKIKSATP 80
A0A452GIT0_Gopherus_agassizii             -----AMRKEAGDSRVLALEDH---PDIESFLNDTLRL----PPDE--IKPNMKIKSVTP 80
G3VXX0_Sarcophilus_harrisii               ------MLKGEGLKNDLPERTEPCGNTSEFPFNRLLRL----TTEE--MKFNMKIKSITP 76
A0A4X2KFU4_Vombatus_ursinus               ------MLKEAGLKNELPERNEPCGNTSEFPFNRLLRL----TTEE--MKFNMKVKSITP 76
G1N6K2_Meleagris_gallopavo                ----------------------------------------------PEE--LKANVKIKSITP 15
F1P5J6_Gallus_gallus                      -----AAKKETSGNGVLTFESP---PRVDTLLNETLKL----LPDE--LKANVKVKSITP 80
U3IH52_Anas_platyrhynchos_platyrhynchos   -----AVA--AAGPGAAG-----------AE------L----A-----GPGPAAGGGRPA 60
A0A151MFV8_Alligator_mississippiensis     ------------------------------------------------------------
A0A1U7RBH0_Alligator_sinensis             -----AMQKEAGDSTASPTEDH---PDTGS-------------SGD--IKASLRATLFTP 48
U3JGA7_Ficedula_albicollis                -----AAKKEAGGDGT-----P---PKIGTPISETLKL----LPED--LKANMKIRSITP 75
A0A3L8SD89_Chloebia_gouldiae              -----AAKKEAGGDGA-----P---PKIGTPISETLKL----LPEE--LKANMKIKSITP 57
H0Z776_Taeniopygia_guttata                ----------------------------------------------PEE--LKANMKIKSITP 15
A0A218V5X6_Lonchura_striata_domestica     -----AAKKEAGGDGA-----P---PKIGTPISETLKL----LPEE--LKANMKIKSITP 75
A0A0Q3MLL4_Amazona_aestiva                -----AAKKDAGGNGALTFEDA---ASAGTALSETLKL----LPNE--LKANMKMKPLTP 80
A0A1V4J6C2_Patagioenas_fasciata_monilis   -----AAKKDPGDNELLTFEDP---PTVGTSLNETLKL----LPAE--LKANMKIKSFTP 80
A0A2I0M3Z8_Columba_livia                  -----AAKKEPGDNGLLTFEDP---PTVGTSLNETLKL----LPAE--LKANMKIKSFTP 80
INP5E_Drosophila_melanogaster             RHSTDAGSQSDGGADVVMRRKS----PKHRSPNH-NRFS-----HQFSLCCKAEKKPMTP 169


INP5E_Homo_sapiens                        RPPAR--PRLERALS---LDDKGWRR---RRFRGSQE-DLE--ARNGTSPSRGSVQS-EG 120
INP5E_Pan_troglodytes                     RPPAR--PRLERALS---LDDKGWRR---RRFRGSQE-DLE--ARNGTSPSRGSVQS-EG 120
A0A2K5K2A0_Colobus_angolensis_palliatus   RPPAR--PRLERALS---LDDKGWRR---RRFRGSQE-DLE--ARNGTSPSRRSVQS-EG 49
A0A2K6PY13_Rhinopithecus_roxellana        RPPAR--PRLERALS---LDDKGWRR---RRFRGSQEWDLE--ARNGTSPSRGTVQS-EG 121
H2PTZ0_Pongo_abelii                       RPPAR--PRLERALS---LDDKGWRR---RRFRGSQE-DLE--ARNGTSPSRGSEQS-EG 120
A0A2K6U2B2_Saimiri_boliviensis_boliviensis RPPAR--PRLERALS---LDDRGWRR---RRFRGSQE-NLE--ARNGTSPSRGSVQS-EG 105
A0A2K6F2I5_Propithecus_coquereli          RPPARPRPRLERALS---LDDRGWRR---RRFRGSHE-DLA--AQNGASPSRGSEQR-EA 118
A0A2K6KCW3_Rhinopithecus_bieti            ------------------LDHPAWWR---KEENQPLP-K--------QRPVPTAV-A-SS 67
A0A2K5MDJ3_Cercocebus_atys                RPPAR--PRLERALS---LDDKGWRR---RRFRGSQE-DLE--TRNGTSPSRGSVQS-EG 120
A0A2K5XR75_Mandrillus_leucophaeus         ------------------------------------------------------------
A0A0D9RTF7_Chlorocebus_sabaeus            RPPAR--PRLERALS---LDDKGWRR---RRFRGSQE-DLE--ARNGTSPSRGSVQS-EG 120
A0A096NRS4_Papio_anubis                   RPPAR--PRLERALS---LDDKGWRR---RRFRGSQE-DLE--ARNGTSPSRGSVQS-EG 120
A0A2K5U0T2_Macaca_fascicularis            RPPAR--PRLERALS---LDDKGWRR---RRFRGSQE-DLE--ARNGTSPSRGSVQS-EG 120
F7DRP8_Macaca_mulatta                     RPPAR--PRLERALS---LDDKGWRR---RRFRGSQE-DLE--ARNGTSPSRGSVQS-EG 120
A0A2K5PHS4_Cebus_capucinus_imitator       RPPAR--PRLERALS---LDDRGWRR---RRFRGSQE-DLE--ARNGTSPSRGSVRS-EG 114
A0A2K5CN95_Aotus_nancymaae                RPPAR--PRLERALS---LDDRGWRR---RRFRGSQE-DLE--ARNGTSPSRSSVQS-EG 122
A0A3Q0DNV4_Tarsius_syrichta               ------------------------------------------------------------
H0XE73_Otolemur_garnettii                 HPPAR--PRLERALS---LDDKGWRR---RHFQGSHD-DPE--AGNEACPSRASKQR-QA 123
```

```
I3N2L4_Ictidomys_tridecemlineatus         QPPTR--PRLERTLS---LDDKGWRR---RRFPRSQE-DLA--TQGGASPYKGSEQD-MA 124
F7BT66_Callithrix_jacchus                 RPPAR--PRLERALS---LDDRSWRR---RRFRGSQE-DLE--ARNGTSPSRGSVQS-DG 122
A0A250Y3V1_Castor_canadensis              QPPMR--PRLERALS---LDDKGWRR---RRFQSSQE-DLA--AQDGASPCRSSMQD-TA 119
A0A1S3G993Dipodomys_ordii                 QPPMK--PRLERALS---LDDKAWRR---RRFPNSQE-DLSAAERDGASPRAGSPHQAQG 65
H0VEZ3_Cavia_porcellus                    HPPAR--PRLERALS---LDDRGWRR---RRFRSSQE-DLA--SRDGAGPSEGSMQN--A 122
G5B6B1_Heterocephalus_glaber              HPAMR--PRMERALS---LDDRGWRR---RPFRSSQE-DLA--ARVEASPGGGSMQN-P- 124
A0A2Y9E841_Trichechus_manatus_latirostris MPPAR--PRLERTLS---LDEKGWRR---RRFRASQE-DLAGQDK--AGPC-------KD 105
L5KA82_Pteropus_alecto                    KPPAR--PKLERALS---LDEKGWRR---RRLQTSHE-DLE--ARSGASPSGGSLPD--Q 95
A0A5N4EAQ5_Camelus_dromedarius            KP-P-TRPRLERALS---LDEKGWRK---RRFRTSRE-DLA--GQSGASPSRGSLQD--E 131
A0A3Q2HCU7_Equus_caballus                 KPPTR--PRLERALS---LDEKGWRR---RRFRTSQE-DLA--SRNGASPSRGSSQD-EA 133
A0A1S2ZM74_Erinaceus_europaeus            KPPPM-RPRLERALS---LDEKSWRR---RRFRTSQE-DLD--SQNGTSPSRGSLKE--E 124
A0A3Q0DCZ7_Mesocricetus_auratus           QLPMR--PKLERTLS---LDDKGWRR---RRFPGSQE-DLT--VQNGASPCRVSTQD--S 114
G3I6Z4_Cricetulus_griseus                 QLPMR--PKLERTLS---LDDKGWRR---RRFQGSQE-DLT--VQNGASPCRGSMQD--S 114
INP5E_Rattus_norvegicus                   QPPIR--PKLERTLS---LDDKGWRR---RRFRGSQE-DLT--VQNGASPCRGSLQD--S 123
INP5E_Mus_musculus                        QPPIR--PKLERTLS---LDDKGWRR---RRFRGSQE-DLT--VQNGASPCRGSLQD--S 123
A0A2Y9NRK2_Delphinapterus_leucas          KPPP-PRPRLERALS---LDEKGWRK---RRFRTSRE-DLA--TRNGPSPSGGSLQD--E 104
A0A2Y9SXS0_Physeter_macrocephalus         KPPP-PRPRLERALS---LDEKGWRK---RRFRTSRE-DLA--ARNGASPSGGSLQD--E 89
E1BAU6_Bos_taurus                         KPPP-PRPRLERALS---LDEKGWRK---RRFRTSRE-DLT--VRNGASPSGGSLQD--E 133
A0A4W2BWU9_Bos_indicus_x_Bos_taurus       KPPP-PRPRLERALS---LDEKGWRK---RRFRTSRE-DLT--VRNGASPSGGSLQD--E 133
L8IYW6_Bos_mutus                          KPPP-PRPRLERALS---LDEKGWRK---RRFRTSRE-DLT--VRNGASPSGASLQD--E 133
A0A452DQV4_Capra_hircus                   KPPP-PRPRLERALS---LDEKGWRK---RRFRTSRE-DLT--VRNGASPSGGSLQD--E 133
A0A5J5MJZ6_Muntiacus_reevesi              KPPP-PRPRLERALS---LDEKGWRK---RRFRTSRE-DLT--VRNGASPSGGSLQD--E 133
A0A5N3WU40_Muntiacus_muntjak              KPPP-PRPRLERALS---LDEKGWRK---RRFRTSRE-DLT--VRNGASPSGGSLQD--E 133
A0A3Q7V225_Ursus_arctos_horribilis        KPPTR--PRLERALS---LDEKAWRR---RRFRTSHE-DLA--AHGGASPSRGSLQD--E 132
A0A452Q8J6_Ursus_americanus               KPPTR--PRLERALS---LDEKAWRR---RRFRTSHE-DLA--AHGGASPSRGSLQD--E 132
A0A452VEA9_Ursus_maritimus                KPPTR--PRLERALS---LDEKAWRR---RRFRTSHE-DLA--AHGGASPSRGSLQD--E 132
A0A2U3ZR12_Odobenus_rosmarus_divergens    KPPTR--PRLERALS---LDEKAWRR---RRFQTSHE-DLA--ARSGASPSRGSLQD--E 103
A0A3Q7N7C7_Callorhinus_ursinus            KPPTR--PRLERALS---LDEKAWRR---RRFQTSHE-DLA--ARSGASPSRGSLQD--E 103
A0A2Y9HBQ8_Neomonachus_schauinslandi      KPPTR--PRLERALS---LDEKAWRR---RRFRTSQE-DLA--ARGGASPSRGSLQD--E 103
A0A485NC41_Lynx_pardinus                  KPPTR--PRLERALS---LDEKAWRR---RRFRTSHE-DLA--TRSGTSPSRGSLQD--E 132
A0A3Q7QUW1_Vulpes_vulpes                  KPPLQ--PRLERALS---LDEKAWRR---RRFRTSPE-DLA--AGSGTGGSRGSLQD--E 103
F1PDA7_Canis_lupus_familiaris             KPPPQ--PRLERALS---LDEKAWRR---RRFRTSPE-DLA--AGSGAGGSRGSLQD--E 112
A0A2Y9JFT0_Enhydra_lutris_kenyoni         KPPPR--PRLERALS---LDEKAWRR---RRFPSSHE-DLA--AHVGASPCRGSSQD--E 132
M3YJ90_Mustela_putorius_furo              KPPPR--PRLERALS---LDEKAWRR---RRFPSSHE-DLA--ARGGASPSRGSSQD--E 132
I3JX61_Oreochromis_niloticus              RPPLLPRLSKSS--K-SISLEETVRT---RRLRNSQE-SLS--DPAETGSSTDSLKEDET 112
A0A3Q4H5I7_Neolamprologus_brichardi       RPPLLPRLSKSR--K-SISLEETVRT---RRLRNSQE-SLS--DPAETGSSTDSLKEDET 112
A0A3B4FRA1_Pundamilia_nyererei            RPPLLPRLSKSS--K-SISLEETVRT---RRLRNSQE-SLS--DPAETGSSTDSLKEDET 112
A0A3P8QQM7_Astatotilapia_calliptera       RPPLLPRLSKSS--K-SISLEETVRT---RRLRNSQE-SLS--DPAETGSSTDSLKEDET 112
W5M6X5_Lepisosteus_oculatus               RPPLLPKPSEQSSLGTRASNEEKLRR---RKLRTSQD-SLT--DPTGTGSSTDSLQDEKS 63
A0A3B3RRU9_Paramormyrops_kingsleyae       KPPSLMKTSKNSSLE---ANVKRRRI---KPLKTSQE-SLT--DRTGTGSSSDSLQDD-- 117
A0A4W4HDW3_Electrophorus_electricus       HPPFLPKPPSMGKGGKNPMSEEKIRG---RKLKSSQE-SLT--DPTETGSSTDSLKEDRA 120
A0A3Q3IZN0_Monopterus_albus               RPPSLPRLFKSS--K-SGSVDESVRT---RRLRNSQE-SLS--DSADIGSSTDSLREDQT 114
A0A3P8VEE4_Cynoglossus_semilaevis         LPPLLPRLSKSS--R-SSSLEETVRN---RRLRNSQE-SLS--DRADTGSSTDSLKEEMM 105
A0A3Q1H6D9_Anabas_testudineus             RPPSLPRLSKSN--K-SGSVEKTVRS---RRLRNSQE-SLS--DQAETGSSTDSLKEDQT 114
A0A4W6CEK3_Lates_calcarifer               RPPSLPRQAKSS--K-SGSVEETVRN---RRLRNSQE-SLS--DPAETGSSTDSLKEDQT 114
A0A3B4VK37_Seriola_dumerili               RPPSLPRQSKPS--K-SGSAAETVRN---RRLRNSQE-SLS--DPAETGSSTDSLKEDQT 114
A0A3Q3GNS7_Labrus_bergylta                RPPSQPRLS--G--K-SCSVEENLIT---RRLKNSQE-SLS--DPTETGSSTDSLREDQA 114
A0A3Q3RRU7_9TELE_Mastacembelus_armatus    RPPSLPRLSKSS--K-TGSVEETVRT---RRLRNSQE-SLS--DPAETGSSTDSLKEDQT 114
A0A4U5UTG8_Collichthys_lucidus            RPPSLPKLF--T--R-GGSVEETVRT---RRLRNSQE-SLS--DPAETGSSTDSLKENRT 112
A0A3B4ZI99_Stegastes_partitus             RPPLLPRLSRSS--R-SGSVEETVRT---RRLRNSQE-SLS--DPAETGSSTDSLREDQT 116
A0A3Q1H1R6_Acanthochromis_polyacanthus    RPPLLPRLSKSS--R-SGSIEETVRT---RRLKNSQE-SLS--DPTETGSSTDSLREDQT 121
A0A3Q1CXU7_Amphiprion_ocellaris           RPPLLPRLS--S--R-SGSIEETVRT---RRLKNSQE-SLS--DPTETGSSTDSLREDQT 114
A0A3P8SMU2_Amphiprion_percula             RPPLLPRLS--S--R-SGSIEETVRT---RRLKNSQE-SLS--DPTETGSSTDSLREDQT 117
A0A5J5DH05_Etheostoma_spectabile          RPPALPRLSKSR--K-GVSLEEAVRI---RRLRNSQE-SLS--DQVETGSSTDSLKEEQT 114
A0A484DDW2_Perca_flavescens               RPPALPRLSKSR--K-SVSVEEAVRT---RRLRNSQE-SLS--DQVGTGSSTDSLKEDQT 114
A0A4Z2H351_Liparis_tanakae                RPPSRPKLPKSN--RTSVSVEEAVRT---RRLRNSQE-SLS--DPVETGSSTDSLREDQP 115
W5UJJ6_Ictalurus_punctatus                RPPLLPKPLGLAKGSRNMSFEEKKKG---RRMRSSQE-SLT--DPAEMTSSTDSLREVSN 123
A0A5N5KDW8_Pangasianodon_hypophthalmus    IHPCFPSLLEWRKEVGTCRFEEKKKG---RRLRSSQE-SLT--DPAEMTSSTDSLREVST 91
A0A3P8ZCA3_Esox_lucius                    RPPPLPKPSALSVVGKGGSTEEKVRS---RRLKNSQE-SLT--DPAETSSSTDSLKEDSS 120
A0A4W5L9K3_Hucho_hucho                    RPPQLPKPPALSKGGTSGSV-EEVRS---RRLKHSQE-SLT--DPAETGSSTDSLKEDST 127
A0A1S3NKU9_Salmo_salar                    RPPPLPKPPALSKGGKSGSM-EEVRS---RRLKHSQE-SLT--DPAETGSSTDSLKEDST 126
W5L4Z3_Astyanax_mexicanus                 HPPSLPKPSGVGKGIRNFSFEEKTRR---RRLRSSQE-SLT--DPAETGSSTDSLREDST 123
A0A3B4C5P1_Pygocentrus_nattereri          HPPSLPKPPTVGKGSRNISFEEKLRR---RRLRSSQE-SLT--DPAETGSSTDSLREDAT 125
H3BEM7_Latimeria_chalumnae                KPPSKPKPPSQPQGEWASSPVEINRR---RKLKISQE-NLTDVS--EISSSAEFHPD--- 161
A0A1L8F610_Xenopus_laevis                 PKPRE--RRLERAVS---LDERHWRR----RFKASQD-SLR--DPDETGSSSGSLQEESA 119
K7FPA9_Pelodiscus_sinensis                KPPRK--PRLARASS---LDEKSWRKR--RLFRMSQE-NLI--DPNETSSSNGSLQETSL 130
A0A452GIT0_Gopherus_agassizii             KPPRK--PRLARASS---LDEKSWRRRR-RVFRTSQE-NLI--DPSETSSSNGSLQESSL 131
G3VXX0_Sarcophilus_harrisii               KPPRK--PKLERTFS---LDEKGWRR---RRFKTSHE-SLT--DPNETVSSSGSLKEVTP 125
A0A4X2KFU4_Vombatus_ursinus               KPPRK--PKLERTFS---LDEKGWRR---RRLKTSQE-SLT--DPNETGSSSGSLREVTT 125
G1N6K2_Meleagris_gallopavo                RPPRK--PRLERAAS---LDEKNWKRWR--RFRTSQE-SLT--DPNETSSSNGSLQEAAL 65
F1P5J6_Gallus_gallus                      RPPRK--PRLERAAS---LDEKNWKRWR--RFRTSQE-SLT--DPNETSSSNGSLQEAAL 130
U3IH52_Anas_platyrhynchos_platyrhynchos   PPPRK--PRLERAAS---LDEKNWRRWR--RFRTSQE-SLT--DPNETSSSNGSLQEASL 110
A0A151MFV8_Alligator_mississippiensis     ------------------------------------------------------------
A0A1U7RBH0_Alligator_sinensis             KPPRQ--PRLERAAS---LDEQSWRRRRRRLLKTSQE-SLT--DPNETSSSNGSLQEASP 100
U3JGA7_Ficedula_albicollis                RPPRK--PRLERAAS---LDEKSWRKWR--RFRTSQE-SLT--DPNETSSSNGSLQEASP 125
A0A3L8SD89_Chloebia_gouldiae              RPPRK--PRLERAAS---LDEKSWRKWR--RFRTSQE-SLT--DPNETSSSNGSLQEASP 107
H0Z776_Taeniopygia_guttata                RPPRK--PRLERAAS---LDEKSWRKWR--RFRTSQE-SLT--DPNETSSSNGSLQEASP 65
A0A218V5X6_Lonchura_striata_domestica     RPPRK--PRLERAAS---LDEKSWRKWR--RFRTSQE-SLT--DPNETSSSNGSLQEASP 125
A0A0Q3MLL4_Amazona_aestiva                RPPQK--PRLERAAS---LDEKSWRRWR--RFRTSQE-SLT--DPNEXSSSNGSLQEASL 130
A0A1V4J6C2_Patagioenas_fasciata_monilis   RPPRK--PRLERAAS---LDEKSWRRWR--RFRMSQE-SLT--DPNETSSSNGSLQEASL 130
A0A2I0M3Z8_Columba_livia                  RPPRK--PRLERAAS---LDEKSWRRWR--RFRTSQE-SLT--DPNETSSSNGSLQEASL 130
```

```
INP5E_Drosophila_melanogaster             PVTVR-------YNS--IPDSNSVL----RRDNLSLSWSMVDNNSGSLHSSEGSSHR--- 213


INP5E_Homo_sapiens                        PG-APAHSC--------SPPCLS--TSLQEIP------------------KSRGVLSSER 151
INP5E_Pan_troglodytes                     PG-APAHSC--------SPPCLS--TSLQEIP------------------KSRGVLSSER 151
A0A2K5K2A0_Colobus_angolensis_palliatus   PG-APAHSC--------SPPCLS--TSLQEIP------------------KSRGVLGSER 80
A0A2K6PY13_Rhinopithecus_roxellana        PG-APAHSC--------SPPCLS--TSLQEIP------------------KSRGVLGSER 152
H2PTZ0_Pongo_abelii                       PG-APAHSC--------SPPCLS--TSLQEIP------------------KSRGVS-SER 150
A0A2K6U2B2_Saimiri_boliviensis_boliviensis PG-APAHSV--------SPPCLS--ASLQEIP------------------KSRRAPGSER 136
A0A2K6F2I5_Propithecus_coquereli          PP-SPTPSL--------SPPCLS--TSLQEIP------------------KCRRGPGSEE 149
A0A2K6KCW3_Rhinopithecus_bieti            LG-APLWFS--------QDPTT-------KKP------------------KMSPSHA-SS 92
A0A2K5MDJ3_Cercocebus_atys                PG-APAHSC--------SPPCLS--TSLQEIP------------------KSRGVLGSER 151
A0A2K5XR75_Mandrillus_leucophaeus         ------------------------------------------------------------
A0A0D9RTF7_Chlorocebus_sabaeus            PG-APAHSC--------SPPCLS--TSLQEIP------------------KSRGVLGSER 151
A0A096NRS4_Papio_anubis                   PG-APAHSC--------SPPCLS--TSLQEIP------------------KSRGVLGSER 151
A0A2K5U0T2_Macaca_fascicularis            PG-APAHSC--------SPPCLS--TSLQEIP------------------KSRGVLGSER 151
F7DRP8_Macaca_mulatta                     PG-APAHSC--------SPPCLS--TSLQEIP------------------KSRGVLGSER 151
A0A2K5PHS4_Cebus_capucinus_imitator       PG-APAHSC--------SPPCLS--ASLQEIP------------------KSRRVPGSER 145
A0A2K5CN95_Aotus_nancymaae                PG-APAHSC--------SPPCLS--ASLQEIP------------------KSRRVPGSER 153
A0A3Q0DNV4_Tarsius_syrichta               ------------------------------------------------------------
H0XE73_Otolemur_garnettii                 PL-APAHSP--------SPPCLS--TSLQDIP------------------KCPRAPSSEG 154
I3N2L4_Ictidomys_tridecemlineatus         PP-GPGPQL--------PS-TLP--EHIFHLP------------------GV----AAQG 150
F7BT66_Callithrix_jacchus                 PG-APAHSC--------SPPCLS--ASLQEIP------------------KSRRVPGSER 153
A0A250Y3V1_Castor_canadensis              YG------H--------TLPCLS--TSLQEIP------------------KLPRAPSSEG 145
A0A1S3G993Dipodomys_ordii                 PAAAAAQDH--------PAPCLG--PSLQEVP------------------KARRAAGSEG 97
H0VEZ3_Cavia_porcellus                    PCKAPGRRS--------PLPCLS--TSLQEIP------------------KPRWASGSAG 154
G5B6B1_Heterocephalus_glaber              APRTPARSL--------PLPCLS--TSLQEIS------------------KPRRAVSSAG 156
A0A2Y9E841_Trichechus_manatus_latirostris PPEAPTCSS--------LPPGLS--TSLQEIP------------------ESHRAP---- 133
L5KA82_Pteropus_alecto                    TPGPPVHTC--------SPPGLS--TSLQEIP------------------MTRRVPGCAR 127
A0A5N4EAQ5_Camelus_dromedarius            APGTPTHSG--------SPPCLS--TSLQEIP------------------TTRRAPCSLG 163
A0A3Q2HCU7_Equus_caballus                 PG-PPAPSG--------PPPCLS--TSLQEIP------------------TSRRPP---- 160
A0A1S2ZM74_Erinaceus_europaeus            PSGPSTHCG--------PLPCLS--TSLQEIP------------------TSRRAPSNAG 156
A0A3Q0DCZ7_Mesocricetus_auratus           DTQSPACSR--------PLPCLS--ASLQEIP------------------KTRRATGSEG 146
G3I6Z4_Cricetulus_griseus                 VTQFPACSR--------PLPCLS--TSLQEIP------------------KTRRATGSEG 146
INP5E_Rattus_norvegicus                   VAQSPAYSR--------PLPCLS--TSLQEIP------------------KPRRATGSEG 155
INP5E_Mus_musculus                        VAQSPAYSR--------PLPCLS--TSLQEIP------------------KSRRATGSEG 155
A0A2Y9NRK2_Delphinapterus_leucas          APGTATRGG--------SPPCLS--TSLQEIP------------------TTRRALG--S 134
A0A2Y9SXS0_Physeter_macrocephalus         APGTAARSG--------SPPCLS--TSLQEIP------------------TTRRAPG--S 119
E1BAU6_Bos_taurus                         VPGTPARSG--------SPPCLS--ASLQEIP------------------TARRAPG--S 163
A0A4W2BWU9_Bos_indicus_x_Bos_taurus       VPGTPARSG--------SPPCLS--ASLQEIP------------------TARRAPG--S 163
L8IYW6_Bos_mutus                          APRTPARSG--------SPPCLS--ASLQEIP------------------TARRAPG--S 163
A0A452DQV4_Capra_hircus                   APGTPARNG--------SPPCLS--TSLQEIP------------------TARRAPG--S 163
A0A5J5MJZ6_Muntiacus_reevesi              TPRTPARSG--------SPPCLS--ASLQEIP------------------TARRAPG--S 163
A0A5N3WU40_Muntiacus_muntjak              TPRTPARSG--------SPPCLS--ASLQEIP------------------TARRAPG--S 163
A0A3Q7V225_Ursus_arctos_horribilis        VPRPPTHTD--------SPPCLS--TSLQEIP------------------TSRRAQGSTG 164
A0A452Q8J6_Ursus_americanus               VPRPPTHTD--------SPPCLS--TSLQEIP------------------TSRRAQGSTG 164
A0A452VEA9_Ursus_maritimus                VPRPPTHTD--------SPPCLS--TSLQEIP------------------TSRRAQGSTG 164
A0A2U3ZR12_Odobenus_rosmarus_divergens    VPRPPTHTD--------SPPCLS--TSLQEIP------------------TSRRAQGSAG 135
A0A3Q7N7C7_Callorhinus_ursinus            VPRAPTHTD--------SPPCLS--TSLQEIP------------------TSRRAQGSAG 135
A0A2Y9HBQ8_Neomonachus_schauinslandi      VPRPPTHTD--------SPPCLS--TSLQEIP------------------TTRRAQGSAG 135
A0A485NC41_Lynx_pardinus                  APQPPTHTG--------SPPCLS--TSLQEIP------------------TSRRAQGSTG 164
A0A3Q7QUW1_Vulpes_vulpes                  VPRPPGPPG--------SPPCLS--TSLQEIP------------------TSRRAQDSAG 135
F1PDA7_Canis_lupus_familiaris             VPRPPGPPG--------SPPCLS--TSLQEIP------------------TSRRAQDSAG 144
A0A2Y9JFT0_Enhydra_lutris_kenyoni         VLRPPAHTD--------SPHCLS--ASLQEIP------------------TSRRAQGSAG 164
M3YJ90_Mustela_putorius_furo              VPRHPAHTD--------SAPCLS--ASWQELP------------------TSRRAQGSAG 164
I3JX61_Oreochromis_niloticus              IVAPSGFVL--------QGT-----AAV---RNERDPSARPHS-------------AVPT 143
A0A3Q4H5I7_Neolamprologus_brichardi       VVAPSGFVL--------QGT-----AAV---RNERDPSGRPHS-------------AVPT 143
A0A3B4FRA1_Pundamilia_nyererei            VVAPSGFVI--------QGT-----AAV---RNERDPSARPHS-------------AVPT 143
A0A3P8QQM7_Astatotilapia_calliptera       VVAPSGFVI--------QGT-----AAV---RNERDPSARPHS-------------AVPT 143
W5M6X5_Lepisosteus_oculatus               G-----H-------------SLL--NSIFALQDRQASLGNDQNSSTELLLRKSRNISSSH 103
A0A3B3RRU9_Paramormyrops_kingsleyae       VLGPPAFDG--------SARSPS----------RQWSIDGSLTDT-PVSVCPARDRGGSL 158
A0A4W4HDW3_Electrophorus_electricus       PPTLGSVTS--------L-------------KNGQT-GGSPSD-------------WVRG 145
A0A3Q3IZN0_Monopterus_albus               VPAAGGYVL--------SDA-----PAI----RNQDSNVGALS-------------TDTS 144
A0A3P8VEE4_Cynoglossus_semilaevis         VSALGAVVS--------A-T-----AT----ISNQDSSVRPLS-------------AAST 134
A0A3Q1H6D9_Anabas_testudineus             VSLEGGFAL--------SGS-----TNL----RSQDSNVRPLS-------------AVAI 144
A0A4W6CEK3_Lates_calcarifer               VAAPGGFVV--------SSA-----ATI---RN-RDTSVRPHS-------------AVAT 144
A0A3B4VK37_Seriola_dumerili               VPPPGGFVV--------SSA-----API---RN-QDSSVRPHS-------------AVTT 144
A0A3Q3GNS7_Labrus_bergylta                ALVAGTLLL--------RGA-----ATI---RNSKDANVRPVS-------------AVTR 145
A0A3Q3RRU7_9TELE_Mastacembelus_armatus    VPAPGVAMV-----------------------RNHNSNVRPLS-------------AVTT 138
A0A4U5UTG8_Collichthys_lucidus            VQAPGGLVL--------SGA-----AAATSTRNEQEPDVRPLS------------SAGVT 147
A0A3B4ZI99_Stegastes_partitus             VPPPGGFVL--------SGA-----VAI---RNNQDSNVRPRS-------------AATT 147
A0A3Q1H1R6_Acanthochromis_polyacanthus    VPPPGRFVF--------SGD-----ATV---RNSQDSSIRPRS-------------AATT 152
A0A3Q1CXU7_Amphiprion_ocellaris           VPPPDRFVF--------SGD-----ATV---KNSQDSSIRPRS-------------AATT 145
A0A3P8SMU2_Amphiprion_percula             VPPPDRFVF--------SGD-----TTV---KNSQDSSIRPRS-------------AATT 148
A0A5J5DH05_Etheostoma_spectabile          APASGGFVL--------SGA-----ATI---RNDQDSNVRKLS-------------AVTT 145
A0A484DDW2_Perca_flavescens               APAPDGFVL--------SGA-----ATI---RNDQDSNMRQLS-------------AVTT 145
A0A4Z2H351_Liparis_tanakae                GPAPGGFVP--------I-------------RTDQDSITRPRS-------------AVAT 141
W5UJJ6_Ictalurus_punctatus                FSAPSSHMV--------L-------------KNGQDAGGSNFD-------------QVAW 149
A0A5N5KDW8_Pangasianodon_hypophthalmus    FPAPSSHMV--------L-------------KNGQNSGGSHFD-------------QVAW 117
A0A3P8ZCA3_Esox_lucius                    VT--SVFTL--------SSA-----SAL---RNGQSSILVPLS--------------APS 148
A0A4W5L9K3_Hucho_hucho                    VT--GVI-------------------TL---RNGQGSIVGPLL--------------TPT 149
```

```
A0A1S3NKU9_Salmo_salar                    VT--GVFTL--------SGA-----ATL---RNGQGSIVGPLL--------------TPT 154
W5L4Z3_Astyanax_mexicanus                 PV-LSSALG--------L-------------KNGQISGGIDFD-------------RATR 148
A0A3B4C5P1_Pygocentrus_nattereri          PA-LSSALE--------L-------------KNGQISGRGNFD-------------QAAG 150
H3BEM7_Latimeria_chalumnae                ------ELV--------DLPNLDHSTKLQNGMHSQ--------------------LSAQS 187
A0A1L8F610_Xenopus_laevis                 VLESAAQFTDEFKG---QNSCSQ-SLSLN------------------------------- 144
K7FPA9_Pelodiscus_sinensis                SPPTRSRVA----L---NG---N-EGSLHNLQDASE----------------PSYYRKNK 163
A0A452GIT0_Gopherus_agassizii             SPLTKSRVV----L---NG---D-QGSLHNLQDASE----------------PSPYGKNK 164
G3VXX0_Sarcophilus_harrisii               SPPTLTTLVHDGNHTSLNLNCLH-STS---LQDIPE----------------PKDIGNST 165
A0A4X2KFU4_Vombatus_ursinus               SPPTLTTLAHDGSHTPHNFNSLQ-STS---LQDIPE----------------PGDIGNST 165
G1N6K2_Meleagris_gallopavo                TSPIRGRGT----P------CCE-PNSLHSSPDASE----------------ESPVGKSK 98
F1P5J6_Gallus_gallus                      TSPIRGRAT----P---CSQCCE-PNSLHSSPDASE----------------ESPVGKSK 166
U3IH52_Anas_platyrhynchos_platyrhynchos   SPPVRGRAT----P---CSQCCE-PNSLHSSPDASE----------------ASPMGKSR 146
A0A151MFV8_Alligator_mississippiensis     ------------------------------------------------------------
A0A1U7RBH0_Alligator_sinensis             SPPVVSRGA----S---LG---T-PWHPGSLRDAVE----------------TSSFGKSK 133
U3JGA7_Ficedula_albicollis                SPPTRGRAS----P---CNPCCQ-QDSLHSSPDALE----------------ASPGGKSR 161
A0A3L8SD89_Chloebia_gouldiae              SPPTRGRAS----P---CNPCCQ-QDSLHSSPDALE----------------ASPGGKSR 143
H0Z776_Taeniopygia_guttata                SPPTRGRAS----P---CNPCCQ-QDSLHSSPDALE----------------ASPGGKSR 101
A0A218V5X6_Lonchura_striata_domestica     SPPTRGRAS----P---CNPCCQ-QDSLHSSPDALE----------------ASPGGKSR 161
A0A0Q3MLL4_Amazona_aestiva                SPLTRGRVS----P---CHRCCH-QNSLHSSPDASE----------------ASAVGKSR 166
A0A1V4J6C2_Patagioenas_fasciata_monilis   SPPVRGRAS----P------CCP-QNSLHSSQDALE----------------ASPVGKSR 163
A0A2I0M3Z8_Columba_livia                  SPPVRGRAS----P------CCP-QNSLHSSQDALE----------------ASAVGKSR 163
INP5E_Drosophila_melanogaster             -PRP-------------SSECAS-----------------------------------AP 224


INP5E_Homo_sapiens                        GSPS------SGGNPLSGV------ASSS----PNLPHRDAAVAGSSPRLPSLLPPRPPP 195
INP5E_Pan_troglodytes                     GSPS------SGGNPLSGV------ASSS----PNLPHRDAAVAGSSPRLPSLLPPRPPP 195
A0A2K5K2A0_Colobus_angolensis_palliatus   GSPS------SGGNPLSGV------ASSS----PNLLHRDAAVVGSSSRLPSLLPPRPPP 124
A0A2K6PY13_Rhinopithecus_roxellana        GSPS------SGGNPLSGV------ASSS----PNLLHRDAAVVGSSSRLPSLLPPRPPP 196
H2PTZ0_Pongo_abelii                       GSPS------SGGNPLSGS--------SS----PNLPHRDAAVAGSSPRLPSLLPPRPPP 192
A0A2K6U2B2_Saimiri_boliviensis_boliviensis GSPS------SGSDPLSGV------AGSA----PNLLHRDATVAGSSPRLPGLPPPRPLP 180
A0A2K6F2I5_Propithecus_coquereli          GSPS------SGGTCLSGV------LSGS----LDLLHRDELCTAHSPRLAGLRPPRPPP 193
A0A2K6KCW3_Rhinopithecus_bieti            REVG------RGCEPALGV------C---------------------------------- 106
A0A2K5MDJ3_Cercocebus_atys                RSPS------SGGNPLSGV------ASSS----PNLLHRDAAVAGSSPRLPSLLPPRPPP 195
A0A2K5XR75_Mandrillus_leucophaeus         ------------------------------------------------------------
A0A0D9RTF7_Chlorocebus_sabaeus            GSRS------SGVNPLSGV------ASSS----PNLLHRDAAVAGSSPRLPSLLPPRPPP 195
A0A096NRS4_Papio_anubis                   GSPS------SGG-PLSGV------ASSS----PNLLHRDAAVAGSSPRLPSLLPPRPPP 194
A0A2K5U0T2_Macaca_fascicularis            GSPS------SGGNPLSGV------ASSS----PNLLHRNAAVAGSSPRLPSLLPPRPPP 195
F7DRP8_Macaca_mulatta                     GSPS------SGGNPLSGV------ASSS----PNLLHRNAAVAGSSPRLPSLLPPRPPP 195
A0A2K5PHS4_Cebus_capucinus_imitator       GSPS------SGSDPLSGV------AASA----PNLLHRDATVAGSSPRLASLLPPRPLP 189
A0A2K5CN95_Aotus_nancymaae                GSPS------SGGDPLSGV------ASSA----PNLLHRDAAVAGSSPRLTSLLPPRPLP 197
A0A3Q0DNV4_Tarsius_syrichta               ------------------------------------------------------------
H0XE73_Otolemur_garnettii                 GSPS------PGGRCLSGV------LSCS----LDLLHREELCTEGSPRLASLLPPRPPP 198
I3N2L4_Ictidomys_tridecemlineatus         GCPS------------W------------------------------------------- 155
F7BT66_Callithrix_jacchus                 GGPS------SGGDPLFGV------ASSA----PNLLHGDATVAGSSPRLASLLPPRPLP 197
A0A250Y3V1_Castor_canadensis              GSPS------LWSNCLSGV------MSTS----LDLLHREATPARSSPRLPGLRHPPSAS 189
A0A1S3G993Dipodomys_ordii                 GSPP------GWSNRLSGV------ASTS----LDPLHREALPTGSSPLPPGLRPPRSAP 141
H0VEZ3_Cavia_porcellus                    GSPT------SWGDRLSGM------VSTS----LDLLHKEATLAGGSPRLASLHTPRPPS 198
G5B6B1_Heterocephalus_glaber              GSPT------SWGGRLSGR------VSTS----LDLLHREATLAGGSPKLATLRAPHPPP 200
A0A2Y9E841_Trichechus_manatus_latirostris GSPS------SWGNCLSGM------ISTS----LDLLHREAAATGSTPRLANVLPPCPLP 177
L5KA82_Pteropus_alecto                    GSPT------SWGNCISGM------VSSS----PDLLPRSGASAGGTPKLADVLPSRPLP 171
A0A5N4EAQ5_Camelus_dromedarius            GGPS------SWGNCLSGM------ISTS----LDLLHRDGASAGSTPRLASLLPPRPLP 207
A0A3Q2HCU7_Equus_caballus                 GSPS------LWGNCLSGM------ISTS----LDLLHRDGAAAGSTPRLAGVLPPRPVP 204
A0A1S2ZM74_Erinaceus_europaeus            GNPT------SWGSCLSGM------ISTS----LDVLHRDG--VGSTPRLASMLAPRSLP 198
A0A3Q0DCZ7_Mesocricetus_auratus           GSPS------LWSDCLPGM------ISTS----LDLLHRGTSPGGGSPKLASLHDAHTPP 190
G3I6Z4_Cricetulus_griseus                 GSPS------VWSDCLPGM------ISTS----LDLLHRETTPGGGSPKLASLHAAHTPP 190
INP5E_Rattus_norvegicus                   GSPS------LWSDCLSGM------ISTS----LDLLHREAASGGPHSRLASLRATHTPP 199
INP5E_Mus_musculus                        GSPS------LWSDCLSGM------ISTS----LDLLHRDAASGGPPSRLASLHASHTPP 199
A0A2Y9NRK2_Delphinapterus_leucas          GGPS------SLGNCLSGM------ISTS----LDLLHRDGASAGSTPRLASLLPPRPLP 178
A0A2Y9SXS0_Physeter_macrocephalus         GGPS------SRGNCLSGT------ISTS----LDLLHRDGASAGSAPRLASLLPPRPLP 163
E1BAU6_Bos_taurus                         GGPS------SWGNCLSGM------ISTS----LDLLHREGASAGSTPRLASLLSPRPLP 207
A0A4W2BWU9_Bos_indicus_x_Bos_taurus       GGPS------SWGNCLSGM------ISTS----LDLLHREGASAGSTPRLASLLSPRPLP 207
L8IYW6_Bos_mutus                          GGPS------SWGNCLSGM------ISTS----LDLLHREGASAGSTPRLASLLSPRPLP 207
A0A452DQV4_Capra_hircus                   GGPS------SWGNCLSGM------ISTS----LDLLHREGASAGNTPRLASLLSPRPLP 207
A0A5J5MJZ6_Muntiacus_reevesi              GGPS------SWGNCLSGM------ISTS----LDLLHREGASAGSAPRLASLLSLRPLP 207
A0A5N3WU40_Muntiacus_muntjak              GGPS------SWGNCLSGM------ISTS----LDLLHREGASAGSAPRLASLLSPRPLP 207
A0A3Q7V225_Ursus_arctos_horribilis        GSPS------SWGHCISGM------LSTS----LDLLHRDRASAGSTARLPAVD------ 202
A0A452Q8J6_Ursus_americanus               GSPS------SWGHCISGM------LSTS----LDLLHRDRASAGSTARLPAVD------ 202
A0A452VEA9_Ursus_maritimus                GSPS------SWGHCISGM------LSTS----LDLLHRDRASAGSTARLPAVD------ 202
A0A2U3ZR12_Odobenus_rosmarus_divergens    GSPN------SWGHCISGM------IGTS----LDLLHRDGALAGSPSRLPAVG------ 173
A0A3Q7N7C7_Callorhinus_ursinus            GSPN------SWGHCISGM------IGTS----LDLLHRDGALAGSPSRLPAVG------ 173
A0A2Y9HBQ8_Neomonachus_schauinslandi      GSPS------SWGHCLSGM------IGSS----LDLLHRDGALAGSPSRLPAVG------ 173
A0A485NC41_Lynx_pardinus                  GGPS------SWGHCISGM------ISTS----LDLLHRDGASAGSTTRLPAAD------ 202
A0A3Q7QUW1_Vulpes_vulpes                  GSPS------SWGHCISGM------ISTS----LDLLHRDGAVAETSVRLPPVD------ 173
F1PDA7_Canis_lupus_familiaris             GSPS------SWGHCISGM------ISTS----LDLLHRDGAVAGTSVRLPPVD------ 182
A0A2Y9JFT0_Enhydra_lutris_kenyoni         GSPS------SWGHCISGM------ISTS----LDVLHRDGASARSPARLPAVD------ 202
M3YJ90_Mustela_putorius_furo              GSPS------SWGHCISGM------ISTS----LDVLHRDGASAQSPARLPTVD------ 202
I3JX61_Oreochromis_niloticus              GSPA------FRDRGSSLS--DYERRPRS----QQSDLTVQQWRATKVRLSPVQPTGPLP 191
A0A3Q4H5I7_Neolamprologus_brichardi       GSPA------FRDRGSSLS--DYERRPCS----QQSDLTVQQWRAAKVRLSPVQPTGPLP 191
A0A3B4FRA1_Pundamilia_nyererei            GSPA------FRDRGSSLS--DYERRPRS----QQSDLTVQQWRAAKVRLSPVQPTGPLP 191
A0A3P8QQM7_Astatotilapia_calliptera       GSPA------FRDRGSSLS--DYERRPRS----QQSDLTVQQWRAAKVRLSPVQPTGPLP 191
W5M6X5_Lepisosteus_oculatus               SDPG------KRSSCHTLP------L--------KGDTQHQIIMSSKTKLSPLQHPGSLP 143
```

```
A0A3B3RRU9_Paramormyrops_kingsleyae        SEPDKK----SFKSCE---------------------PISNSAASSKIRLSPIHPSGPMP 193
A0A4W4HDW3_Electrophorus_electricus        ESPP------FRDRCSSLPG--EDE-CLH----GQGCLAGNKPKPSKIILSPLQPIGAFP 192
A0A3Q3IZN0_Monopterus_albus                VSPV------FRDRGNSLS--ECESRPHS----QQSDPTDHQVRPNKVLLSPVQPMGPLP 192
A0A3P8VEE4_Cynoglossus_semilaevis          RQSA------FRNRGSSLS--EVGRRPRS----QQSDLTEHGMRISKLCLSPVRASVPLP 182
A0A3Q1H6D9_Anabas_testudineus              GSPV------LRDRGSSFS--EDERRPHS----QQSDPTDNWMMSTKVHLSPVQPVGPLP 192
A0A4W6CEK3_Lates_calcarifer                GSPV------FRERGSSLS--EYERRPHS----QQSDPTDHRGRSTKQRLSPVQPMGPLP 192
A0A3B4VK37_Seriola_dumerili                GSPV------FRERGSSLS--EYDRRPHS----QQSDPTDHRMRSAKMRLSPVQPTGPLP 192
A0A3Q3GNS7_Labrus_bergylta                 GSPV------FRDRGRSLS--EYERRPTS----QQSDPVDHLVRPAKVRLTPVQPTGTLP 193
A0A3Q3RRU7_9TELE_Mastacembelus_armatus     RSPG------FRDRGNSLS--EFESRPCS----QQSDPIDQWVRSTKVRLSPVQPTGPLP 186
A0A4U5UTG8_Collichthys_lucidus             GSPV------FRDRGSSLS--EYEMRPPS----QQSDPTDHRARSTKVRLSPVQPTGPLP 195
A0A3B4ZI99_Stegastes_partitus              GSPV------FRDRGSSLS--EYERRPRS----QQSNPTDHRGRSAKLRLSPVQPTGTLP 195
A0A3Q1H1R6_Acanthochromis_polyacanthus     GSPV------FRDRGSSLS--EYERRPRS----QQSDPTEHRGRSAKLRLSPVQPTGPLP 200
A0A3Q1CXU7_Amphiprion_ocellaris            GSPV------FRDRGSSLS--EYERRPRS----QQSDPTDHRERSAKLRLSPVQPTGPLP 193
A0A3P8SMU2_Amphiprion_percula              GSPV------FRDRGSSLS--EYERRPRS----QQSDPTDHRERSTKLRLSPVQPTGPLP 196
A0A5J5DH05_Etheostoma_spectabile                                      GSPV------FRDRGRSLS--EYERRPTS----
QQSDPTDHRVRSSKVRLSPLQPTGTLP 193
A0A484DDW2_Perca_flavescens                GSPV------FRDRGRSLS--EYERRPTS----QQSDPTDHRARSSKVRLSPVQPTGTLP 193
A0A4Z2H351_Liparis_tanakae                 GSPV------SRDRGSSLSDSEYETRPHS----QQSDPTDHRARPARPRLSPVQPTGPLP 191
W5UJJ6_Ictalurus_punctatus                 QSPP------FWDRCNSFPE--DDR-CLS----GQGHFSGQHPKPSKIILSPLQPAGTFP 196
A0A5N5KDW8_Pangasianodon_hypophthalmus     QSPP------FRDRCSSFPE--EDR-CPP----GQGHFSGQHPKPSKIILSPLQPAGAFP 164
A0A3P8ZCA3_Esox_lucius                     RSPV------FRARGQSLS--EYERTSQD----HHRDDAEQRVWSSKVRLSPLQPSGTLP 196
A0A4W5L9K3_Hucho_hucho                     GSPV------FRARGQSLS--EYERRPHD----HHGDPAEQRGRPFKVRLSPLQPSGPLP 197
A0A1S3NKU9_Salmo_salar                     GSPV------FRARGQSLS--EYERRPHD----HHGDLAEQRGRPFKVRLSPLQPSGPLP 202
W5L4Z3_Astyanax_mexicanus                  ESPP------FRDRCSSLPE--ADK-HPS----RQSRLVELRAKPSKIILSPLQPTGSFP 195
A0A3B4C5P1_Pygocentrus_nattereri           ESPP------FRERCSSLPE--ADK-HPR----GQSRLAEQRAKQSRIILSPLHATGAFP 197
H3BEM7_Latimeria_chalumnae                 GRPNECVQQETGRNSSSNKS-DSENQDYS----RHSAAKGVKSPKAKSKLPTVTPTQPLP 242
A0A1L8F610_Xenopus_laevis                  -------------------------PMK----PGIVLGYPGNSSPSPRLSQIAPKHALP 174
K7FPA9_Pelodiscus_sinensis                 SSIS-------------ESEKQVSEVPTA----SVGFMQGKESSGSKSRLSKITPSRPLP 206
A0A452GIT0_Gopherus_agassizii              SSIS-------------DSKKQVSEVPGV----SVGLMQRKESLGSKPRLCKITPPRPLP 207
G3VXX0_Sarcophilus_harrisii                NPPS-------------SLEIIPSTVKSG----SLELVDGVTASGNKPRFSKILPPLALP 208
A0A4X2KFU4_Vombatus_ursinus                DPPS-------------SLEIIPPTVTSG----SLELLHGVTASWNKLRLSKVSPPLALP 208
G1N6K2_Meleagris_gallopavo                 GSTS-------------DLGKRASEISSA----FGGLLRGKAFAGSKPRLSQIMPARPLP 141
F1P5J6_Gallus_gallus                       GSTS-------------DLGKRASEISSA----FGGLLRGKAFAGSKPRLSQIMPARPLP 209
U3IH52_Anas_platyrhynchos_platyrhynchos    GSTS-------------DLGKRASEISSA----FGGLLRGKAFAGSKPRLSQIMPARPLP 189
A0A151MFV8_Alligator_mississippiensis      ------------------------------------------------------------
A0A1U7RBH0_Alligator_sinensis              GSGS-------------DSEKE-------------AFEQGKALSGGKPRLPQIAPPRALP 167
U3JGA7_Ficedula_albicollis                 GGAS-------------DLGKRASEISSA----FGGLLRGKGFAGGKPRLSQIMPARPLP 204
A0A3L8SD89_Chloebia_gouldiae               AGTS-------------DLGKRASEISSA----FGGLLRGKGFAGGKPRLSQIMPARPLP 186
H0Z776_Taeniopygia_guttata                 AGTS-------------DLGKRASEISSA----FGGLLRGKGFAGGKPRLSQIMPARPLP 144
A0A218V5X6_Lonchura_striata_domestica      AGTS-------------DLGKRASEISSA----FGGLLRGKGFAGGKPRLSQIMPARPLP 204
A0A0Q3MLL4_Amazona_aestiva                 GSPS-------------DLGKRASELSSA----FGGLLRGKAFAGSKPRLSQVMPARPLP 209
A0A1V4J6C2_Patagioenas_fasciata_monilis    GGTS-------------DLGKRASEISSA----FGGLLRGKAFAGGKPRLSQITPVRPLP 206
A0A2I0M3Z8_Columba_livia                   GGTS-------------DLGKRASEISSA----FGGLLRGKAFAGGKPRLSQITPVRPLP 206
INP5E_Drosophila_melanogaster              ESPV--ASKTFFAHCSPAAASASVARSESLQNRSPIRPMAVSACRSRLRLKLYPPGQELP 282


INP5E_Homo_sapiens                         ALS--LDIASDSLRTANK---VDS-D---LADYKLRAQPLLV---RAHSSLGPGRPRS-- 241
INP5E_Pan_troglodytes                      ALS--LDIASDSLRTANK---VDS-D---LADYKLRAQPLLV---RAHSSLGPGRPRS-- 241
A0A2K5K2A0_Colobus_angolensis_palliatus    ALS--LDIASDSLRTANK---VDS-D---LADYRLRAQPLLV---RAHSSLVPGRPRS-- 170
A0A2K6PY13_Rhinopithecus_roxellana         ALS--LDIASDSLRTANK---VDS-D---LADYRLRAQPLLV---RAHSSLVPGRPRS-- 242
H2PTZ0_Pongo_abelii                        ALS--LDIASDSLRTANK---VDS-D---LADYKLRAQPLLV---RAHSSLGPGRPRS-- 238
A0A2K6U2B2_Saimiri_boliviensis_boliviensis ALG--LDIASDSLRTANK---VDP-E---LADYRLRAQPRLV---RAHSSLGPGRPRS-- 226
A0A2K6F2I5_Propithecus_coquereli           AMD--LDITSSSLRTANK---VDS-D---HADYKLRMQTRLV---RAHSSLGPGRPRS-- 239
A0A2K6KCW3_Rhinopithecus_bieti             ---------------------------------AARSGPVFL---ESRQQR---RRVQ-- 125
A0A2K5MDJ3_Cercocebus_atys                 ALS--LDIASDSLRTANK---VDS-D---LADYKLRAQPLLV---RAHSSLGPGRPRS-- 241
A0A2K5XR75_Mandrillus_leucophaeus          ------------------------------------------------------------
A0A0D9RTF7_Chlorocebus_sabaeus             ALS--LDIASDSLRTANK---VDS-D---LAGYKLRAQPLLV---RAHSSLGPGQPRS-- 241
A0A096NRS4_Papio_anubis                    ALS--LDIASDSLRTANK---VDS-D---LADYKLRAQPLLV---RAHSSLGPGRPRS-- 240
A0A2K5U0T2_Macaca_fascicularis             ALS--LDIASDSLRTANK---VDS-D---LADYKLRAQPLLV---RAHSSLGPGRPRS-- 241
F7DRP8_Macaca_mulatta                      ALS--LDIASDSLRTANK---VDS-D---LADYKLRAQPLLV---RAHSSLGPGRPRS-- 241
A0A2K5PHS4_Cebus_capucinus_imitator        ALG--LDIASDSLRTANK---VDP-D---LADYKLRAQPRLV---RAHSSLGPGRPRS-- 235
A0A2K5CN95_Aotus_nancymaae                 ALG--LDIASCSLRTANK---VDP-D---LADYKLRAQPRLV---RAHSSLVPGRPRS-- 243
A0A3Q0DNV4_Tarsius_syrichta                ------------------------------------------------------------
H0XE73_Otolemur_garnettii                  AMD--LDVTSNSLRTANK---VDT-D---HADYKRRVQTRLV---RAHSSLGPGRPRS-- 244
I3N2L4_Ictidomys_tridecemlineatus          -------------------------------------ELP---EAGH-PPGPQPRS-- 170
F7BT66_Callithrix_jacchus                  ALG--LDIASDSLRTANK---VDP-D---LADYKLRAHPRLV---RAHSSLGPARPRS-- 243
A0A250Y3V1_Castor_canadensis               PID--LNVASDSLRTASK---VDS-E---YADHKLRIQTRLV---RANSSLGPGRSQS-- 235
A0A1S3G993Dipodomys_ordii                  ASD--HRAASDPASAAND---IDS-E---HADRKPRAQSKLV---RAHSSLGTCRPRS-- 187
H0VEZ3_Cavia_porcellus                     SE---PAMASDTLRTASR---LDS-E---QGDRKQGTPTRLV---RTHSSLGPGRPRS-- 243
G5B6B1_Heterocephalus_glaber               SSEPTMTMASEALRTASQ---VDS-E---QADREQGTPTRLF---RAHSSLGPSQPQS-- 248
A0A2Y9E841_Trichechus_manatus_latirostris  AMD--LSVSSDSLRMANK---VDP-D---REDFRRRVQ-KLV---RAHSSLGPSRPRS-- 222
L5KA82_Pteropus_alecto                     AID--WNTTSDSLRTTHR---VDA-D---HADYKLRLQTRLF---GAHSGLGPGRPPS-- 217
A0A5N4EAQ5_Camelus_dromedarius             AMD--QNVASNALRTANK---VDS-D---HADYKLRLQARLF---RAHSSLGPGRPPS-- 253
A0A3Q2HCU7_Equus_caballus                  AME--QAVVSHSLRTANK---VDE-D---HADYKLRLQTRLF---RAHSSLGPGRPPS-- 250
A0A1S2ZM74_Erinaceus_europaeus             AMD--LNVTSDSLRTANK---VDA-N---HADYKLGLQARLF---RAHSSLGPGLPPS-- 244
A0A3Q0DCZ7_Mesocricetus_auratus            AMD--LNIASNSLRTANK---VDP-E---HADYKLRMQTRLV---RAHSSLGSSRPRS-- 236
G3I6Z4_Cricetulus_griseus                  AMD--LNIASSSLRTANK---VDP-E---HADYKLRMQTRLV---RAHSSLGPSRPRS-- 236
INP5E_Rattus_norvegicus                    AMD--LNIASSSLRTANK---VDP-E---HTDYKLRMQNRLV---RAHSNLGPSRPRS-- 245
INP5E_Mus_musculus                         AMD--LSIASSSLRTANK---VDP-E---HTDYKLRMQTRLV---RAHSNLGPSRPRS-- 245
A0A2Y9NRK2_Delphinapterus_leucas           AMD--WNVASDALRTANK---VDL-D---HADRQLRWQARPF---RAHSSLRPGRPPS-- 224
A0A2Y9SXS0_Physeter_macrocephalus          AMD--WNVASDALRTANK---VGW-G---HADYQVRRQAGLF---RAHSSLGPGRPPS-- 209
```

```
E1BAU6_Bos_taurus                          AMA--RNMASEGLRTANK---VDP-D---HADYKLRLQARLF---RAHSSLGPGRPPS-- 253
A0A4W2BWU9_Bos_indicus_x_Bos_taurus        AMA--RNMASEGLRTANK---VDP-D---HADYKLRLQARLF---RAHSSLGPGRPPS-- 253
L8IYW6_Bos_mutus                           AMA--RNMASEGLRTANK---VDP-D---HADYKLRLQARLF---RAHSSLGPGRPPS-- 253
A0A452DQV4_Capra_hircus                    AMA--RNVASEGLRTANK---VDP-D---HTDYKLRLQARLF---RAHSSLGPGRPPS-- 253
A0A5J5MJZ6_Muntiacus_reevesi               AMG--RNVASEGLRTANK---VDP-D---HADCKLRLQARLF---RVHSSLGPGRPPS-- 253
A0A5N3WU40_Muntiacus_muntjak               AMG--RNVASEGLRTANK---VDP-D---HADYKLRLQARLF---RVHSSLGPGRPPS-- 253
A0A3Q7V225_Ursus_arctos_horribilis         -----QKVASHSLRSADR---VDP-G---QADCKSRLQSRLL---RAHSGLGPGRPPS-- 245
A0A452Q8J6_Ursus_americanus                -----QKVASHSLRSADR---VDP-G---QADCKSRLQSRLL---RAHSSLGPGRPPS-- 245
A0A452VEA9_Ursus_maritimus                 -----QKVASHSLRSADR---VDP-G---QADCKSRLQSRLL---RAHSGLGPGRPLS-- 245
A0A2U3ZR12_Odobenus_rosmarus_divergens     -----QKVASNSLRSADR---VDS-V---PTDCKSRLQSRLF---RAHSSLGPGRPPS-- 216
A0A3Q7N7C7_Callorhinus_ursinus             -----QKVASNALRSTDR---VDS-V---QTDCKSRLQSRLF---RAHSSLGPSRPPS-- 216
A0A2Y9HBQ8_Neomonachus_schauinslandi       -----QKVASNSLRSADR---VDL-V---QTDCKPRLQSRLF---RAHSSLGPGRPPS-- 216
A0A485NC41_Lynx_pardinus                   -----QKAASNPPWTAN-------------PTGKSRLQSRLF---RAHSSLGPGRPPS-- 239
A0A3Q7QUW1_Vulpes_vulpes                   -----PKVAPESLRPTHR---VDS-G---PADGKPHLQSRLF---RAHSSLGPGWPPS-- 216
F1PDA7_Canis_lupus_familiaris              -----PKVAPESLRPTHR---VDS-G---PVDGKPHLQSRLF---RAHSSLGPGRPPS-- 225
A0A2Y9JFT0_Enhydra_lutris_kenyoni          -----QKVASGSLRPADR---VDS-G---QADLKARLPSRLF---RAHSSLGPGRPPS-- 245
M3YJ90_Mustela_putorius_furo               -----QKVASGSRRPTDR---VDS-G---QADLKARLPSRLF---RAHSSLGPGRPPS-- 245
I3JX61_Oreochromis_niloticus               ALE--KSFASATLRAANR---IDR-D---CLDYAMPAREKLGER--LHRNLSDSRLLE-- 238
A0A3Q4H5I7_Neolamprologus_brichardi        ALE--KSFASATLRAANR---IDR-D---CLDYAMPAREKLGER--LHRNLSDSRLLE-- 238
A0A3B4FRA1_Pundamilia_nyererei             ALE--KSFASATLRAANR---IDR-D---CLDYAMPAREKLGER--LHRNLSDSRLLE-- 238
A0A3P8QQM7_Astatotilapia_calliptera        ALE--KSFASATLRAANR---IDR-D---CLDYAMPAREKLGER--LHRNLSDSRLLE-- 238
W5M6X5_Lepisosteus_oculatus                PVG--VNMASTSLRTANR---IDQ-D---CVDYAVIGKRGYE---RQHSSLSEYRLLD-- 189
A0A3B3RRU9_Paramormyrops_kingsleyae        AVE--LCTASTSLRTANR---IDR-D---CVDYGDTRRAGPE---RLHRNLSDSRLLE-- 239
A0A4W4HDW3_Electrophorus_electricus        PLE--NSITSLSLRSTNR---IDR-D---CLDYGRG---SPAER--LHRNFSDSLLLD-- 236
A0A3Q3IZN0_Monopterus_albus                ALE--KNFMSANLGAANR---IDR-D---CLDNAELA---SGDR--RHRILSDSQLLE-- 236
A0A3P8VEE4_Cynoglossus_semilaevis          ALE--KSFVSATIKAAKR---IDR-D---CLDYAVLAREKLGER--LHRNLSDSRLLE-- 229
A0A3Q1H6D9_Anabas_testudineus              ALE--KTFVSATLRPVNR---IDK-D---CLDYAVLHRENLGER--LHRNLSDSRLLE-- 239
A0A4W6CEK3_Lates_calcarifer                ALE--KSFVSATLRAANR---IDR-D---CLDYGVLAREKLRER--LHRNLSDSRLLE-- 239
A0A3B4VK37_Seriola_dumerili                TLE--KSFVSATLRAANR---IDR-D---CLDYAALAKENLGER--LHRNLSDSRLLE-- 239
A0A3Q3GNS7_Labrus_bergylta                 ALE--KSFA--TLRAANR---IDR-D---CLDYAVLAREKHGER--LHRNLSDSRLLE-- 238
A0A3Q3RRU7_9TELE_Mastacembelus_armatus     ALE--KSFASATLRAANR---IDR-D---CLDYSVLAKEKLGER--LHRNLSDSRLLE-- 233
A0A4U5UTG8_Collichthys_lucidus             ALE--KSFASATLRAANR---IDR-D---CLDYAVLAREKLGER--FHRNLSDSRLLE-- 242
A0A3B4ZI99_Stegastes_partitus              ALE--KSFASATLRAANR---IDR-D---CLDYAVLAREKLGER--LHRNLSDSRLLE-- 242
A0A3Q1H1R6_Acanthochromis_polyacanthus     ALE--RSFASATLRAANR---IDR-D---CLDYAVLAREKLGER--LHRNLSDSRLLE-- 247
A0A3Q1CXU7_Amphiprion_ocellaris            ALE--RSFASATLRAANR---IDR-D---CLDYAVLAREKLGER--LHRNLSDSRLLE-- 240
A0A3P8SMU2_Amphiprion_percula              ALE--RSFASATLRAANR---IDR-D---CLDYAVLAREKLGER--LHRNLSDSRLLE-- 243
A0A5J5DH05_Etheostoma_spectabile           ALG--KSFASDTLRAANR---IDG-D---CLDYAMMAREKLGER--LHRNLSDSRLLE-- 240
A0A484DDW2_Perca_flavescens                ALG--KSFASATLRAANR---IDG-D---CLDYAMMAREKLGER--LHRNLSDSRLLE-- 240
A0A4Z2H351_Liparis_tanakae                 ALE--KSFASATLRAANR---IDR-D---RVDYAVLAREKVGER--LPRNLSDSRLLD-- 238
W5UJJ6_Ictalurus_punctatus                 PLE--YSIAAMSLRTANQ---IDR-D---CLDYRNG---SLQEK--LQKNLSDSQLWD-- 240
A0A5N5KDW8_Pangasianodon_hypophthalmus     PLE--YSIASMSLRTANQ---IDR-D---CLDYRNG---SLQEK--LQKNLTDSQLWD-- 208
A0A3P8ZCA3_Esox_lucius                     ALE--QSLESVSLRATNR---IDR-D---CLDYGILPGKAGPERLHLHRNLSDSRLLE-- 245
A0A4W5L9K3_Hucho_hucho                     ALE--QTLASESLRTANR---IDR-D---CVDYGIVPGRAGPERLHLHRNLSDSRLLD-- 246
A0A1S3NKU9_Salmo_salar                     ALE--QTLASESLRTANR---IDR-D---CVDYGIVPGRAGPERLHLHRNLSDSRLLD-- 251
W5L4Z3_Astyanax_mexicanus                  PLD--YSVASMSLRTTNR---IDR-D---CLDYGKR---GLPER--LHRNFSDSRLLD-- 239
A0A3B4C5P1_Pygocentrus_nattereri           PVE--YNVASMSLRTTNR---IDR-D---CLDYGKR---GLTER--LHRNFSDSRLLE-- 241
H3BEM7_Latimeria_chalumnae                 PLD--MQATSHVLRTTNK---IDQ-D---CMDYAQTQASRFA---RQRRSLSDGKLQD-- 288
A0A1L8F610_Xenopus_laevis                  QIE--MNVTSRALRTANR---IDP-D---YMDYKEASSQLRCLR--QSSSLSDTRLHNNS 223
K7FPA9_Pelodiscus_sinensis                 PLE--LSMASHVLRTANR---IDS-D---YMDYQHYSQSRFERV---SSSLSDGRLHNS- 253
A0A452GIT0_Gopherus_agassizii              PLE--LSVASHVLRTANR---IDS-D---YMDYQHYSQSRFERL---SSSLSDTGLHNS- 254
G3VXX0_Sarcophilus_harrisii                PLE--LNVASETLRTANK---IDP-D---YVNYKHHAQSKLLRP---DSSLSDGRLQS-- 254
A0A4X2KFU4_Vombatus_ursinus                PLE--LNVASETLRTTNK---IDP-D---YVNYKHHTQSKLLRP---DSSLSDGRLQS-- 254
G1N6K2_Meleagris_gallopavo                 PME--LNVASHTLRTANK---IDP-D---LMDYRHYSQNKFGRV---SNSLSDTRLHGN- 188
F1P5J6_Gallus_gallus                       PME--LNVASHTLRTANK---IDP-D---LMDYRHYSQHKFGRV---SNSLSDTRLHGN- 256
U3IH52_Anas_platyrhynchos_platyrhynchos    PME--LNVASHTLRTANR---IDS-D---FMDYRHYSQHKFGRV---SSSLSDTRLHGN- 236
A0A151MFV8_Alligator_mississippiensis      -ME--IDVVSRALRTAHR---IDS-D---CTDYWRGSGSKLVRQ--SGGSLSDTRLHGS- 47
A0A1U7RBH0_Alligator_sinensis              PME--IDVVSRALRTAHR---IDS-D---CTDYRRGSGSKLARQ--SGGSLSDTRLHGS- 215
U3JGA7_Ficedula_albicollis                 PME--LNVASHSLRTANR---IDS-D---CLDYRHYSQHKFGRV---SSSLSDSRLQGN- 251
A0A3L8SD89_Chloebia_gouldiae               PME--LNVASHSLRTANR---IDS-D---CLDYRHYSQHKFGRV---SSSLSDSRLQGN- 233
H0Z776_Taeniopygia_guttata                 PME--LNVASHSLRTANR---IDS-D---CLDYRHYSQHKFGRV---SSSLSDSRLQGN- 191
A0A218V5X6_Lonchura_striata_domestica      PME--LNAASHSLRTANR---IDS-D---CLDYRHYSQHKFGRV---SSSLSDSRLQGN- 251
A0A0Q3MLL4_Amazona_aestiva                 PLE--LSVASHLLRTANR---IDS-D---CLDYRQCSQHKFGRV---SSSLSDTRLHGN- 256
A0A1V4J6C2_Patagioenas_fasciata_monilis    PLE--LNVASHTLRTANR---IDS-D---CMDYRYYSQHKFGRV---SSSLSDTRLHGN- 253
A0A2I0M3Z8_Columba_livia                   PLE--LNVASHTLRTANR---IDS-D---CMDYRYYSQHKFGRV---SSSLSDTRLHGN- 253
INP5E_Drosophila_melanogaster              PLQA--ATEPGDIKRATTPQHMSSPNIATQPDGIEHIEEQPGVRFMSHENMTLQRQGS-- 338


INP5E_Homo_sapiens                         --PLACDDCSLRSAKSS---FSLLAP----------IRSKDVRSRSYLEGSLLASGALLG 286
INP5E_Pan_troglodytes                      --PLACDDCSLRSAKSS---FSLLAP----------IRSKDVRSRSYLEGSLLASGALLG 286
A0A2K5K2A0_Colobus_angolensis_palliatus    --PLACDDCSLRSAKSS---FSLLAP----------IRSKDVRSRSYLEGSLLASGALLG 215
A0A2K6PY13_Rhinopithecus_roxellana         --PLACDDCSLRSAKSS---FSLLAP----------IRSKDVRSRSYLEGSLLASGALLG 287
H2PTZ0_Pongo_abelii                        --PLACDDCSLRSAKSS---FSLLAP----------IRSKDVRSRSYLEGSLLASGALLG 283
A0A2K6U2B2_Saimiri_boliviensis_boliviensis --PLACDDCSLRSARSS---FSLLAP----------IRTKDVRSRSYLEGSLLASGALLG 271
A0A2K6F2I5_Propithecus_coquereli           --PLACDSCSLRSAKSA---FSLLAP----------IRTKDVRSRSYLEGSLLASGALLG 284
A0A2K6KCW3_Rhinopithecus_bieti             --ESPCSGLGAASD-IL---WVLLLSLGTWPRPPPRDVLGSSRHRSYLEGSLLASGALLG 179
A0A2K5MDJ3_Cercocebus_atys                 --PLACDDCSLRSAK-S---FSLL---------AP-IRSKDVRSRSYLEGSLLASGALLG 285
A0A2K5XR75_Mandrillus_leucophaeus          ----------------------------------------MGLESYLEGSLLASGALLG 19
A0A0D9RTF7_Chlorocebus_sabaeus             --PLACDDYSLRSAKSS---FSLL---------AP-IRSKDVRSRSYLEGSLLASGALLG 286
A0A096NRS4_Papio_anubis                    --PLACDDCSLRSAKSS---FSLL---------AP-IRSKDVRSRSYLEGSLLASGALLG 285
```

```
A0A2K5U0T2_Macaca_fascicularis           --PLACDDCSLRSAKSS---FSLL---------AP-IRSKDVRSRSYLEGSLLASGALLG 286
F7DRP8_Macaca_mulatta                    --PLACDDCSLRSAKSS---FSLL---------AP-IRSKDVRSRSYLEGSLLASGALLG 286
A0A2K5PHS4_Cebus_capucinus_imitator      --PLACDDCSLRSAKSP---FSLLAP----------IRTKDVRSRSYLEGSLLASGALLG 280
A0A2K5CN95_Aotus_nancymaae               --PLACDDCSLRSAKSS---FSLLAP----------IRTKDVRSRSYLEGSLLASGALLG 288
A0A3Q0DNV4_Tarsius_syrichta              ---------------------------------------------SYLEGSLLASGALLG 15
H0XE73_Otolemur_garnettii                --PLACDSCSLRSANSA---FSLLTP----------IRTKDVRSRSYLEGSLLASGALLG 289
I3N2L4_Ictidomys_tridecemlineatus        --PLACDTHSLHSAKSF---FSLLAP----------IRTKDVRSRSYLEGSLLASGALLG 215
F7BT66_Callithrix_jacchus                --PLACDDCSLRSAKSS---FSLLAP----------IRTKDVRSRSYLEGSLLASGALLG 288
A0A250Y3V1_Castor_canadensis             --PLACDNHSLHSAKSS---FSLLAP----------IRTKDVRSRSYMEGSLLASGALLG 280
A0A1S3G993Dipodomys_ordii                --PLASDDHPLHAARSS---FSLLAP----------IRTKDIRSRSYLEGSLLASGALLG 232
H0VEZ3_Cavia_porcellus                   --PLTCDDHSLHSARSS---FSLLAP----------IRTKDVRSRSYLEGSLLASGALLG 288
G5B6B1_Heterocephalus_glaber             --PLACDDHSLHSAK-S---FSLLAP----------IRTKDVRSRSYLEGSLLASGALLG 292
A0A2Y9E841_Trichechus_manatus_latirostris --PLACDSCSLRSAKSS---FSLLAP----------IRANDVRSRSYLEGSLLASGALLG 267
L5KA82_Pteropus_alecto                   --PLACDDCSLRSAKSS---FSLLAP----------IRAKDVRSRSYLEGSLLASGALMG 262
A0A5N4EAQ5_Camelus_dromedarius           --PLANDDCSLHSAKST---FSLLAP----------IRAKDVRSRSYLEGSLLASGALMG 298
A0A3Q2HCU7_Equus_caballus                --PLACDNCSLRSAKSS---FSLLAP----------IRTKDVRSRSYLEGSLLASGALMG 295
A0A1S2ZM74_Erinaceus_europaeus           --PLSFDNCSLRSAKSS---FSLLAP----------IRAKDVRSRSYLEGSLLASGALMG 289
A0A3Q0DCZ7_Mesocricetus_auratus          --PLAGDDHSIHSARSS---FSLLAP----------IRTKDIRSRSYLEGSLMASGALLG 281
G3I6Z4_Cricetulus_griseus                --PLAGDDHSILSARSS---FSLLAP----------IRTKDIRSRSYLEGSLMASGALLG 281
INP5E_Rattus_norvegicus                  --PLAGDDHSIHSARSS---FSLLAP----------IRTKDIRSRSYLEGSLLASGALLG 290
INP5E_Mus_musculus                       --PLAGDDHSIHSAR-S---FSLLAP----------IRTKDIRSRSYLEGSLLASGALLG 289
A0A2Y9NRK2_Delphinapterus_leucas         --PLARDDCSLHSARSA---VSLLAP----------VRTKDVRSRSYLEGSLLASGALMG 269
A0A2Y9SXS0_Physeter_macrocephalus        --PLARDDCSLRPATST---FSLLVP----------IRTKDVRGRSYLEGSLLASGALMG 254
E1BAU6_Bos_taurus                        --PLAYDDCSLHSSRST---FSLLAP----------IRAKDVRSRSYLEGSLLATGALMG 298
A0A4W2BWU9_Bos_indicus_x_Bos_taurus      --PLAYDDCSLHSSRST---FSLLAP----------IRAKDVRSRSYLEGSLLATGALMG 298
L8IYW6_Bos_mutus                         --PLAYDDCSLHSSRST---FSLLAP----------IRAKDVRSRSYLEGSLLATGALMG 298
A0A452DQV4_Capra_hircus                  --PLAYDDCSLHSSRST---FSLLAP----------IRAKDVRSRSYLEGSLLASGALMG 298
A0A5J5MJZ6_Muntiacus_reevesi             --PLACDDCSLHSSRST---FSLLAP----------IRAKDVRSRSYLEGSLLASGALMG 298
A0A5N3WU40_Muntiacus_muntjak             --PLACDDCSLHSSRST---FSLLAP----------IRAKDVRSRSYLEGSLLASGALMG 298
A0A3Q7V225_Ursus_arctos_horribilis       --PLACDA------KSS---FSLLAP----------IRTKDVRSRSYLEGSLLASGALMG 284
A0A452Q8J6_Ursus_americanus              --PLACDA------KSS---FSLLAP----------IRTKDVRSRSYLEGSLLASGALMG 284
A0A452VEA9_Ursus_maritimus               --PLACDA------KSS---FSLLAP----------IRTKDVRSRSYLEGSLLASGALMG 284
A0A2U3ZR12_Odobenus_rosmarus_divergens   --PLACDA------K-S---FSLLAP----------IRAKDVRSRSYLEGSLLASGALMG 254
A0A3Q7N7C7_Callorhinus_ursinus           --PLACDA------K-S---FSLLAP----------IRAKDVRSRSYLEGSLLASGALMG 254
A0A2Y9HBQ8_Neomonachus_schauinslandi     --PLACDA------KSS---FSLLAP----------IRAKDVRSRSYLEGSLLASGALMG 255
A0A485NC41_Lynx_pardinus                 --PLACDA------KPS---FSLLAP----------IRAKDVRSRSYLEGSLLASGALMG 278
A0A3Q7QUW1_Vulpes_vulpes                 --PLVCEA------RSS---FSLLAP----------IRAKDVRSRSYLEGSLLASGALMG 255
F1PDA7_Canis_lupus_familiaris            --PLVCEA------RSS---FSLLAP----------IRAKDVRSRSYLEGSLLASGALMG 264
A0A2Y9JFT0_Enhydra_lutris_kenyoni        --PLASEA------RSS---FSLLAP----------IRAKDVRTRSYLEGSLLASGALMG 284
M3YJ90_Mustela_putorius_furo             --PLASEA------RSS---FSLLAP----------IRAKDVRTRSYLEGSLLASGALMG 284
I3JX61_Oreochromis_niloticus             --NMGSDSASVNSMRST---YSVLSP----------IRPQDMRNRSFMEGSVLGSGALLG 283
A0A3Q4H5I7_Neolamprologus_brichardi      --NMGSDSASVNSMRST---YSVLSP----------IRPQDMRNRSFMEGSVLGSGALLG 283
A0A3B4FRA1_Pundamilia_nyererei           --NMGSDSASVNSMRST---YSVLSP----------IRPQDMRNRSFMEGSVLGSGALLG 283
A0A3P8QQM7_Astatotilapia_calliptera      --NMGSDSASVNSMRST---YSVLSP----------IRPQDMRNRSFMEGSVLGSGALLG 283
W5M6X5_Lepisosteus_oculatus              --SMVSDNTSLSSMKST---LSVLNP----------IRPKDVRNRSFLEGSVLGHGALLG 234
A0A3B3RRU9_Paramormyrops_kingsleyae      --TMVSDNTSLNSMKST---SSVLNP----------IRPRDVRNRSFLEGSVLGHGALLG 284
A0A4W4HDW3_Electrophorus_electricus      --TMVLDSTSVNSMKST---LSALNP----------IRPKDVRNRSFLEGSVLGSGALLG 281
A0A3Q3IZN0_Monopterus_albus              --NMGSDSASVNSLRST---YSVLSP----------IRPQDVRNRSFLEGSVLGRGALLG 281
A0A3P8VEE4_Cynoglossus_semilaevis        --NMGSDNASVNSLRST---YSVLSP----------IRPQDVRNRSFLEGSLMGSGALLG 274
A0A3Q1H6D9_Anabas_testudineus            --NMGSDSASVNSLRSN---YSVLSP----------IRPQDVRNRSFLEGSVLGSGALLG 284
A0A4W6CEK3_Lates_calcarifer              --NMGSDSASVNSMRST---YSVLSP----------IRPQDVRNRSFLEGSVLGSGALLG 284
A0A3B4VK37_Seriola_dumerili              --NMGSDSASVNSMRST---YSVLSP----------IRPQDVRNRSFLEGSVLGSGALLG 284
A0A3Q3GNS7_Labrus_bergylta               --NMGSDSASVNSMRSN---YSVLSP----------IRPQDVRNRSFLEGSVLGSGALLG 283
A0A3Q3RRU7_9TELE_Mastacembelus_armatus   --NMGSDSASVNSLRST---YSVLSP----------IRPQDVRNRSFLEGSVLGNGALLG 278
A0A4U5UTG8_Collichthys_lucidus           --NMGSDSASVNSMRST---YSVLSP----------IRPQDVRNRSFLEGSGLGSGALLG 287
A0A3B4ZI99_Stegastes_partitus            --NMGSDSASVNSMRST---YSVLSP----------IRPQDVRNRSFLEGSVLGSGALLG 287
A0A3Q1H1R6_Acanthochromis_polyacanthus   --NMGSDSASVNSMRST---YSVLSP----------IRPQDVRNRSFLEGSVLGSGALLG 292
A0A3Q1CXU7_Amphiprion_ocellaris          --NMGSDSASVNSMRST---YSVLSP----------IRPQDVRNRSFLEGSVLGSGALLG 285
A0A3P8SMU2_Amphiprion_percula            --NMGSDSASVNSMRST---YSVLSP----------IRPQDVRNRSFLEGSVLGSGALLG 288
A0A5J5DH05_Etheostoma_spectabile         --NMGSDSASVNSMRST---YSVLSP----------IRPQDVRNRSFLEGSVLGSGALLG 285
A0A484DDW2_Perca_flavescens              --NMGSDSASVNSMRST---YSVLSP----------IRPQDVRNRSFLEGSVLGSGALLG 285
A0A4Z2H351_Liparis_tanakae               --TMGSDSASVTSMRST---YSVLSP----------IRPQDVRNRSFLEGSVLGSGALLG 283
W5UJJ6_Ictalurus_punctatus               --TMVSDRTSVNSMKSA---FSVLNP----------IRPKDVRNRSFLEGSVMGNGALLG 285
A0A5N5KDW8_Pangasianodon_hypophthalmus   --TMVSDSTSVNSMKST---FSVLNP----------IRPKDVRNRSFLEGSVLGNGALLG 253
A0A3P8ZCA3_Esox_lucius                   --SMVSDNTSVNSMKST---FSVLNP----------IRPRDVRNRSFLEGSVLGSGALLG 290
A0A4W5L9K3_Hucho_hucho                   --NMVLDNTSVNSMKST---FSVLNP----------ITPRDVRNRSFLEGSVLGSGALLG 291
A0A1S3NKU9_Salmo_salar                   --NMVLDNTSVNSMKST---FSVLNP----------IRPRDVRNRSFLEGSVLGSGALLG 296
W5L4Z3_Astyanax_mexicanus                --TMVADSASVNSMKSN---FSALNP----------IRPKDVRNRSFLEGSVLGNGALLG 284
A0A3B4C5P1_Pygocentrus_nattereri         --TMVSDSASVNSMKST---FSALNP----------IRPKDVRNRSFLEGSVLGNGALLG 286
H3BEM7_Latimeria_chalumnae               --SMVPDNSSVSSMKSS---STMRFP----------ILCKDIRKRNYLEGSLLSTGALLG 333
A0A1L8F610_Xenopus_laevis                SSSMVCNQCSSDSMKST---YSLLTP----------IRPKDMRNRSYLEGSLLASGALLG 270
K7FPA9_Pelodiscus_sinensis               --GMVYDSCSTDSMKST---FSLLTP----------LRAKDVRNRSYLEGSLLASGALLG 298
A0A452GIT0_Gopherus_agassizii            --GMVCDSCSTDSMKST---FSLLTP----------LRAKDVRNRSYLEGSLLASGALLG 299
G3VXX0_Sarcophilus_harrisii              --SVVCDNCSTTSVKSS---FSLLTP----------IHSKDIRSRAYMEGSLLTSGALLG 299
A0A4X2KFU4_Vombatus_ursinus              --CMVCDNCSTTSLKSS---FSLLTP----------IHSKNVRSRSYMEGSLLASGALLG 299
G1N6K2_Meleagris_gallopavo               --GTVYDNCSTDSMKST---FSLLTP----------IRSKDVRSRSYLEGSLLASGALLG 233
F1P5J6_Gallus_gallus                     --GIVYDNCSTDSMKST---FSLLTP----------IRSKDVRSRSYLEGSLLASGALLG 301
U3IH52_Anas_platyrhynchos_platyrhynchos  --GMVYDNCSTDSMKST---FSLLTP----------IRSKDVRSRSYLEGSLLANGALLG 281
A0A151MFV8_Alligator_mississippiensis    --GMVCDSCSTDSMKSS---FSLLAP----------IRAKDVRSRSYLEGSLLASGALLG 92
A0A1U7RBH0_Alligator_sinensis            --GMVCDSCSTDSMKSS---FSLLAP----------IRAKDVRSRSYLEGSLLASGALLG 260
U3JGA7_Ficedula_albicollis               --GTFYDNCSTDSMKST---FSVLTP----------IRAKDVRSRSYLEGSLLASGALMG 296
```

```
A0A3L8SD89_Chloebia_gouldiae              --GTLYDNCSTDSMKST---FSVLTP----------IRAKDVRSRSYLEGSLLASGALMG 278
H0Z776_Taeniopygia_guttata                --GTLYDNCSTDSMKST---FSVLTP----------IRAKDVRSRSYLEGSLLASGALMG 236
A0A218V5X6_Lonchura_striata_domestica     --GTLYDNCSTDSMKST---FSVLTP----------IRAKDVRSRSYLEGSLLASGALMG 296
A0A0Q3MLL4_Amazona_aestiva                --GMVYDNCSTDSMKST---FSLLTP----------IRSKDVRSRSYLEGSLLASGALLG 301
A0A1V4J6C2_Patagioenas_fasciata_monilis   --GMVYDNCSTDSMKST---FSLLTP----------IRSKDVRSRSYLEGSLLASGALLG 298
A0A2I0M3Z8_Columba_livia                  --GMVYDNCSTDSMKST---FSLLTP----------IRSKDVRSRSYLEGSLLASGALLG 298
INP5E_Drosophila_melanogaster             ---------GSNLARQTLMAAHALNL----------IPADNARERSYLDGR-LGSTSLLG 378
                                                                                    :::*  :    :*:*

INP5E_Homo_sapiens                        ADELARYFPDRNVALFVATWNMQGQKELPPS----LDEFLLPAEADYAQDLYVIGVQEGC 342
INP5E_Pan_troglodytes                     ADELARYFPDRNVALFVATWNMQGQKELPPS----LDEFLLPAEADYAQDLYVIGVQEGC 342
A0A2K5K2A0_Colobus_angolensis_palliatus   ADELARYFPDRNVALFVATWNMQGQKELPPS----LDEFLLPAEADYAQDLYIIGVQEGC 271
A0A2K6PY13_Rhinopithecus_roxellana        ADELARYFPDRNVALFVATWNMQGQKELPPS----LDEFLLPAEADYAQDLYVIGVQEGC 343
H2PTZ0_Pongo_abelii                       ADELARYFPDRNVALFVATWNMQGQKELPPS----LDEFLLPAEADYAQDLYVIGVQEGC 339
A0A2K6U2B2_Saimiri_boliviensis_boliviensis AEELARYFPDRNVALFVATWNMQGQKELPPS----LDEFLLPAEADYAQDLYAIGVQEGC 327
A0A2K6F2I5_Propithecus_coquereli          ADELARYFPDRNVALFVATWNMQGQKELPPS----LDELLLPAEADYAQDLYVVGVQEGC 340
A0A2K6KCW3_Rhinopithecus_bieti            ADELARYFPDRNVALFVATWNMQGQKELPPS----LDEFLLPAEADYAQDLYVIGVQEGC 235
A0A2K5MDJ3_Cercocebus_atys                ADELARYFPDRNVALFVATWNMQGQKELPPS----LDEFLLPAEADYAQDLYVVGVQEGC 341
A0A2K5XR75_Mandrillus_leucophaeus         ADELARYFPDRNVALFVATWNMQGQKELPPS----LDEFLLPAEADYAQDLYVIGVQEGC 75
A0A0D9RTF7_Chlorocebus_sabaeus            ADELARYFPDRNVALFVATWNMQGQKELPPS----LDEFLLPAEADYAQDLYVIGVQEGC 342
A0A096NRS4_Papio_anubis                   ADELARYFPDRNVALFVATWNMQGQKELPPS----LDEFLLPAEADYAQDLYVIGVQEGC 341
A0A2K5U0T2_Macaca_fascicularis            ADELARYFPDRYVALFVATWNMQGQKELPPS----LDEFLLPAEADYAQDLYVIGVQEGC 342
F7DRP8_Macaca_mulatta                     ADELARYFPDRYVALFVATWNMQGQKELPPS----LDEFLLPAEADYAQDLYVIGVQEGC 342
A0A2K5PHS4_Cebus_capucinus_imitator       AEELARYFPDRNVALFVATWNMQGQKELPPS----LDEFLLPAEADYAQDLYAIGVQEGC 336
A0A2K5CN95_Aotus_nancymaae                AEELARYFPDRNVALFAATWNMQGQKELPPS----LDEFLLPAEADYAQDLYAIGVQEGC 344
A0A3Q0DNV4_Tarsius_syrichta               ADELARYFPDRTVAVFVATWNMQGQKELPPS----LDEFLLPAEADYAQDLYVIGVQEGC 71
H0XE73_Otolemur_garnettii                 AEELARYFPDRNMALFVATWNMQGQKELPPS----LDEFLLPAEADYAQDLYVVGVQEGC 345
I3N2L4_Ictidomys_tridecemlineatus         ADELTQYFPDRNMALFVATWNMQGQKELPPS----LDEFLLPTEADYTQDLYVIGVQEGC 271
F7BT66_Callithrix_jacchus                 AEELARYFPDRNVALFVATWNMQGQKELPPS----LDEFLLPAEADYAQDLYAIGVQEGC 344
A0A250Y3V1_Castor_canadensis              AEELARYFPDRNVVLFVATWNMQGQKELPPS----LDEFLLPTEADYTQDLYVIGVQEGC 336
A0A1S3G993Dipodomys_ordii                 ADELARYFPDRNVALFVATWNMQGQKELPVS----LDEFLLPSEADYTQDLYVIGVQEGC 288
H0VEZ3_Cavia_porcellus                    AEELAQYFPDRSMALFVATWNMQGQKELPPS----LDELLLPPEADYTQDLYVIGVQEGC 344
G5B6B1_Heterocephalus_glaber              ADELAQYFPDRSVALFVVTWNMQGQKELPPS----LDELLLPAAADYTQDLYVVGVQEGC 348
A0A2Y9E841_Trichechus_manatus_latirostris ADELARYFPDRSVAVFVATWNMQGQKELPPN----LDELLLPTEADYAQDMYVVGVQEGC 323
L5KA82_Pteropus_alecto                    ADELARYFPDRSVALFVATWNMQGQKEPPSN----LDELLLPAEADYAQDLYVVGVQEGC 318
A0A5N4EAQ5_Camelus_dromedarius            ADELARYFPDRSVALFVATWNMQGQKELPPN----LDELLLPAEADYAQDLYVVGVQEGC 354
A0A3Q2HCU7_Equus_caballus                 ADELARYFPDRNMALYVATWNMQGQKELPPN----LDELLLPVEADYAQDLYVVGVQEGC 351
A0A1S2ZM74_Erinaceus_europaeus            AEELARYFPDRNLALFVATWNMQGQKELPTN----LDELLLPAEADFAQDLYVVGVQEGC 345
A0A3Q0DCZ7_Mesocricetus_auratus           ADELARYFPDRNMALFVATWNMQGQKELPAS----LDEFLLPAEADYTQDLYVIGVQEGC 337
G3I6Z4_Cricetulus_griseus                 ADELVRYFPDRNMALFVATWNMQGQKELPAS----LDEFLLPTEADYTQDLYVIGVQEGC 337
INP5E_Rattus_norvegicus                   ADELARYFPDRNMALFVATWNMQGQKELPAS----LDEFLLPTEADYTQDLYVIGVQEGC 346
INP5E_Mus_musculus                        AEELARYFPDRNMALFVATWNMQGQKELPAS----LDEFLLPTEADYTQDLYVIGIQEGC 345
A0A2Y9NRK2_Delphinapterus_leucas          ADELARYFPDRNVALFVATWNMQGQKELPPN----LDELLLPAEADFAQDLYVVGVQEGC 325
A0A2Y9SXS0_Physeter_macrocephalus         ADELARYFPDRNVALFVATWNMQGQKELPPN----LDELLLPAEADLAQDLYVVGVQEGC 310
E1BAU6_Bos_taurus                         ADELARYFPDRSVALFVATWNMQGQKELPPN----LDELLLPAEADLAQDLYVVGVQEGC 354
A0A4W2BWU9_Bos_indicus_x_Bos_taurus       ADELARYFPDRSVALFVATWNMQGQKELPPN----LDELLLPAEADLAQDLYVVGVQEGC 354
L8IYW6_Bos_mutus                          ADELARYFPDRSVALFVATWNMQGQKELPPN----LDELLLPAEADLAQDLYVVGVQEGC 354
A0A452DQV4_Capra_hircus                   ADELARYFPDRSVALFVATWNMQGQKELPPN----LDELLLPAEADLAQDLYVVGVQEGC 354
A0A5J5MJZ6_Muntiacus_reevesi              ADELARYFPDRTVALFVATWNMQGQKELPPN----LDELLLPAEADLAQDLYVVGVQEGC 354
A0A5N3WU40_Muntiacus_muntjak              ADELARYFPDRTVALFVATWNMQGQKELPPN----LDELLLPAEADLAQDLYVMGVQEGC 354
A0A3Q7V225_Ursus_arctos_horribilis        AEELARYFPDRNMALFVATWNMQGQKELPPN----LDELLLPAEADYAQDLYVVGVQEGC 340
A0A452Q8J6_Ursus_americanus               AEELARYFPDRNMALFVATWNMQGQKELPPN----LDELLLPAEADYAQDLYVVGVQEGC 340
A0A452VEA9_Ursus_maritimus                AEELARYFPDRNMALFVATWNMQGQKVSRSRPLGFSAGLSGPHAADYAQDLYVVGVQEGC 344
A0A2U3ZR12_Odobenus_rosmarus_divergens    AEELARYFPDRNMALFVATWNMQGQKELPPN----LDELLLPAEADYAQDLYVVGVQEGC 310
A0A3Q7N7C7_Callorhinus_ursinus            AEELARYFPDRNMALFVATWNMQGQKELPPN----LDELLLPAEADYAQDLYVVGVQEGC 310
A0A2Y9HBQ8_Neomonachus_schauinslandi      AEELARYFPDRNMALFVATWNMQGQKELPPN----LDELLLPAEADYAQDLYVVGVQEGC 311
A0A485NC41_Lynx_pardinus                  ADELARYFPDRSMAVFVATWNMQGQKELPPN----LDELLLPAEADYAQDLYVIGVQEGC 334
A0A3Q7QUW1_Vulpes_vulpes                  AEELARYFPDRNLALFVATWNMQGQKELPPN----LDELLLPAEADYAQDLYVIGVQEGC 311
F1PDA7_Canis_lupus_familiaris             AEELARYFPDRNLALFVATWNMQGQKELPPN----LDELLLPAEADYAQDLYVIGVQEGC 320
A0A2Y9JFT0_Enhydra_lutris_kenyoni         AEELARYFPDRSMALFVATWNMQGQKELPPN----LDELLLPAEADYAQDLYVVGVQEGC 340
M3YJ90_Mustela_putorius_furo              AEELARYFPDRSMALFVATWNMQGQKELPPN----LDELLLPAEADYAQDLYVVGVQEGC 340
I3JX61_Oreochromis_niloticus              AEELDRLFPDRRVGIYIATWNMQGEKGLPVN----LDDLLLPTDSEFAQDFYVIGVQEGC 339
A0A3Q4H5I7_Neolamprologus_brichardi       AEELDRLFPDRRVGIYIATWNMQGEKGLPVN----LDDLLLPTDSEFAQDFYVIGVQEGC 339
A0A3B4FRA1_Pundamilia_nyererei            AEELDRLFPDRRVGIYIATWNMQGEKGLPVN----LDDLLLPTDSEFAQDFYVIGVQEGC 339
A0A3P8QQM7_Astatotilapia_calliptera       AEELDRLFPDRRVGIYIATWNMQGEKGLPVN----LDDLLLPTDSEFAQDFYVIGVQEGC 339
W5M6X5_Lepisosteus_oculatus               AEELDRYFPDRRVGIYIATWNMQGKKELPAN----LDDLLLPADSEFAQDIYFIGVQEGC 290
A0A3B3RRU9_Paramormyrops_kingsleyae       ADELDRYFPERRLGIYIVTWNMQGEKELPAD----LDDLLLPTDSEFAQDFYVIGVQEGC 340
A0A4W4HDW3_Electrophorus_electricus       AEELDRYFPDRHVGICIATWNMQGEKGLPSS----LDDLLLPTDMEFAQDFYIIGVQEGC 337
A0A3Q3IZN0_Monopterus_albus               AEELDRHFPNRRVGIYVVTWNMQGENGMPAN----LDDLLLPMDSEFAQDFYIIGVQEGC 337
A0A3P8VEE4_Cynoglossus_semilaevis         AEELDRYFPDRKLSIYIVTWNMQGEKGLPVN----LDDLLLPTDSDTAQDFYIIGVQEGC 330
A0A3Q1H6D9_Anabas_testudineus             AEELDRYFPDRRVGFYIATWNMQGEKGLPAN----LDDLLLPTDSEFAQDFYVIGVQEGC 340
A0A4W6CEK3_Lates_calcarifer               AEELDRYFPNRRVGVYIATWNMQGEKGLPAN----LDDLLLPTDSEFAQDFYIIGVQEGC 340
A0A3B4VK37_Seriola_dumerili               AEELDRYFPDRRVGIYIATWNMQGERGLPTN----LDDLLLPTDSEFAQDFYIIGVQEGC 340
A0A3Q3GNS7_Labrus_bergylta                AEELDRYFPDRRLGIYVATWNMQGEKGLPSN----LDDLLLPTDSEFAQDFYIIGVQEGC 339
A0A3Q3RRU7_9TELE_Mastacembelus_armatus    AEELDRYFPDRRLGIYIATWNMQGEKGLPPN----LDDFLLPTDSEFAQDFYIIGVQEGC 334
A0A4U5UTG8_Collichthys_lucidus            AEELDRYFPDRKVGIYIATWNMQGEKGLPGN----LDDLLLPTDSEFAQDFYIIGVQEGC 343
A0A3B4ZI99_Stegastes_partitus             AEELDRYFPDRRVGVYVATWNMQGEKGLPTN----LDDLLLPTDSEFAQDFYIIGVQEGC 343
A0A3Q1H1R6_Acanthochromis_polyacanthus    AEELDRYFPDRRVGVYIATWNMQGEKGLPTN----LDDLLLPTDSEFAQDFYIIGVQEGC 348
A0A3Q1CXU7_Amphiprion_ocellaris           AEELDRYFPDRRVGVYIATWNMQGEKGLPTN----LDDLLLPTDSEFAQDFYIIGVQEGC 341
```

```
A0A3P8SMU2_Amphiprion_percula           AEELDRYFPDRRVGVYIATWNMQGEKGLPTN----LDDLLLPTDSEFAQDFYIIGVQEGC 344
A0A5J5DH05_Etheostoma_spectabile        AEELDRHFPDRRVGIYIATWNMQGEKGLPAN----LDDLLLPTDSAFAQDFYIIGVQEGC 341
A0A484DDW2_Perca_flavescens             AEELDRHFPDRRVGIYIATWNMQGEKGLPAN----LDDLLLPTDSAFAQDFYIIGVQEGC 341
A0A4Z2H351_Liparis_tanakae              AEELDRHFPNRRVGIYVATWNMQGEKGLPSN----LDDLLLPTDSEFAQDFYIIGVQEGC 339
W5UJJ6_Ictalurus_punctatus              AEELDRYFPSRQIGIYVATWNMQGEKGLPNN----LDDLLLPTDTDFAQDFYIIGVQEGC 341
A0A5N5KDW8_Pangasianodon_hypophthalmus  AEELDRYFPSRQIGIYIATWNMQGEKGLPNN----LDDLLLPTDTDFAQDFYIIGVQEGC 309
A0A3P8ZCA3_Esox_lucius                  AEELDRYFPERRVGIYIATWNMQGEKGLPNN----LDDLLLPTDSEFAQDFYIIGVQEGC 346
A0A4W5L9K3_Hucho_hucho                  AEELDRYFPERRVGIYIATWNMHGEKGLPNN----LDDLLLPTDSEFAQDFYIIGVQEGC 347
A0A1S3NKU9_Salmo_salar                  AEELDRYFPERRVGIYIATWNMHGEKGLPNN----LDDLLLPTDSEFAQDFYVIGVQEGC 352
W5L4Z3_Astyanax_mexicanus               PEELDRYFPDRHVGIYIATWNMQGEKGFPNN----LDDLLLPTDTEFAQDFYIIGVQEGC 340
A0A3B4C5P1_Pygocentrus_nattereri        PEELDRYFPDRHVGIYIATWNMQGEKGLPNN----LDDLLLPTDTEFAQDFYIIGVQEGC 342
H3BEM7_Latimeria_chalumnae              SEELDRHFPNRKIGIYVVTWNMQGRKELPEN----LNDLLLPSDSDFAQDMYVVGTQESC 389
A0A1L8F610_Xenopus_laevis               AEELERYFPDRRLAIFIATWNMQGKKELPES----LDDFLLPSDDDFAQDVYVIGVQEGC 326
K7FPA9_Pelodiscus_sinensis              SEELSRYFPDRNIGIFVATWNMQGQKELPEN----LDDFLFPSDPDYAQDMYVIGVQEGC 354
A0A452GIT0_Gopherus_agassizii           AEELSRYFPDRNIGIFVATWNMQGRKELPEN----LDDFLFPSDPDYAQDMYVIGIQEGC 355
G3VXX0_Sarcophilus_harrisii             ANELTRYFPDRNMGIFVATWNMQGQKELPNN----LDDFLLPSEADYVQDMYVLGVQEGC 355
A0A4X2KFU4_Vombatus_ursinus             ADELTRYFPDGNLGLFVATWNMQGQKELPNN----LDDFLLPSEADYVQDMYVLGVQEGC 355
G1N6K2_Meleagris_gallopavo              AEELSRYFPDRNIGIFVATWNMQGQKELPEN----LDDFLLPTDPDYAQDMYVIGVQEGC 289
F1P5J6_Gallus_gallus                    AEELSRYFPDRNVGIFVATWNMQGQKELPEN----LDDFLLPTDPDYAQDMYVIGVQEGC 357
U3IH52_Anas_platyrhynchos_platyrhynchos AEELSRYFPDRNIGIFVATWNMQGQKELPVN----LDDFLLPTDPDYAQDMYVIGVQEGC 337
A0A151MFV8_Alligator_mississippiensis   AEELNRYFPDRTIGIFVATWNMQGQKELPEN----LDDLLLPTDPDYAQDMYIIGVQEGC 148
A0A1U7RBH0_Alligator_sinensis           AEELNRYFPDRTIGIFVATWNMQGQKELPEN----LDDLLLPTDPDYAQDMYIIGVQEGC 316
U3JGA7_Ficedula_albicollis              AEELSRYFPDRNMGIFVATWNMQGQKELPVN----LDDFLLPTDPDFAQDMYVIGVQEGC 352
A0A3L8SD89_Chloebia_gouldiae            AEELSRYFPNRNMGIFVATWNMQGQKELPVN----LDDFLLPTDPDFAQDMYVIGVQEGC 334
H0Z776_Taeniopygia_guttata              AEELSRYFPNRNMGIFVATWNMQGQKELPVN----LDDFLLPTDPDFAQDMYVIGVQEGC 292
A0A218V5X6_Lonchura_striata_domestica   AEELSRYFPNRNMGIFVATWNMQGQKELPVN----LDDFLLPTDPDFAQDMYVIGVQEGC 352
A0A0Q3MLL4_Amazona_aestiva              AEELSRYFPDRNIGIFVATWNMQGQKELPLN----LDDFLLPADPDYAQDMYVIGVQEGC 357
A0A1V4J6C2_Patagioenas_fasciata_monilis AEELSRYFPDRNIGIFVATWNMQGQKELPVN----LDDFLLPTEADYAQDMYVIGVQEGC 354
A0A2I0M3Z8_Columba_livia                AEELSRYFPDRNIGIFVATWNMQGQKELPVN----LDDFLLPTEADYAQDMYVIGVQEGC 354
INP5E_Drosophila_melanogaster           PSELSRVLPQKEITVFVGTWNMNGHS-PPKQ----LNDFVLPANVEHVPDIVVMGTQEST 433
                                         .** : :*.  : .   ****:*.            :  *      . *.  :* **.

INP5E_Homo_sapiens                      SDRREWETRLQETLGPHYVLLSSAAHGVLYMSLFIRRDLIWFCS-EVECSTVTTRIVSQI 401
INP5E_Pan_troglodytes                   SDRREWETRLQETLGPHYVLLSSAAHGVLYMSLFIRRDLIWFCS-EVECSTVTTRIVSQI 401
A0A2K5K2A0_Colobus_angolensis_palliatus SDRREWETRLQETLGPHYVLLSSAAHGVLYMSLFIRRDLIWFCS-EVECSTVTTRIVSQI 330
A0A2K6PY13_Rhinopithecus_roxellana      SDRREWETRLQETLGPHYVLLSSAAHGVLYMSLFIRRDLIWFCS-EVECSTVTTRIVSQI 402
H2PTZ0_Pongo_abelii                     SDRREWETRLQETLGPHYVLLSSAAHGVLYMSLFIRRDLIWFCS-EVECSTVTTRIVSQI 398
A0A2K6U2B2_Saimiri_boliviensis_boliviensis SDRREWETRLQETLGPHYVLIASAAHGVLYMALFIRRDLIWFCS-EVESSTVTTRIVSQI 386
A0A2K6F2I5_Propithecus_coquereli        SDRREWETRLQETLGPHYVLLSSAAHGVLYLSLFIRRDLIWFCS-EVECSTVTTRIVSQI 399
A0A2K6KCW3_Rhinopithecus_bieti          SDRREWETRLQETLGPHYVLLSSAAHGVLYMSLFIRRDLIWFCS-EVECSTVTTRIVSQI 294
A0A2K5MDJ3_Cercocebus_atys              SDRREWETRLQETLGPHYVLLSSAAHGVLYMSLFIRRDLIWFCS-EVECSTVTTRIVSQI 400
A0A2K5XR75_Mandrillus_leucophaeus       SDRREWETRLQETLGPHYVLLSSAAHGVLYMSLFIRRDLIWFCS-EVECSTVTTRIVSQI 134
A0A0D9RTF7_Chlorocebus_sabaeus          SDRREWETRLQETLGPHYVLLSSAAHGVLYMSLFIRRDLIWFCS-EVECSTVTTRIVSQI 401
A0A096NRS4_Papio_anubis                 SDRREWETRLQETLGPHYVLLSSAAHGVLYMSLFIRRDLIWFCS-EVECSTVTTRIVSQI 400
A0A2K5U0T2_Macaca_fascicularis          SDRREWETRLQETLGPHYVLLSSAAHGVLYMSLFIRRDLIWFCS-EVECSTVTTRIVSQI 401
F7DRP8_Macaca_mulatta                   SDRREWETRLQETLGPHYVLLSSAAHGVLYMSLFIRRDLIWFCS-EVECSTVTTRIVSQI 401
A0A2K5PHS4_Cebus_capucinus_imitator     SDRREWETRLQETLGPHYVLIASAAHGVLYMALFIRRDLIWFCS-EVESSTVTTRIVSQI 395
A0A2K5CN95_Aotus_nancymaae              SDRREWETRLQETLGPHYVLIASAAHGVLYMALFIRRDLIWFCS-EVESSTVTTRIVSQI 403
A0A3Q0DNV4_Tarsius_syrichta             SDRREWEARLQETLGPHYVLLSSAAHGVLYMSLFIRRDLIWFCS-EVECATVTTRIVSQI 130
H0XE73_Otolemur_garnettii               SDRREWETRLQETLGPHYVLLSSAAHGVLYMSLFLRRDLIWFCSAEVESSTVTTRIVSQI 405
I3N2L4_Ictidomys_tridecemlineatus       SDRREWETRLQETLGPRYVLLSSAAHGVLYVSLFIRRDLIWFCS-EVECSTVTTRIVSQI 330
F7BT66_Callithrix_jacchus               SDRREWETRLQETLGPHYVLIASAAHGVLYMALFIRRDLIWFCS-EVESSTVTTRIVSQI 403
A0A250Y3V1_Castor_canadensis            SDRREWETRLQETLGPRYVLLSSAAHGVLYMSLFIRRDLIWFCS-EVECSTVTTRIVSQI 395
A0A1S3G993Dipodomys_ordii               SDRREWETRLQETLGPRYVLLSSAAHGVLYMSLFIRRDLIWFCS-EVECSTVTTRIVSQI 347
H0VEZ3_Cavia_porcellus                  SDRREWETRLQETLGPRYVLLSSAAHGVLYLSLFIRRDLIWFCS-EVECATVTTRIVSQI 403
G5B6B1_Heterocephalus_glaber            SDRREWEVRLQETLGPRYVLLSSTAHGVLYLCLFIRRDLIWFCS-EVECATVTTRIVSQI 407
A0A2Y9E841_Trichechus_manatus_latirostris SDRREWEVRLQETLGPHYVLLQSAAHGVLYLSVFLRRDLIWFCS-EVECSTVTTRIVSHI 382
L5KA82_Pteropus_alecto                  SDRREWETRLQETLGPHYVMLCSAAHGALHMSVLIRRDLVWFCS-EVESSTVTTRIVSQI 377
A0A5N4EAQ5_Camelus_dromedarius          SDRREWEARLQETLGPHYVMLFSTAHGALYLSVLIRRDLIWFCS-EVESATVTTRIVSQI 413
A0A3Q2HCU7_Equus_caballus               SDRREWETRLQETLGPHYVMLYSAAHGALYMSVLIRRDLIWFCS EVESSTVTTRIVSQI 410
A0A1S2ZM74_Erinaceus_europaeus          SDRREWETRLQETLGPHYVMLYSTAHGALYMSVLIRRDLIWFCS EVESSTVTTRIVSQI 404
A0A3Q0DCZ7_Mesocricetus_auratus         SDRREWETRLQETLGPQYVLLSSAAHGVLYMSLFIRRDLIWFCS EVECSTVTTRIVSQI 396
G3I6Z4_Cricetulus_griseus               SDRREWETRLQETLGPQYVLLSSAAHGVLYMSLFIRRDLIWFCS EVECSTVTTRIVSQI 396
INP5E_Rattus_norvegicus                 SDRREWETRLQETLGPQYVLLSSAAHGVLYMSLFIRRDLIWFCS EVEYSTVTTRIVSQI 405
INP5E_Mus_musculus                      SDRREWETRLQETLGPQYVLLSSAAHGVLYMSLFIRRDLIWFCS EVEYSTVTTRIVSQI 404
A0A2Y9NRK2_Delphinapterus_leucas        SDRREWETRLQETLGPHYVMLYSAAHGALYMSVLIRRDLIWFCS EVESSTVTTRIVSQI 384
A0A2Y9SXS0_Physeter_macrocephalus       SDRREWETRLQETLGPHYVMLYSAAHGALYMSVLIRRDLIWFCS EVESSTVTTRIVSQI 369
E1BAU6_Bos_taurus                       SDRREWEARLQETLGPHYVMLYSAAHGALYMAVLIRRDLIWFCS EVESSTVTTRIVSQI 413
A0A4W2BWU9_Bos_indicus_x_Bos_taurus     SDRREWEARLQETLGPHYVMLYSAAHGALYMAVLIRRDLIWFCS EVESSTVTTRIVSQI 413
L8IYW6_Bos_mutus                        SDRREWEVRLQETLGPHYVMLYSAAHGALYMAVLIRRDLIWFCS EVESSTVTTRIVSQI 413
A0A452DQV4_Capra_hircus                 SDRREWEARLQETLGPHYVMLYSAAHGALYMAVLIRRDLIWFCS EVESSTVTTRIVSQI 413
A0A5J5MJZ6_Muntiacus_reevesi            SDRREWETRLQETLGPHYVMLYSAAHGALYMSVLIRRDLIWFCS EVESSTVTTRIVSQI 413
A0A5N3WU40_Muntiacus_muntjak            SDRREWETRLQETLGPHYVMLYSAAHGALYMSVLIRRDLIWFCS EVESSTVTTRIVSQI 413
A0A3Q7V225_Ursus_arctos_horribilis      SDRREWETRLQETLGPHYVTLYSAAHGALYMSVLIRRDLIWFCS EVESSTVTTRIVSHI 399
A0A452Q8J6_Ursus_americanus             SDRREWETRLQETLGPHYVTLYSAAHGALYMSVLIRRDLIWFCS EVESSTVTTRIVSHI 399
A0A452VEA9_Ursus_maritimus              SDRREWETRLQETLGPHYVTLYSAAHGALYMSVLIRRDLIWFCS EVESSTVTTRIVSHI 403
A0A2U3ZR12_Odobenus_rosmarus_divergens  SDRREWETRLQETLGPHYVTLYSAAHGALYMSVLIRRDLIWFCS EVESSTVTTRIVSHI 369
A0A3Q7N7C7_Callorhinus_ursinus          SDRREWETRLQETLGPHYVTLYSAAHGALYMSVLIRRDLIWFCS EVESSTVTTRIVSHI 369
A0A2Y9HBQ8_Neomonachus_schauinslandi    SDRREWETRLQETLGPRYVTLYSAAHGALYMSVLIRRDLIWFCS EVESSTVTTRIISHI 370
```

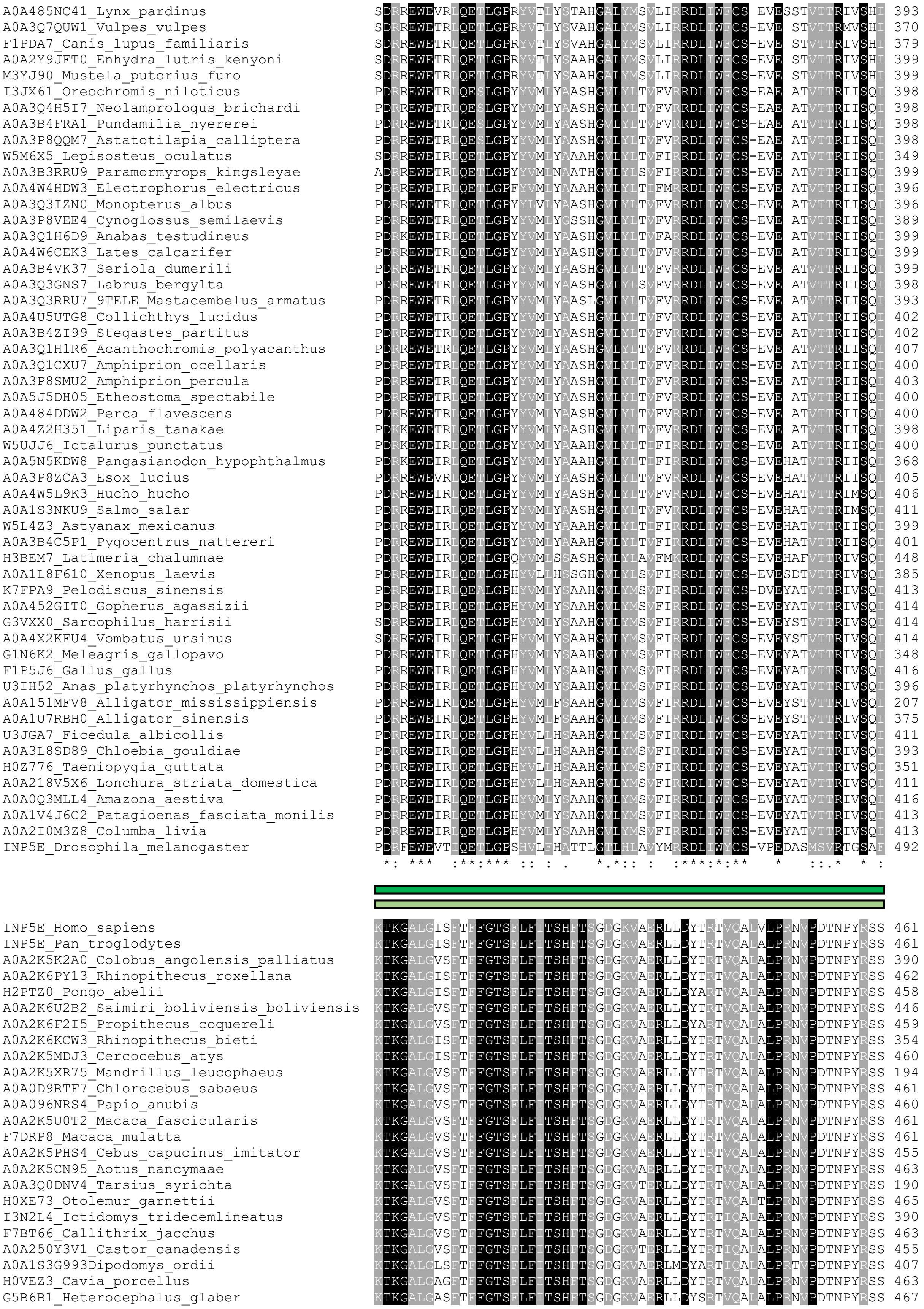
A0A485NC41_Lynx_pardinus SDRREWEVRLQETLGPRYVTLYSTAHGALYMSVLIRRDLIWFCS EVESSTVTTRIVSHI 393
A0A3Q7QUW1_Vulpes_vulpes SDRREWETRLQETLGPRYVTLYSVAHGALYMSVLIRRDLIWFCS-EVE STVTTRMVSHI 370
F1PDA7_Canis_lupus_familiaris SDRREWETRLQETLGPRYVTLYSVAHGALYMSVLIRRDLIWFCS-EVE STVTTRIVSHI 379
A0A2Y9JFT0_Enhydra_lutris_kenyoni SDRREWETRLQETLGPRYVTLYSAAHGALYMSVLIRRDLIWFCS-EVE STVTTRIVSHI 399
M3YJ90_Mustela_putorius_furo SDRREWETRLQETLGPRYVTLYSAAHGALYMSVLIRRDLIWFCS-EVE STVTTRIVSHI 399
I3JX61_Oreochromis_niloticus PDRREWETRLQESLGPYYVMLYAASHGVLYLTVFVRRDLIWFCS-EAE ATVTTRIISQI 398
A0A3Q4H5I7_Neolamprologus_brichardi PDRREWETRLQESLGPYYVMLYAASHGVLYLTVFVRRDLIWFCS-EAE ATVTTRIISQI 398
A0A3B4FRA1_Pundamilia_nyererei PDRREWETRLQESLGPYYVMLYAASHGVLYLTVFVRRDLIWFCS-EAE ATVTTRIISQI 398
A0A3P8QQM7_Astatotilapia_calliptera PDRREWETRLQESLGPYYVMLYAASHGVLYLTVFVRRDLIWFCS-EAE ATVTTRIISQI 398
W5M6X5_Lepisosteus_oculatus SDRREWEIRLQETLGPYYVMLYAAHGVLYLTVFIRRDLIWFCS-EVE ATVTTRIISQI 349
A0A3B3RRU9_Paramormyrops_kingsleyae ADRREWETRLQETLGPYYVMLNATHGVLYLSVFIRRDLIWFCS-EVE ATVTTRIISQI 399
A0A4W4HDW3_Electrophorus_electricus PDRREWEIRLQETLGPFYVMLYAAHGVLYLTIFMRRDLIWFCS-EVE ATVTTRIISQI 396
A0A3Q3IZN0_Monopterus_albus PDRREWETRLQETLGPYYLVLYAASHGVLYLTVFVRRDLIWYCS-EVE ATVTTRIISQI 396
A0A3P8VEE4_Cynoglossus_semilaevis PDRREWETRLQETLGPYYVMLYCSSHGVLYLTVFVRRDLIWFCS-EVE ATVTTRIISQI 389
A0A3Q1H6D9_Anabas_testudineus PDRKEWEIRLQETLGPYYVMLYAASHGVLYLTVFARRDLIWFCS-EVE ATVTTRIISQI 399
A0A4W6CEK3_Lates_calcarifer PDRREWETRLQETLGPYYVMLYAASHGVLYLTVFVRRDLIWFCS-EVE ATVTTRIISQI 399
A0A3B4VK37_Seriola_dumerili PDRREWETRLQETLGPYYVMLYAASHGVLYLTVFVRRDLIWFCS-EVE ATVTTRIISQI 399
A0A3Q3GNS7_Labrus_bergylta PDRREWETRLQETLGPYYVMLYAASHGVLYLSVFVRRDLIWFCS-EVE ATVTTRIISQI 398
A0A3Q3RRU7_9TELE_Mastacembelus_armatus PDRREWETRLQETLGPYYVMLYAASLGVLYLTVFVRRDLIWFCS-EVE ATVTTRIISQI 393
A0A4U5UTG8_Collichthys_lucidus PDRREWETRLQETLGPYYVMLYAASHGVLYLTVFVRRDLIWFCS-EVE ATVTTRIISQI 402
A0A3B4ZI99_Stegastes_partitus PDRREWETRLQETLGPYYVMLYAASHGVLYLTVFVRRDLIWFCS-EVE ATVTTRIISQI 402
A0A3Q1H1R6_Acanthochromis_polyacanthus PDRREWETRLQETLGPYYVMLYAASHGVLYLTVFVRRDLIWFCS-EVE ATVTTRIISQI 407
A0A3Q1CXU7_Amphiprion_ocellaris PDRREWETRLQETLGPYYVMLYAASHGVLYLTVFVRRDLIWFCS-EVE ATVTTRIISQI 400
A0A3P8SMU2_Amphiprion_percula PDRREWETRLQETLGPYYVMLYAASHGVLYLTVFVRRDLIWFCS-EVE ATVTTRIISQI 403
A0A5J5DH05_Etheostoma_spectabile PDRREWETRLQETLGPYYVMLYAASHGVLYLTVFVRRDLIWFCS-EVE ATVTTRIISQI 400
A0A484DDW2_Perca_flavescens PDRREWETRLQETLGPYYVMLYAASHGVLYLTVFVRRDLIWFCS-EVE ATVTTRIISQI 400
A0A4Z2H351_Liparis_tanakae PDKKEWETRLQETLGPYYVMLYAASHGVLYLTVFVRRDLIWFCS-EVE ATVTTRIISQI 398
W5UJJ6_Ictalurus_punctatus PDRKEWEIRLQETLGPYYVMLYAAHGVLYLTIFIRRDLIWFCS-EVE ATVTTRIISQI 400
A0A5N5KDW8_Pangasianodon_hypophthalmus PDRKEWEIRLQETLGPYYVMLYAAHGVLYLTIFIRRDLIWFCS-EVEHATVTTRIISQI 368
A0A3P8ZCA3_Esox_lucius PDRREWEVRLQETLGPYYVMLYAASHGVLYLTVFVRRDLIWFCS-EVEHATVTTRIISQI 405
A0A4W5L9K3_Hucho_hucho PDRREWEIRLQETLGPYYVMLYAASHGVLYLTVFVRRDLIWFCS-EVEHATVTTRIMSQI 406
A0A1S3NKU9_Salmo_salar PDRREWEIRLQETLGPYYVMLYAASHGVLYLTVFVRRDLIWFCS-EVEHATVTTRIMSQI 411
W5L4Z3_Astyanax_mexicanus PDRREWEIRLQETLGPYYVMLYAAHGVLYLTIFIRRDLIWYCS-EVEHATVTTRIISQI 399
A0A3B4C5P1_Pygocentrus_nattereri PDRREWEIRLQETLGPYYVMLYAAHGVLYLTVFIRRDLIWFCS-EVEHATVTTRIISQI 401
H3BEM7_Latimeria_chalumnae PDRREWEIRLQETLGPQYVMLSSASHGVLYLAVFMKRDLIWFCS-EVEHAFVTTRIVSQI 448
A0A1L8F610_Xenopus_laevis PDRREWEIRLQETLGPHYVLLHSSGHGVLYLSVFIRRDLIWFCS-EVESDTVTTRIVSQI 385
K7FPA9_Pelodiscus_sinensis PDRREWEIRLQEALGPHYVMLYSAAHGVLYMSVFIRRDLIWFCS-DVEYATVTTRIVSQI 413
A0A452GIT0_Gopherus_agassizii PDRREWEIRLQETLGPHYVMLYSAAHGVLYMSVFIRRDLIWFCS-EVEYATVTTRIVSQI 414
G3VXX0_Sarcophilus_harrisii SDRREWEIRLQETLGPHYVMLYSAAHGVLYMSVFIRRDLIWFCS-EVEYSTVTTRIVSQI 414
A0A4X2KFU4_Vombatus_ursinus SDRREWEIRLQETLGPHYVMLYSAAHGVLYMSVFIRRDLIWFCS-EVEYSTVTTRIVSQI 414
G1N6K2_Meleagris_gallopavo PDRREWEIRLQETLGPHYVMLYSAAHGVLYMSVFIRRDLIWFCS-EVEYATVTTRIVSQI 348
F1P5J6_Gallus_gallus PDRREWEIRLQETLGPHYVMLYSAAHGVLYMSVFIRRDLIWFCS-EVEYATVTTRIVSQI 416
U3IH52_Anas_platyrhynchos_platyrhynchos PDRREWEIRLQETLGPHYVMLYSAAHGVLYMSVFIRRDLIWFCS-EVEYATVTTRIVSQI 396
A0A151MFV8_Alligator_mississippiensis PDRREWEIRLQETLGPHYVMLFSAAHGVLYMSVFIRRDLIWFCS-EVEYSTVTTRIVSQI 207
A0A1U7RBH0_Alligator_sinensis PDRREWEIRLQETLGPHYVMLFSAAHGVLYMSVFIRRDLIWFCS-EVEYSTVTTRIVSQI 375
U3JGA7_Ficedula_albicollis PDRREWEIRLQETLGPHYVLLHSAAHGVLYMSVFIRRDLIWFCS-EVEYATVTTRIVSQI 411
A0A3L8SD89_Chloebia_gouldiae PDRREWEIRLQETLGPHYVLLHSAAHGVLYMSVFIRRDLIWFCS-EVEYATVTTRIVSQI 393
H0Z776_Taeniopygia_guttata PDRREWEIRLQETLGPHYVLLHSAAHGVLYMSVFIRRDLIWFCS-EVEYATVTTRIVSQI 351
A0A218V5X6_Lonchura_striata_domestica PDRREWEIRLQETLGPHYVLLHSAAHGVLYMSVFIRRDLIWFCS-EVEYATVTTRIVSQI 411
A0A0Q3MLL4_Amazona_aestiva PDRREWEIRLQETLGPHYVMLYSAAHGVLYMSVFIRRDLIWFCS-EVEYATVTTRIVSQI 416
A0A1V4J6C2_Patagioenas_fasciata_monilis PDRREWEIRLQETLGPHYVMLYSAAHGVLYMSVFIRRDLIWFCS-EVEYATVTTRIVSQI 413
A0A2I0M3Z8_Columba_livia PDRREWEIRLQETLGPHYVMLYSAAHGVLYMSVFIRRDLIWFCS-EVEYATVTTRIVSQI 413
INP5E_Drosophila_melanogaster PDRFEWEVTLQETLGPSHVLFHATTLGILHLAVYMRRDLIWYCS-VPEDASMSVRTGSAF 492
*: *** :**:*** :: : . *.*:: : :***:*:** * ::.* * :
INP5E_Homo_sapiens KTKGALGISFTFFGTSFLFITSHFTSGDGKVAERLLDYTRTVQALVLPRNVPDTNPYRSS 461
INP5E_Pan_troglodytes KTKGALGISFTFFGTSFLFITSHFTSGDGKVAERLLDYTRTVQALALPRNVPDTNPYRSS 461
A0A2K5K2A0_Colobus_angolensis_palliatus KTKGALGVSFTFFGTSFLFITSHFTSGDGKVAERLLDYTRTVQALALPRNVPDTNPYRSS 390
A0A2K6PY13_Rhinopithecus_roxellana KTKGALGISFTFFGTSFLFITSHFTSGDGKVAERLLDYTRTVQALALPRNVPDTNPYRSS 462
H2PTZ0_Pongo_abelii KTKGALGISFTFFGTSFLFITSHFTSGDGKVAERLLDYARTVQALALPRNVPDTNPYRSS 458
A0A2K6U2B2_Saimiri_boliviensis_boliviensis KTKGALGVSFTFFGTSFLFITSHFTSGDGKVAERLLDYTRTVQALALPRNVPDTNPYRSS 446
A0A2K6F2I5_Propithecus_coquereli KTKGALGVSFTFFGTSFLFITSHFTSGDGKVAERLLDYARTVQALALPRNVPDTNPYRSS 459
A0A2K6KCW3_Rhinopithecus_bieti KTKGALGISFTFFGTSFLFITSHFTSGDGKVAERLLDYTRTVQALALPRNVPDTNPYRSS 354
A0A2K5MDJ3_Cercocebus_atys KTKGALGISFTFFGTSFLFITSHFTSGDGKVAERLLDYTRTVQALALPRNVPDTNPYRSS 460
A0A2K5XR75_Mandrillus_leucophaeus KTKGALGVSFTFFGTSFLFITSHFTSGDGKVAERLLDYTRTVQALALPRNVPDTNPYRSS 194
A0A0D9RTF7_Chlorocebus_sabaeus KTKGALGVSFTFFGTSFLFITSHFTSGDGKVAERLLDYTRTVQALALPRNVPDTNPYRSS 461
A0A096NRS4_Papio_anubis KTKGALGVSFTFFGTSFLFITSHFTSGDGKVAERLLDYTRTVQALALPRNVPDTNPYRSS 460
A0A2K5U0T2_Macaca_fascicularis KTKGALGVSFTFFGTSFLFITSHFTSGDGKVAERLLDYTRTVQALALPRNVPDTNPYRSS 461
F7DRP8_Macaca_mulatta KTKGALGVSFTFFGTSFLFITSHFTSGDGKVAERLLDYTRTVQALALPRNVPDTNPYRSS 461
A0A2K5PHS4_Cebus_capucinus_imitator KTKGALGVSFTFFGTSFLFITSHFTSGDGKVAERLLDYTRTVQALALPRNVPDTNPYRSS 455
A0A2K5CN95_Aotus_nancymaae KTKGALGVSFTFFGTSFLFITSHFTSGDGKVAERLLDYTRTVQALALPRNVPDTNPYRSS 463
A0A3Q0DNV4_Tarsius_syrichta KTKGALGVSFIFFGTSFLFITSHFTSGDGKVTERLLDYTRTVQALALPRNVPDTNPYRSS 190
H0XE73_Otolemur_garnettii KTKGALGVSFTFFGTSFLFITSHFTSGDGKVAERLLDYTRTVQALTLPRNVPDTNPYRSS 465
I3N2L4_Ictidomys_tridecemlineatus KTKGALGVSFTFFGTSFLFITSHFTSGDGKVAERLLDYTRTIQALALPRNVPDTNPYRSS 390
F7BT66_Callithrix_jacchus KTKGALGVSFTFFGTSFLFITSHFTSGDGKVAERLLDYTRTVQALALPRNVPDTNPYRSS 463
A0A250Y3V1_Castor_canadensis KTKGALGVSFTFFGTSFLFITSHFTSGDGKVTERLLDYTRTIQALALPRNVPDTNPYRSS 455
A0A1S3G993Dipodomys_ordii KTKGALGLSFTFFGTSFLFITSHFTSGDGKVAERLMDYARTIQALALPRTVPDTNPYRSS 407
H0VEZ3_Cavia_porcellus KTKGALGAGFTFFGTSFLFITSHFTSGDGKVAERLLDYTRTVQALALPRNVPDTNPYRSS 463
G5B6B1_Heterocephalus_glaber KTKGALGASFTFFGTSFLFITSHFTSGDGKVAERLLDYSRTVQALALPRNVPDTNPYRSS 467

```
A0A2Y9E841_Trichechus_manatus_latirostris    KTKGALGVGFTFFGTSFLFITSHFTSGDGKVAERLLDYTRTIRALALPRSVPDTNPYRSS 442
L5KA82_Pteropus_alecto                       KTKGALGVSFTFFGTSFLFITSHFTSGDGKVGDRLLDYSKTVQGLALPKNVPDTCPYRSD 437
A0A5N4EAQ5_Camelus_dromedarius               KTKGALGVGFTFFGTSFLFITSHFTSGDGKVSERLVDYSKTIQGLALPRNVPDTSPYRSD 473
A0A3Q2HCU7_Equus_caballus                    KTKGALGVSFTFFGTSFLFITSHFTSGDGKVSERLVDYTKTIQGLALPKNVPDTSPYRSD 470
A0A1S2ZM74_Erinaceus_europaeus               KTKGALGVSFTFFGTSFLFITSHFTSGDGKVNERLLDYTKTVQGLTLPKNVPETSPYRSD 464
A0A3Q0DCZ7_Mesocricetus_auratus              KTKGALGVSFTFFGTSFLFITSHFTSGDGKVAERLLDYSRTIQALALPRNVPDTNPYRSS 456
G3I6Z4_Cricetulus_griseus                    KTKGALGVSFTFFGTSFLFITSHFTSGDGKVAERLLDYSRTIQALALPRNVPDTNPYRSS 456
INP5E_Rattus_norvegicus                      KTKGALGVSFTFFGTSFLFITSHFTSGDGKVAERLLDYNRTIQALALPRNVPDTNPYRSS 465
INP5E_Mus_musculus                           KTKGALGVSFTFFGTSFLFITSHFTSGDGKVAERLLDYSRTIQALALPRNVPDTNPYRSS 464
A0A2Y9NRK2_Delphinapterus_leucas             KTKGALGVSFTFFGTSLLFITSHFTSGDGKVGERLVDYSKTVQGLALPKNVPDTSPYRSD 444
A0A2Y9SXS0_Physeter_macrocephalus            KTKGALGVSFTFFGTSLLFITSHFTSGDGKVGERLADYSKTVQGLALPKNVPATSPYRSD 429
E1BAU6_Bos_taurus                            KTKGALGVSFTFFGTSLLFITSHFTSGDGKVGERLVDYSKTVQGLALPKNVPDTSPYRSD 473
A0A4W2BWU9_Bos_indicus_x_Bos_taurus          KTKGALGVSFTFFGTSLLFITSHFTSGDGKVGERLVDYSKTVQGLALPKNVPDTSPYRSD 473
L8IYW6_Bos_mutus                             KTKGALGVSFTFFGTSLLFITSHFTSGDGKVGERLVDYSKTVQGLALPKNVPDTSPYRSD 473
A0A452DQV4_Capra_hircus                      KTKGALGVSFTFFGTSLLFITSHFTSGDGKVGERLVDYSKTVQGLALPKNVPDTSPYRSD 473
A0A5J5MJZ6_Muntiacus_reevesi                 KTKGALGVSFTFFGTSLLFITSHFTSGDGKVGERLVDYSKTVQGLALPKNVPDTSPYRSD 473
A0A5N3WU40_Muntiacus_muntjak                 KTKGALGVSFTFFGTSLLFITSHFTSGDGKVGERLVDYSKTVQGLALPKNVPDTSPYRSD 473
A0A3Q7V225_Ursus_arctos_horribilis           KTKGALGVSFTFFGTSFLFITSHFTSGDGKVNERLLDYSRTVQGLALPKSVPDTSPYRSD 459
A0A452Q8J6_Ursus_americanus                  KTKGALGVSFTFFGTSFLFITSHFTSGDGKVNERLLDYSRTVQGLALPKSVPDTSPYRSD 459
A0A452VEA9_Ursus_maritimus                   KTKGALGVSFTFFGTSFLFITSHFTSGDGKVNERLLDYSRTVQGLALPKSVPDTSPYRSD 463
A0A2U3ZR12_Odobenus_rosmarus_divergens       KTKGALGVSFTFFGTSFLFITSHFTSGDGKVSERLLDYSRTVQGLALPKSVPDTSPFRSD 429
A0A3Q7N7C7_Callorhinus_ursinus               KTKGALGVSFTFFGTSFLFITSHFTSGDGKVSERLLDYSRTVQGLALPKSVPDTSPFRSD 429
A0A2Y9HBQ8_Neomonachus_schauinslandi         KTKGALGISFTFFGTSFLFITSHFTSGDGKVSERLLDYSRTVQGLALPRSVPDTSPFRSD 430
A0A485NC41_Lynx_pardinus                     KTKGALGVSFTFFGTSFLFITSHFTSGDGKVSERLLDYSRTVQGLALPRSVPDTSPYRSD 453
A0A3Q7QUW1_Vulpes_vulpes                     KTKGALGVSFTFFGTSFLFITSHFTSGDGKVSERLLDYSRTIQGLALPKSVPDTSPYRSD 430
F1PDA7_Canis_lupus_familiaris                KTKGALGVSFTFFGTSFLFITSHFTSGDGKVSERLLDYSRTIQGLALPKSVPDTSPYRSD 439
A0A2Y9JFT0_Enhydra_lutris_kenyoni            KTKGALGVSFTFFGTSLLFITSHFTSGDGKVSERLLDYSRTIQGLALPKSVPDTSPYRSD 459
M3YJ90_Mustela_putorius_furo                 KTKGALGVSFTFFGTSLLFITSHFTSGDGKVSERLLDYSRTVQGLALPKSVPDTSPYRSD 459
I3JX61_Oreochromis_niloticus                 KTKGAVGIAFTFFGTSFLFVTSHFTSGDAKVYERILDYNKIIEALALPKGLPDTNPYRST 458
A0A3Q4H5I7_Neolamprologus_brichardi          KTKGAVGIAFTFFGTSFLFVTSHFTSGDAKVYERILDYNKIVEALALPKGLPDTNPYRST 458
A0A3B4FRA1_Pundamilia_nyererei               KTKGAVGIAFTFFGTSFLFVTSHFTSGDAKVYERILDYNKIVEALALPKGLPDTNPYRST 458
A0A3P8QQM7_Astatotilapia_calliptera          KTKGAVGIAFTFFGTSFLFVTSHFTSGDAKVYERILDYNKIVEALALPKGLPDTNPYRST 458
W5M6X5_Lepisosteus_oculatus                  KTKGALGIGFTFFGTSFLFITSHFTSGDSKVYERILDYNKIIEALALPRDLPDTNPYRSS 409
A0A3B3RRU9_Paramormyrops_kingsleyae          KTKGAVGICLTFFGTSFLFITSHFTSGDTKVYERILDYNKIIEALALPRSLPDTNPYRST 459
A0A4W4HDW3_Electrophorus_electricus          KTKGAVGVGFTFFGTSFLFITSHFTSGDSKVYERILDYNKIIEALALPRCLPDTNPYRSV 456
A0A3Q3IZN0_Monopterus_albus                  KTKGAVGVAFMFFGTSFLFITSHFTSGDAKVYERILDYNKIIEALGLPRGLPDTNPYRST 456
A0A3P8VEE4_Cynoglossus_semilaevis            KTKGAVGVTFTFFGTSFLFITSHFTSGDAKVYERILDYNKIIEALALPKSLPDTNPYRST 449
A0A3Q1H6D9_Anabas_testudineus                KTKGAVGITFTFFGTSFLFITSHFTSGDAKVYERILDYNKIVEALALPKGLPDTNPYRST 459
A0A4W6CEK3_Lates_calcarifer                  KTKGAVGIAFTFFGTSFLFITSHFTSGDAKVYERILDYNKIVEALALPKGLPDTNPYRST 459
A0A3B4VK37_Seriola_dumerili                  KTKGAVGIAFTFFGTSFLFITSHFTSGDAKVYERILDYNKIVEALALPKGLPDTNPYRST 459
A0A3Q3GNS7_Labrus_bergylta                   KTKGAVGLAFTFFGTSFLFITSHFTSGDSKVYERILDYNKIVEALALPKGLPDTNPYRST 458
A0A3Q3RRU7_9TELE_Mastacembelus_armatus       KTKGAVGIAFTFFGTSFLFITSHFTSGDAKIYERILDYNKIVEALGLPKDLPDTNPYRST 453
A0A4U5UTG8_Collichthys_lucidus               KTKGAVGIAFTFFGTSFLFITSHFTSGDAKIYERILDYNKIVEALALPKGLPDTNPYRST 462
A0A3B4ZI99_Stegastes_partitus                KTKGAVGIAFTFFGTSFLFITSHFTSGDAKVYERILDYNKIVEALALPKGLPDTNPYRST 462
A0A3Q1H1R6_Acanthochromis_polyacanthus       KTKGAVGIAFTFFGTSFLFITSHFTSGDSKVYERILDYNKIVEALALPKGLPDTNPYRST 467
A0A3Q1CXU7_Amphiprion_ocellaris              KTKGAVGIAFTFFGTSFLFITSHFTSGDAKVYERILDYNKIVEALALPKGLPDTNPYRST 460
A0A3P8SMU2_Amphiprion_percula                KTKGAVGIAFTFFGTSFLFITSHFTSGDAKVYERILDYNKIVEALALPKGLPDTNPYRST 463
A0A5J5DH05_Etheostoma_spectabile             KTKGAVGIAFTFFGTSFLFITSHFTSGDAKVYERVLDYNKIVEALALPKGLPDTNPYRST 460
A0A484DDW2_Perca_flavescens                  KTKGAVGIAFTFFGTSFLFITSHFTSGDAKVYERVLDYNKIVEALALPKGLPDTNPYRST 460
A0A4Z2H351_Liparis_tanakae                   KTKGAVGIAFTFFGTSFLFITSHFTSGDAKVYERVLDYNKIIEALALPKGLPDTNPYRST 458
W5UJJ6_Ictalurus_punctatus                   KTKGAVGIGFTFFGTSFLFITSHFTSGDSKVYERILDYNKIIEALALPRCLPDTNPYRSV 460
A0A5N5KDW8_Pangasianodon_hypophthalmus       KTKGAVGIGFTFFGTSFLFITSHFTSGDSKVYERILDYNKIIEALALPRCLPDTNPYRSV 428
A0A3P8ZCA3_Esox_lucius                       KTKGAVGIGFTFFGTSFLFITSHFTSGDSKVYERILDYNKIIEAMALPKGLPDTNPYRSM 465
A0A4W5L9K3_Hucho_hucho                       KTKGAVGIGFTFFGTSFLFITSHFTSGDSRVYERILDYNKIVEALALPKVLPDTNPYRST 466
A0A1S3NKU9_Salmo_salar                       KTKGAVGIGFTFFGTSFLFITSHFTSGDSRVYERILDYNKIVEALALPKVLPDTNPYRST 471
W5L4Z3_Astyanax_mexicanus                    KTKGAVGIGFTFFGTSFLFITSHFTSGDSKVYERILDYNKIVEALALPRGLPDTNPYRSI 459
A0A3B4C5P1_Pygocentrus_nattereri             KTKGAVGIGFTFFGTSFLFITSHFTSGDSKVYERILDYNKIIEALALPRCLPDTNPYRST 461
H3BEM7_Latimeria_chalumnae                   KTKGAVGVCFTFFGTSFLFVTSHFTSGSTKVYERILDYKKIIEALALPRNLPDTNPYRSD 508
A0A1L8F610_Xenopus_laevis                    KTKGALAVSFTFFGTSFLFITSHFTSGDSKVADRILDYKKIIEGLQLPRNIPDTNPYRSN 445
K7FPA9_Pelodiscus_sinensis                   KTKGALGICFTFFGTSFLFITSHFTSGDGKVYERTLDYNKTIQALGLPKNVPDTNPYRSS 473
A0A452GIT0_Gopherus_agassizii                KTKGALGICFTFFGTSFLFITSHFTSGDGKVCERILDYNKTIQALALPKNVPDTNPYRSS 474
G3VXX0_Sarcophilus_harrisii                  KTKGSLGVSFTFFGTSFLFITSHFTSGDGKVSDRLLDYSKTIQTLTLPKNVPDTNPYQSS 474
A0A4X2KFU4_Vombatus_ursinus                  KTKGSLGVSFTFFGTSFLFITSHFTSGDGKVSDRLLDYSKTIQALTLPKNVPDTNPYHSS 474
G1N6K2_Meleagris_gallopavo                   KTKGALGICFTFFGTSFLFITSHFTSGDSKVNERKLDYNKTIQALTLPKVVPDTNPYRSS 408
F1P5J6_Gallus_gallus                         KTKGALGICFTFFGTSFLFITSHFTSGDSKVNERKLDYNKTIQALTLPKVVPDTNPYRSS 476
U3IH52_Anas_platyrhynchos_platyrhynchos      KTKGALGICFTFFGTSFLFITSHFTSGDSKVNERKLDYNKTIQALTLPKNVPDTNPYRSS 456
A0A151MFV8_Alligator_mississippiensis        KTKGALGISFTFFGTSFLFITSHFTSGDSKVYERILDYNKIIQALALPKNVPDTNPYRSS 267
A0A1U7RBH0_Alligator_sinensis                KTKGALGISFTFFGTSFLFITSHFTSGDSKVYERILDYNKIIQALALPKNVPDTNPYRSS 435
U3JGA7_Ficedula_albicollis                   KTKGALGISFTFFGTSFLFITSHFTSGDSKVNERKLDYNKTIQALTLPKNVPDTNPYRSS 471
A0A3L8SD89_Chloebia_gouldiae                 KTKGALGISFTFFGTSFLFITSHFTSGDSKVNERKLDYNKTIQALTLPKNVPDTNPYRSS 453
H0Z776_Taeniopygia_guttata                   KTKGALGISFTFFGTSFLFITSHFTSGDSKVNERKLDYNKTIQALTLPKNVPDTNPYRSS 411
A0A218V5X6_Lonchura_striata_domestica        KTKGALGISFTFFGTSFLFITSHFTSGDSKVNERKLDYNKTIQALTLPKNVPDTNPYRSS 471
A0A0Q3MLL4_Amazona_aestiva                   KTKGALGISFTFFGTSFLFITSHFTSGDSKVNERKLDYNKTIQSLTLPKNVPDTNPYRSS 476
A0A1V4J6C2_Patagioenas_fasciata_monilis      KTKGALGISFTFFGTSFLFITSHFTSGDSKVNERKLDYNKTIQALTLPKNVPDTNPYRSS 473
A0A2I0M3Z8_Columba_livia                     KTKGALGISFTFFGTSFLFITSHFTSGDSKVNERKLDYNKTIQALTLPKNVPDTNPYRSS 473
INP5E_Drosophila_melanogaster                RTKGAVAISFCLFGTSMLFVTSHLTAHQQKVKERVSDVKRIINALDLPRNLPNQ---RHK 549
                                             :***::.  : :****:**:***:*: . :: :*  *  : :. : **: :*     :

INP5E_Homo_sapiens                           AADVTTRFDEVFWFGDFNFRLSGGRTVVDALLCQGLV----VDVPALLQHDQLIREMRKG 517
```

```
INP5E_Pan_troglodytes                         AADVTTRFDEVFWFGDFNFRLSGGRTVVDALLCQGLV----VDVPALLQHDQLIREMRKG 517
A0A2K5K2A0_Colobus_angolensis_palliatus       AADVTTRFDEVFWFGDFNFRLSGGRTAVDTLLCQGLV----VDVPALLQHDQLIQEMRKG 446
A0A2K6PY13_Rhinopithecus_roxellana            AADVTTRFDEVFWFGDFNFRLSGGRTAVDTLLCQGLV----VDVPALLQHDQLIREMRKG 518
H2PTZ0_Pongo_abelii                           AADVTTRFDEVFWFGDFNFRLSGGRTVVDALLCQGLV----VDVPALLQHDQLIQEMQKG 514
A0A2K6U2B2_Saimiri_boliviensis_boliviensis    AADVTTRFDQVFWFGDFNFRLSGGRAAVDALLGQGLA----VDVPALLEHDQLIREMRRG 502
A0A2K6F2I5_Propithecus_coquereli              AADVTTRFDEVFWFGDFNFRLSGGRATVDALLLQGPE----ADVPALLQHDQLTREMRKG 515
A0A2K6KCW3_Rhinopithecus_bieti                AADVTTRFDEVFWFGDFNFRLSGGRTAVDTLLCQGLV----VDVPALLQHDQLIREMRKG 410
A0A2K5MDJ3_Cercocebus_atys                    AADVTTRFDEVFWFGDFNFRLSGGRTAVDTLLCQGLV----VDVPALLQHDQLIREMRKG 516
A0A2K5XR75_Mandrillus_leucophaeus             AVDVTTRFDEVFWFGDFNFRLSGGRTAVDTLLCQGLV----VDVPALLQHDQLIREMRKG 250
A0A0D9RTF7_Chlorocebus_sabaeus                AADVTTRFDEVFWFGDFNFRLSGGRTAVDTLLCQGLV----VDVPALLQHDQLIREMRKG 517
A0A096NRS4_Papio_anubis                       AADVTTRFDEVFWFGDFNFRLSGGRTAVDTLLCQGLV----VDVPALLQHDQLIREMRKG 516
A0A2K5U0T2_Macaca_fascicularis                AADVTTRFDEVFWFGDFNFRLSGGRTAVDTLLCQGLV----VDVPALLQHDQLIREMRKG 517
F7DRP8_Macaca_mulatta                         AADVTTRFDEVFWFGDFNFRLSGGRTAVDTLLCQGLV----VDVPALLQHDQLIREMRKG 517
A0A2K5PHS4_Cebus_capucinus_imitator           AADVTTRFDQVFWFGDFNFRLSGGRAAVDALLGRGLA----VDVPALLEHDQLIREMRRG 511
A0A2K5CN95_Aotus_nancymaae                    AADVTTRFDQVFWFGDFNFRLSGGRAAVDALLGKGLA----VDVPALLEHDQLIREMRRG 519
A0A3Q0DNV4_Tarsius_syrichta                   PADVTTRFDEVFWFGDFNFRLSGGRAAVDALLHQGLA----ADVPALLQRDQLLREMQKG 246
H0XE73_Otolemur_garnettii                     AADVTTRFDEVFWFGDFNFRLSGGRAAVDTLLLQGAE----TDVPTLLQHDQLTREMRKG 521
I3N2L4_Ictidomys_tridecemlineatus             AADVTTRFDEVFWFGDFNFRLSGGRMAVEAILKQGLD----VDVPALLQHDQLIQEMKKG 446
F7BT66_Callithrix_jacchus                     AADVTTRFDQVFWFGDFNFRLSGGRVAVDALLGQGLA----VDVPALLEHDQLIQEMRKG 519
A0A250Y3V1_Castor_canadensis                  AADVTTRFDEVFWFGDFNFRLSGGRVAVEAILKKDVE----VDVQALLQHDQLTREMKKG 511
A0A1S3G993Dipodomys_ordii                     AADVTTRFDEVFWFGDFNFRLSGGRRAVEAILKQDLE----VDVPALLRHDQLTREMKKG 463
H0VEZ3_Cavia_porcellus                        TADVTTRFDEVFWFGDFNFRLGGGRAAVEAALRQERE----VAVRALLQQDQLTREMKQG 519
G5B6B1_Heterocephalus_glaber                  AADVTTRFDAVFWFGDFNFRLGGGRAAVEGALRQERE----AAVRVLLQQDQLTREMKRG 523
A0A2Y9E841_Trichechus_manatus_latirostris     PVDVTTRFDEVFWFGDFNFRLSAGRTAVEALLRQELE----TDMSALLQHDQLTREMRRG 498
L5KA82_Pteropus_alecto                        PADVTTRFDGVFWFGDFNFRLGAGRVAVEAILRQDPG----ASVPALLQLDQLTREMKKG 493
A0A5N4EAQ5_Camelus_dromedarius                AADVTTRFDGVFWFGDFNFRLSGGRAAVEAILKQDPG----ADVQALLQRDQLTREMKRG 529
A0A3Q2HCU7_Equus_caballus                     SADVTTRFDGVFWFGDFNFRLSGGRMAVETILKQDLG----VNVPALLQHDQLTREMKKG 526
A0A1S2ZM74_Erinaceus_europaeus                SADVTSRFDGVFWFGDFNFRLSSGRAAVEAILKQDVG----ARVPALLQHDQLTREMRKG 520
A0A3Q0DCZ7_Mesocricetus_auratus               AGDVTTRFDEVFWFGDFNFRLSGGRAAVEAFLKQDPE----VDVLALLQHDQLTQEMKKG 512
G3I6Z4_Cricetulus_griseus                     AGDVTTRFDEVFWFGDFNFRLSGGRVAVEAFLKQDPE----VDVLALLQHDQLTREMKKG 512
INP5E_Rattus_norvegicus                       AGDVTTRFDEVFWFGDFNFRLSGGRVAVEAFLKQDPE----VDVLALLQHDQLTREMKKG 521
INP5E_Mus_musculus                            AGDVTTRFDEVFWFGDFNFRLSGGRVAVEAFLKQKPE----VDVLALLQHDQLTREMKKG 520
A0A2Y9NRK2_Delphinapterus_leucas              AADVTSRFDGVFWFGDFNFRLSGGRAAVEAILKQDPG----ASVQELLQHDQLTREMKRG 500
A0A2Y9SXS0_Physeter_macrocephalus             AADVTTRFDGVFWFGDFNFRLSGGRAAVEAILKQDPG----VSVQALLQHDQLTREMKRG 485
E1BAU6_Bos_taurus                             AADVTTRFDEVFWFGDFNFRLSGGRAAVEAILKQDLS----SSVQALLQHDQLTREMQRG 529
A0A4W2BWU9_Bos_indicus_x_Bos_taurus           AADVTTRFDEVFWFGDFNFRLSGGRAAVEAILKQDLS----SSVQALLQHDQLTREMQRG 529
L8IYW6_Bos_mutus                              AADVTTRFDEVFWFGDFNFRLSGGRAAVEAILKQDLS----SSVQALLQHDQLTREMQRG 529
A0A452DQV4_Capra_hircus                       AADVTTRFDEVFWFGDFNFRLSGGRAAVEAILKQDLG----SSVQALLQHDQLTLEMQRG 529
A0A5J5MJZ6_Muntiacus_reevesi                  AADVTTRFDEVFWFGDFNFRLSGGRAAVEAILKQDLG----SSVQALLERDQLTREMQRG 529
A0A5N3WU40_Muntiacus_muntjak                  AADVTTRFDEVFWFGDFNFRLSGGRAAVEAILKQDLG----SSVQALLERDQLTREMQRG 529
A0A3Q7V225_Ursus_arctos_horribilis            AADVTTRFDGVFWFGDFNFRLSGGRVAVEAILKQDLG----EKVSALLQHDQLTQEMRKG 515
A0A452Q8J6_Ursus_americanus                   AADVTTRFDGVFWFGDFNFRLSGGRVAVEAILKQDLG----EKVSALLQHDQLTQEMRKG 515
A0A452VEA9_Ursus_maritimus                    AADVTTRFDGVFWFGDFNFRLS--RVAVEAILKQDLG----EKVSALLQHDQLTQEMRKG 517
A0A2U3ZR12_Odobenus_rosmarus_divergens        AADVTTRFDGVFWFGDFNFRLSGGRVAVEAILKQDLG----EKVSALLQHDQLTQEMKKG 485
A0A3Q7N7C7_Callorhinus_ursinus                AADVTTRFDGVFWFGDFNFRLSGGRVAVEAILKQDLG----EKVSALLQHDQLTQEMKKG 485
A0A2Y9HBQ8_Neomonachus_schauinslandi          AADVTTRFDGVFWFGDFNFRLSGGRVAVEAILKQDLG----EKVSALLQHDQLTQEMKKG 486
A0A485NC41_Lynx_pardinus                      AADVTTRFDGVFWFGDFNFRLNGGRVAVEAILKQDLG----EKVSALLQHDQLTQEMKKG 509
A0A3Q7QUW1_Vulpes_vulpes                      AADVTTRFDGVFWFGDFNFRLSGGRVAVEAILKQDLV----EKVSALLQHDQLTQEMKKG 486
F1PDA7_Canis_lupus_familiaris                 AADVTTRFDGVFWFGDFNFRLSGGRVAVEAILKQDLV----EKVSTLLQHDQLTQEMKKG 495
A0A2Y9JFT0_Enhydra_lutris_kenyoni             AADVTTRFDGVFWFGDFNFRLSGGRVAVEAILKQDLG----EKVSALLQHDQLTQEMKKG 515
M3YJ90_Mustela_putorius_furo                  AADVTTRFDGVFWFGDFNFRLSGGRMAVEAILKQDLG----EKVSALLQHDQLTQEMKKG 515
I3JX61_Oreochromis_niloticus                  PSDVTTRFDQVFWFGDFNFRLDKDRSEVEAIMSSTVG----GDMSPLLEHDQLSKEMKEG 514
A0A3Q4H5I7_Neolamprologus_brichardi           PSDVTTRFDQVFWFGDFNFRLDKDRSEVEAVISSTVG----GEMSPLLEHDQLSKEMKEG 514
A0A3B4FRA1_Pundamilia_nyererei                PSDVTTRFDQVFWFGDFNFRLDKDRSEVEAIISSTVG----GDMSPLLEHDQLSKEMKEG 514
A0A3P8QQM7_Astatotilapia_calliptera           PSDVTTRFDQVFWFGDFNFRLDKDRSEVEAIISSTVG----GDMSPLLEHDQLSKEMKEG 514
W5M6X5_Lepisosteus_oculatus                   SSDVTTRFDEVFWFGDFNFRLNEDRTGVESILKQDLG----SDMSRLLQYDQLSKEMKDG 465
A0A3B3RRU9_Paramormyrops_kingsleyae           SSDVTTRFDEVFWFGDFNFRLEKDREGVEAILR-ELE----SDLAPLLQYDQLAKEMRDG 514
A0A4W4HDW3_Electrophorus_electricus           ASDVTTRFDEVFWFGDFNFRLSKDRHGVDAILNEHTG----TNMGSLLQHDQLHKEMKDG 512
A0A3Q3IZN0_Monopterus_albus                   SSDVTTRFDQVFWFGDFNFRLSEERANVEAIMSHTSG----GDMGLLLEHDQLSKEMKDG 512
A0A3P8VEE4_Cynoglossus_semilaevis             PSDVTTRFDQVFWFGDFNFRLSKERSDVDAMMKRTEG----GDMSVLLEHDQLSIELKDG 505
A0A3Q1H6D9_Anabas_testudineus                 SSDVTTRFDQVFWFGDFNFRLSKDRVEVENVMNRTVG----DDMSPLVEHDQLSKEMKEG 515
A0A4W6CEK3_Lates_calcarifer                   PSDVTTRFDQVFWFGDFNFRLSKDRVDVESIMNRTVD----GDMGPLLEHDQLFKEMKDG 515
A0A3B4VK37_Seriola_dumerili                   PSDVTTRFDQVFWFGDFNFRLSKDRVDVETLMNHTGA----GNMDTLLEHDQLSKEMKDG 515
A0A3Q3GNS7_Labrus_bergylta                    PSDVTTRFDQVFWFGDFNFRISKDRVDVESMINRTVG----ENMGPLLEHDQLSKEIKEG 514
A0A3Q3RRU7_9TELE_Mastacembelus_armatus        PSDVTTRFDQVFWFGDFNFRLSKDRVDVEAIMNRTVE----GDMGPLLEHDQLSKEMNDG 509
A0A4U5UTG8_Collichthys_lucidus                PSDVTTRFDQVFWFGDFNFRLSKDRVDVETIMNRTVA----GDMGPLLAHDQLSKEMKDG 518
A0A3B4ZI99_Stegastes_partitus                 PSDVTTRFDQVFWFGDFNFRLSKDRTDVETIMNRTAG----GEMGPLLEHDQLSKEMKDG 518
A0A3Q1H1R6_Acanthochromis_polyacanthus        PSDVTTRFDQVFWFGDFNFRLSKDRADVEMMMSQTGD----GDKSLLLEHDQLSKEMKDG 523
A0A3Q1CXU7_Amphiprion_ocellaris               PSDVTTRFDQVFWFGDFNFRLSKDRADVEMMMNRTGD----GDKSLLLEHDQLSKEMKDG 516
A0A3P8SMU2_Amphiprion_percula                 PSDVTTRFDQVFWFGDFNFRLSKDRADVEMMMNRTGD----GDKSLLLEHDQLSKEMKDG 519
A0A5J5DH05_Etheostoma_spectabile              PSDVTTRFDQVFWFGDFNFRLSKDRVDVETIINRTVG----GNMAPLLEHDQLSKEMKDG 516
A0A484DDW2_Perca_flavescens                   PSDVTTRFDQVFWFGDFNFRLSKDRVDVETIINRTVG----GDMGPLLEHDQLSKEMKDG 516
A0A4Z2H351_Liparis_tanakae                    ASDVTTRFDQVFWFGDFNFRLCKDRVDVEAIMSRMVG----AEMGPLLEHDQLSKEMKEG 514
W5UJJ6_Ictalurus_punctatus                    ASDVTTRFDEVFWFGDFNFRLSQDRGRVEDILNQLTV----KNMDALLQHDQLCKEMKDG 516
A0A5N5KDW8_Pangasianodon_hypophthalmus        ASDVTTRFDEVFWFGDFNFRLSQDRERVEDILNQLTG----KNMDALLQHDQLCKEMKDG 484
A0A3P8ZCA3_Esox_lucius                        PSDVTTRFDEVFWFGDFNFRLSKDRVGIDALLNQNHG----VDMGLLLHHDQLSKEMKDG 521
A0A4W5L9K3_Hucho_hucho                        SSDVTTRFDEVFWFGDFNFRLSEDRVGVEALLNQNQG----VDMGPLLHHDQLSKEMKDG 522
A0A1S3NKU9_Salmo_salar                        SSDVTTRFDEVFWFGDFNFRLSKDRVGVEALLNQNQG----VDMGPLLHHDQLSKEMKDG 527
W5L4Z3_Astyanax_mexicanus                     ASDVTTRFDEVFWFGDFNFRLSKDRGGVEAILNQHIG----KDMGPLLQHDQLCKEIKEG 515
A0A3B4C5P1_Pygocentrus_nattereri              ASDVTTRFDEVFWFGDFNFRLSKDRGGIEAILNQHIR----EDMGPLLQHDQLCKEMKDG 517
H3BEM7_Latimeria_chalumnae                    HLDVTTRFDEVLWFGDLNFRLANSRMEIDLLLKQNLK----NNMSSLLQYDQLLKEMQDG 564
```

```
A0A1L8F610_Xenopus_laevis                   SADVTSRFDEVFWFGDFNFRLNKERSIVNSILQSKLE----KDMSRLVQYDQLTKEMNGE 501
K7FPA9_Pelodiscus_sinensis                  PSDVTTRFDEVFWFGDFNFRLNKDRESVESILSQHLD----TDMSKLLQYDQLIKEMNDE 529
A0A452GIT0_Gopherus_agassizii               PSDVTTRFDEVFWFGDFNFRLNKDRESVDSILNQHLE----KDMSKLLQYDQLIKEMNDG 530
G3VXX0_Sarcophilus_harrisii                 SLDVTTRFDEVFWFGDFNFRLNGGRMAVESILKQNLE----MNMSELLQHDQLIKEMKKG 530
A0A4X2KFU4_Vombatus_ursinus                 SLDVTSRFDEVFWFGDFNFRLNGGRMAVESILKQNLE----MNMSELLQHDQLIKEMKKG 530
G1N6K2_Meleagris_gallopavo                  SGDVTTRFDEVFWFGDFNFRLNKDRETVDSILNQNPE----TGVSKLLAYDQLTSEMGRG 464
F1P5J6_Gallus_gallus                        SGDVTTRFDEVFWFGDFNFRLNKDRETVDSILNQNPE----TGVSKLLAYDQLTSEMGRG 532
U3IH52_Anas_platyrhynchos_platyrhynchos     SSDVTTRFDEVFWFGDFNFRLNKDRETVDSILNQNPE----TGVSKLLAYDQLTSEMSRG 512
A0A151MFV8_Alligator_mississippiensis       PSDVTTRFDEVFWFGDFNFRLNQDREAVNLILNQNLE----MDMSKLLQYDQLLKEMKNG 323
A0A1U7RBH0_Alligator_sinensis               PSDVTTRFDEVFWFGDFNFRLNQDREAVNLILNQNLE----MDMSKLLQYDQLLKEMKNG 491
U3JGA7_Ficedula_albicollis                  SSDVTTRFDEVFWFGDFNFRLNKDRETVDSILNQHPD----TDVSKLLVYDQLTSEMSRG 527
A0A3L8SD89_Chloebia_gouldiae                SSDVTTRFDEVFWFGDFNFRLNKDRETVDSILNQNPD----TDVSKLLVYDQLTSEMSRG 509
H0Z776_Taeniopygia_guttata                  SSDVTTRFDEVFWFGDFNFRLNKDRETVDSILNQNPD----TDVSKLLAYDQLTSEMSRG 467
A0A218V5X6_Lonchura_striata_domestica       SSDVTTRFDEVFWFGDFNFRLNKDRETVDSILNQNPD----TDVSKLLVYDQLTSEMSRG 527
A0A0Q3MLL4_Amazona_aestiva                  SSDVTTRFDEVFWFGDFNFRLNKDRETVDSILNQNPE----TDVSRLLAYDQLTSEMSRG 532
A0A1V4J6C2_Patagioenas_fasciata_monilis     SSDVTTRFDEVFWFGDFNFRLNKDRETVDSILNQNPE----TDVSKLLAYDQLISEMSRG 529
A0A2I0M3Z8_Columba_livia                    SSDVTTRFDDVFWFGDFNFRLNKDRETVDSILNQNPE----TDVSKLLAYDQLISEMSRG 529
INP5E_Drosophila_melanogaster               NKDVTQNFDNVFWCGDLNFRLGEPREKLLEWIQNTKFPLPSHLPHGYMHTDQLTSVLADG 609
                                              *** .** *:* **:***:   *  :   :               :  ***   :

INP5E_Homo_sapiens                          SIFKGFQEPDIHFLPSYKFDIGKDTYDSTSKQRTPSYTDRVLYRSRHKGD---------- 567
INP5E_Pan_troglodytes                       SIFKGFQEPDIHFLPSYKFDIGKDTYDSTSKQRTPSYTDRVLYRSRHKGD---------- 567
A0A2K5K2A0_Colobus_angolensis_palliatus     SIFKGFQEPDIHFLPSYKFDIGKDTYDSTSKQRTPSYTDRVLYRSRHKGD---------- 496
A0A2K6PY13_Rhinopithecus_roxellana          SIFKGFQEPDIHFLPSYKFDIGKDTYDSTSKQRTPSYTDRVLYRSRHKGD---------- 568
H2PTZ0_Pongo_abelii                         SIFKGFQEPDIHFLPSYKFDIEKDTYDSTSKQRTPSYTDRVLYRSRHKGD---------- 564
A0A2K6U2B2_Saimiri_boliviensis_boliviensis  SIFKGFQEPDIHFLPSYKFDIGKDTYDSTSKQRTPSYTDRVLYRSRHKGD---------- 552
A0A2K6F2I5_Propithecus_coquereli            SVFKGFQEPDIHFLPSYKFDIGKDTYDSTSKQRTPSYTDRVLYRSRHKGD---------- 565
A0A2K6KCW3_Rhinopithecus_bieti              SIFKGFQEPDIHFLPSYKFDIGKDTYDSTSKQRTPSYTDRVLYRSRHKGD---------- 460
A0A2K5MDJ3_Cercocebus_atys                  SIFKGFQEPDIHFLPSYKFDIGKDTYDSTSKQRTPSYTDRVLYRSRHKGD---------- 566
A0A2K5XR75_Mandrillus_leucophaeus           SIFKGFQEPDIHFLPSYKFDIGKDTYDSTSKQRTPSYTDRVLYRSRHKGD---------- 300
A0A0D9RTF7_Chlorocebus_sabaeus              SIFKGFQEPDIHFLPSYKFDIGKDTYDSTSKQRTPSYTDRVLYRSRHKGD---------- 567
A0A096NRS4_Papio_anubis                     SIFKGFQEPDIHFLPSYKFDIGKDTYDSTSKQRTPSYTDRVLYRSRHKGD---------- 566
A0A2K5U0T2_Macaca_fascicularis              SIFKGFQEPDIHFLPSYKFDIGKDTYDSTSKQRTPSYTDRVLYRSRHKGD---------- 567
F7DRP8_Macaca_mulatta                       SIFKGFQEPDIHFLPSYKFDIGKDTYDSTSKQRTPSYTDRVLYRSRHKGD---------- 567
A0A2K5PHS4_Cebus_capucinus_imitator         SIFKGFQEPDIHFLPSYKFDIGKDTYDSTSKQRTPSYTDRVLYRSRHKGD---------- 561
A0A2K5CN95_Aotus_nancymaae                  SIFKGFQEPDIHFLPSYKFDIGKDTYDSTSKQRTPSYTDRILYRSRHKGD---------- 569
A0A3Q0DNV4_Tarsius_syrichta                 SVFKGFQEADIHFLPSYKFDIGKDTYDSTSKQRTPSYTDRVLYRSRQKGD---------- 296
H0XE73_Otolemur_garnettii                   SIFKGFQEPDIHFLPSYKFDIGKDTYDSTSKQRTPSYTDRVLYRSRHKGD---------- 571
I3N2L4_Ictidomys_tridecemlineatus           SIFKGFQEPDIHFLPSYKFDIGKDTYDSTSKQRTPSYTDRVLYRSRHKGD---------- 496
F7BT66_Callithrix_jacchus                   EGCGVAAAGDEEGVRAAAGDEGSGLVGAAVVEGTPSYTDRVLYRSRHKGD---------- 569
A0A250Y3V1_Castor_canadensis                SIFKGFQEPDIHFLPSYKFDIGKDTYDSTSKQRTPSYTDRVLYKSRQKGD---------- 561
A0A1S3G993Dipodomys_ordii                   SIFKGFQEPDIHFLPSYKFDIGKDTYDSTSKQRTPSYTDRVLYRSRHKGD---------- 513
H0VEZ3_Cavia_porcellus                      SIFRGFQEPDIVFLPSYKFDLGKDTYDSTSKQRTPSYTDRVLYRSRHKGD---------- 569
G5B6B1_Heterocephalus_glaber                SVFRGFQEPDIVFLPSYKFDLGKDTYDSTSKQRTPSYTDRVLYRSRHKGD---------- 573
A0A2Y9E841_Trichechus_manatus_latirostris   AIFKGFQEPDIHFLPSYKFDIGKDTYDSTSKQRTPSYTDRVMYRSRHKDD---------- 548
L5KA82_Pteropus_alecto                      SVFRGFQEPDIHFLPSYKFDVGKDVYDTTSKQRTPSYTDRVVYRSRHKGD---------- 543
A0A5N4EAQ5_Camelus_dromedarius              SIFKGFQEPDIHFLPSYKFDIGKDSYDTTSKQRTPSYTDRVMYRSRHKDD---------- 579
A0A3Q2HCU7_Equus_caballus                   SIFKGFQEPDIHFLPSYKFDIGKDSYDTSSKQRTPSYTDRVMYRSRHKDD---------- 576
A0A1S2ZM74_Erinaceus_europaeus              SIFKSFQEPDIHFLPSYKFDIGKDSYDTTSKQRTPSYTDRVLYRSRHKDD---------- 570
A0A3Q0DCZ7_Mesocricetus_auratus             SIFRGFEEAEIHFLPSYKFDIGKDTYDSTSKQRTPSYTDRVLYKSRHKGD---------- 562
G3I6Z4_Cricetulus_griseus                   SIFRGFEEAEIHFLPSYKFDIGKDTYDSTSKQRTPSYTDRVLYKSRHKGD---------- 562
INP5E_Rattus_norvegicus                     SIFKGFEEAEIHFLPSYKFDIGKDTYDSTSKQRTPSYTDRVLYKSRHKGD---------- 571
INP5E_Mus_musculus                          SIFRGFEEAEIHFLPSYKFDIGKDTYDSTSKQRTPSYTDRVLYKSRHKGD---------- 570
A0A2Y9NRK2_Delphinapterus_leucas            SIFKGFQEPDIHFLPSYKFDVGKDSYDTTSKQRTPSYTDRVMYRSRHRDD---------- 550
A0A2Y9SXS0_Physeter_macrocephalus           SIFKGFQEPDIHFLPSYKFDVGKDSYDTTSKQRTPSYTDRVMYRSRHRDD---------- 535
E1BAU6_Bos_taurus                           SIFKGFQEPDIHFLPSYKFDVGKDSYDTTSKQRTPSYTDRILYRSRHQGD---------- 579
A0A4W2BWU9_Bos_indicus_x_Bos_taurus         SIFKGFQEPDIHFLPSYKFDVGKDSYDTTSKQRTPSYTDRILYRSRHQGD---------- 579
L8IYW6_Bos_mutus                            SIFKGFQEPDIHFLPSYKFDVGKDSYDTTSKQRTPSYTDRILYRSRHQGD---------- 579
A0A452DQV4_Capra_hircus                     SIFKGFQEPDIHFLPSYKFDVGKDSYDTTSKQRTPSYTDRILYRSRHKGD---------- 579
A0A5J5MJZ6_Muntiacus_reevesi                SIFKGFQEPDIHFLPSYKFDVGKDSYDTTSKQRTPSYTDRVLFRSRHKDD---------- 579
A0A5N3WU40_Muntiacus_muntjak                SIFKGFQEPDIHFLPSYKFDVGKDSYDTTSKQRTPSYTDRILFRSRHKDD---------- 579
A0A3Q7V225_Ursus_arctos_horribilis          SIFKGFQEPDIHFLPSYKFDVGKDSYDTTSKQRTPSYTDRVMYRSRHKGD---------- 565
A0A452Q8J6_Ursus_americanus                 SIFKGFQEPDIHFLPSYKFDVGKDSYDTTSKQRTPSYTDRVMYRSRHKGD---------- 565
A0A452VEA9_Ursus_maritimus                  SIFKGFQEPDIHFLPSYKFDVGKDSHATTSKQRTPSYTDRVMYRSRHKGD---------- 567
A0A2U3ZR12_Odobenus_rosmarus_divergens      SIFKGFQEPDIHFLPSYKFDIGKDSYDTTSKQRTPSYTDRVMYRSRHKGD---------- 535
A0A3Q7N7C7_Callorhinus_ursinus              SIFKGFQEPDIHFLPSYKFDIGKDSYDTTSKQRTPSYTDRVMYRSRHKGD---------- 535
A0A2Y9HBQ8_Neomonachus_schauinslandi        SVFRGFQEPDIHFLPSYKFDIGKDSYDTTSKQRTPSYTDRVMYRSRHKGD---------- 536
A0A485NC41_Lynx_pardinus                    SIFKGFQEPDIHFLPSYKFDIGKDSYDTTSKQRTPSYTDRVVYRSRHKGD---------- 559
A0A3Q7QUW1_Vulpes_vulpes                    SIFKGFQEPDIHFLPSYKFDIGKDSYDTTSKQRTPSYTDRVMYRSRHKGD---------- 536
F1PDA7_Canis_lupus_familiaris               SIFKGFQEPDIHFLPSYKFDIGKDSYDTTSKQRTPSYTDRVMYRSRHKGD---------- 545
A0A2Y9JFT0_Enhydra_lutris_kenyoni           SIFKGFQEPDIHFLPSYKFDIGKDSYDTTSKQRTPSYTDRVMYKSRHKGD---------- 565
M3YJ90_Mustela_putorius_furo                SIFKGFQEPDIHFLPSYKFDIGKDSYDTTSKQRTPSYTDRVMYKSRHKGD---------- 565
I3JX61_Oreochromis_niloticus                SIFKGFQEASIHFLPTYKFDIGCDVYDTTSKQRTPSYTDRILFRSRQAND---------- 564
A0A3Q4H5I7_Neolamprologus_brichardi         SIFKGFQEASIHFLPTYKFDIGRDVYDTTSKQRTPSYTDRILFRSRQAND---------- 564
A0A3B4FRA1_Pundamilia_nyererei              SIFKGFQEASIHFLPTYKFDIGRDVYDTTSKQRTPSYTDRILFRSRQAND---------- 564
A0A3P8QQM7_Astatotilapia_calliptera         SIFKGFQEASIHFLPTYKFDIGRDVYDTTSKQRTPSYTDRILFRSRQAND---------- 564
W5M6X5_Lepisosteus_oculatus                 SIFKGFEEAPIQFLPTYKFDIGCDTYDSTAKQRTPSYTDRVLYRSRQKDD---------- 515
A0A3B3RRU9_Paramormyrops_kingsleyae         SIFKGFQEAPISFLPTYKFDVGCDVYDTTSKKRIPSYTDRVLFRSRQKDD---------- 564
A0A4W4HDW3_Electrophorus_electricus         SIFKGFQEAPIHFPPTYKFDIGCDVYDTTSKQRTPSYTDRILFRNRQADD---------- 562
```

```
A0A3Q3IZN0_Monopterus_albus                  SVFKGFQEAPIHFLPTYKFDINCDIYDTTSKQRTPSYTDRILFRNRQADD---------- 562
A0A3P8VEE4_Cynoglossus_semilaevis            SIFKGFQEASIHFFPTYKFDIGCDIYDTTSKQRTPSYTDRILFRNRQADD---------- 555
A0A3Q1H6D9_Anabas_testudineus                SIFKGFQEAPIRFFPTYKFDIGCDIYDTTSKQRTPSYTDRILFRSRQADD---------- 565
A0A4W6CEK3_Lates_calcarifer                  SIFKGFQEATIHFLPTYKFDIGCDIYDTTSKQRTPSYTDRILFRNRQAED---------- 565
A0A3B4VK37_Seriola_dumerili                  SIFKGFQEATIHFLPTYKFDIGCDIYDTTSKQRTPSYTDRILFRNRQADD---------- 565
A0A3Q3GNS7_Labrus_bergylta                   SIFKGFQEAPIHFRPTYKFDVGCDIYDTTSKQRTPSYTDRILFRNRQADD---------- 564
A0A3Q3RRU7_9TELE_Mastacembelus_armatus       SIFKGFQEAPIHFFPTYKFDIGCDIYDTTSKQRTPSYTDRILFRNRQAGD---------- 559
A0A4U5UTG8_Collichthys_lucidus               SIFKGFQEAPIHFFPTYKFDIGCDIYDTTSKQRTPSYTDRILFRNRQAND---------- 568
A0A3B4ZI99_Stegastes_partitus                SIFKGFQEAPIHFYPTYKFDIGCDIYDTTSKQRTPSYTDRILFRNRQADD---------- 568
A0A3Q1H1R6_Acanthochromis_polyacanthus       SIFKGFQEAPIHFYPTYKFDIGCDIYDTTSKQRTPSYTDRILFRNRQTDD---------- 573
A0A3Q1CXU7_Amphiprion_ocellaris              SIFKGFQEAPIHFYPTYKFDIGCDIYDTTSKQRTPSYTDRILFRNRQTDD---------- 566
A0A3P8SMU2_Amphiprion_percula                SIFKGFQEAPITFYPTYKFDIGCDIYDTTSKQRTPSYTDRILFRNRQTDD---------- 569
A0A5J5DH05_Etheostoma_spectabile             SIFKGFQEAPIHFLPTYKFDIGCDIYDTTSKQRTPSYTDRILFRSRQAED---------- 566
A0A484DDW2_Perca_flavescens                  SIFKGFQEAPIHFLPTYKFDVGCDIYDTTSKQRTPSYTDRILFRSRQAED---------- 566
A0A4Z2H351_Liparis_tanakae                   SIFKGFQEAPIHFLPTYKFDAGCDIYDTTSKQRTPSYTDRILSRSRQADD---------- 564
W5UJJ6_Ictalurus_punctatus                   SIFKGFQEAPIHFTPTYKFDIGCDVYDTTPKQRTPSYTDRILFRSRQVDD---------- 566
A0A5N5KDW8_Pangasianodon_hypophthalmus       SIFKGFQEAPIHFTPTYKFDIGCDVYDTTPKQRTPSYTDRILFRSRQVDD---------- 534
A0A3P8ZCA3_Esox_lucius                       SIFKGFQEAPIQFLPTYKFDVGCDVYDTTSKQRTPSYTDRILFRNRHVDD---------- 571
A0A4W5L9K3_Hucho_hucho                       SIFKGFQEALIQFLPTYKFDVGCDVYDTTSKQRTPSYTDRILFRNRQVDD---------- 572
A0A1S3NKU9_Salmo_salar                       SIFKGFQEALIQFLPTYKFDVGCDVYDTTSKQRTPSYTDRILFRNRQVDD---------- 577
W5L4Z3_Astyanax_mexicanus                    SIFKGFQEAPIHFPPTYKFDIGCDVYDSTSKQRTPSYTDRILYRNRQADD---------- 565
A0A3B4C5P1_Pygocentrus_nattereri             SIFKGFQEAPIHFPPTYKFDIGCDIYDSTSKQRTPSYTDRILYRNRQADD---------- 567
H3BEM7_Latimeria_chalumnae                   SIFNGFREAVIGFLPTYKFDVGCDTYDSSAKQRIPSYTDRIVYKSRQQHD---------- 614
A0A1L8F610_Xenopus_laevis                    TIFKGFQEAEIQFLPTYKFDIGCDVYDSSSKQRTPSYTDRVVYKSRNKDD---------- 551
K7FPA9_Pelodiscus_sinensis                   AIFKGFQEAAIHFRPSYKFDLGQDSYDTTSKQRTPSYTDRVMYRSRHKDD---------- 579
A0A452GIT0_Gopherus_agassizii                AIFKGFQEAAIHFRPSYKFDIGQDSYDTTSKQRTPSYTDRVIYRSRYKDD---------- 580
G3VXX0_Sarcophilus_harrisii                  SIFKGFREADIHFFPSYKFDVGKDSYDSTSKQRTPSYTDRVVYKSRQKDD---------- 580
A0A4X2KFU4_Vombatus_ursinus                  SIFKGFREADIHFFPSYKFDVGKDSYDSTSKQRTPSYTDRVVYKSRQKDD---------- 580
G1N6K2_Meleagris_gallopavo                   SIFKGFQEADIHFRPSYKFDIGKDSYDTTSKQRTPSYTDRVVFRSRYRDD---------- 514
F1P5J6_Gallus_gallus                         SIFKGFQEADIHFRPSYKFDIGKDSYDTTSKQRTPSYTDRVVFRSRYRDD---------- 582
U3IH52_Anas_platyrhynchos_platyrhynchos      SIFKGFQEADIRFRPSYKFDIGKDSYDTTSKQRTPSYTDRVVFRSRYRDD---------- 562
A0A151MFV8_Alligator_mississippiensis        SVFKGFQEAAIHFHPSYKFDIGQDCYDTTSKQRTPSYTDRVVYRSRHKND---------- 373
A0A1U7RBH0_Alligator_sinensis                SVFKGFQEAAIHFHPSYKFDIGQDCYDTTSKQRTPSYTDRVVYRSRHKND---------- 541
U3JGA7_Ficedula_albicollis                   SIFKGFQEADIHFRPSYKFDIGKDSYDSTSKQRTPSYTDRVVFRSRYKDD---------- 577
A0A3L8SD89_Chloebia_gouldiae                 SIFKGFQEAAIHFRPSYKFDIGKDSYDTTSKQRTPSYTDRVVFRSRYKDD---------- 559
H0Z776_Taeniopygia_guttata                   SIFKGFQEAAIHFRPSYKFDIGKDSYDTTSKQRTPSYTDRVVFRSRYKDD---------- 517
A0A218V5X6_Lonchura_striata_domestica        SIFKGFQEAAIHFRPSYKFDIGKDSYDTTSKQRTPSYTDRVVFRSRYKDD---------- 577
A0A0Q3MLL4_Amazona_aestiva                   SIFKGFQEADIRFRPSYKFDIGKDSYDTTSKQRTPSYTDRVVFRSRYRDD---------- 582
A0A1V4J6C2_Patagioenas_fasciata_monilis      SIFKGFQEADIHFRPSYKFDIGKDSYDTTSKQRTPSYTDRVVYRSRYKDD---------- 579
A0A2I0M3Z8_Columba_livia                     SIFKGFQEADIHFRPSYKFDIGKDSYDTTSKQRTPSYTDRVVYRSRYKDD---------- 579
INP5E_Drosophila_melanogaster                AAFRGFMEANITFPPTYKYDPGSQNFDTSSKQRAPAYTDRILYKYRQMQGLVIRRQTLVP 669
                                                            :   *       ::  :  *:****:: : *   .

INP5E_Homo_sapiens                           ---------ICPVSYSSCPGIKTSDHRPVYGLFRVKVRPGRDNIPLAAGKFDRELYLLGI 618
INP5E_Pan_troglodytes                        ---------ICPVSYSSCPGIKTSDHRPVYGLFRVKVRPGRDNIPLAAGKFDRELYLLGI 618
A0A2K5K2A0_Colobus_angolensis_palliatus      ---------ICPVIYSSCPGIKTSDHRPVYGLFRVKVRPGRDNIPLAAGKFDRELYLLGI 547
A0A2K6PY13_Rhinopithecus_roxellana           ---------ICPVIYSSCPGIKTSDHRPVYGLFRVKVRPGRDNIPLAAGKFDRELYLLGI 619
H2PTZ0_Pongo_abelii                          ---------ICPVSYSSCPGIKTSDHRPVYGLFRVKVRPGRDNIPLAAGKFDRELYLLGI 615
A0A2K6U2B2_Saimiri_boliviensis_boliviensis   ---------ICPVRYSSCAGIRTSDHRPVYGLFRVKVRPGRDNIPLAAGKFDRELYLIGI 603
A0A2K6F2I5_Propithecus_coquereli             ---------ICPVKYASCPGIKTSDHRPVYGLFRVKVRPGRDNIPLAAGKFDRELYLIGI 616
A0A2K6KCW3_Rhinopithecus_bieti               ---------ICPVIYSSCPGIKTSDHRPVYGLFRVKVRPGRDNIPLAAGKFDRELYLLGI 511
A0A2K5MDJ3_Cercocebus_atys                   ---------ICPVSYSSCPGIKTSDHRPVYGLFRVKVRPGRDNIPLAAGKFDRELYLLGI 617
A0A2K5XR75_Mandrillus_leucophaeus            ---------ICPVSYSSCPGIKTSDHRPVYGLFRVKVRPGRDNIPLAAGKFDRELYLLGI 351
A0A0D9RTF7_Chlorocebus_sabaeus               ---------ICPVIYSSCPGIKTSDHRPVYGLFRVKVRPGRDNIPLAAGKFDRELYLLGI 618
A0A096NRS4_Papio_anubis                      ---------ICPVSYSSCPGIKTSDHRPVYGLFRVKVRPGRDNIPLAAGKFDRELYLLGI 617
A0A2K5U0T2_Macaca_fascicularis               ---------ICPVSYSSCPGIKTSDHRPVYGLFRVKVRPGRDNIPLAAGKFDRELYLLGI 618
F7DRP8_Macaca_mulatta                        ---------ICPVSYSSCPGIKTSDHRPVYGLFRVKVRPGRDNIPLAAGKFDRELYLLGI 618
A0A2K5PHS4_Cebus_capucinus_imitator          ---------ICPVRYSSCAGIRTSDHRPVYGLFRVKVRPGRDNIPLAAGKFDRELYLIGI 612
A0A2K5CN95_Aotus_nancymaae                   ---------ICPVRYSSCAGIRTSDHRPVYGLFRVKVRPGRDNIPLAAGKFDRELYLIGI 620
A0A3Q0DNV4_Tarsius_syrichta                  ---------ICPVRYSSCPGIKTSDHRPVYGLFRVKVRPGRDNIPLAAGKFDRELYLLGI 347
H0XE73_Otolemur_garnettii                    ---------ICPVKYSSCPGIKTSDHRPVYGLFRVKVRPGRDNIPLAAGKFDRELYLIGI 622
I3N2L4_Ictidomys_tridecemlineatus            ---------ICPVKYSSCPGIKTSDHRPVYGLFRVKVRPGRDNIPLAAGKFDRELYLIGI 547
F7BT66_Callithrix_jacchus                    ---------ICPVRYSSCTGIRTSDHRPVYGLFRVKVRPGRDNIPLAAGKFDRELYLIGI 620
A0A250Y3V1_Castor_canadensis                 ---------ISPLKYSSCPGIKTSDHRPVYGLFRVKVKPGRDNIPLAAGKFDRELYLIGI 612
A0A1S3G993Dipodomys_ordii                    ---------ICPLKYSSCLGIKTSDHRPVYGLFRVKVRPGRDNIPLAAGKFDRELYLIGI 564
H0VEZ3_Cavia_porcellus                       ---------IHPVTYSSCPGIRTSDHRPVYGLFRVRVRPGRDNIPLAAGKFDRELYLVGI 620
G5B6B1_Heterocephalus_glaber                 ---------ICPVIYSSCPGIKTSDHRPVYGLFRVRVRPGRDNIPLAAGKFDRELYLLGI 624
A0A2Y9E841_Trichechus_manatus_latirostris    ---------ICPVKYSSCPSIRTSDHRPVYGLFRVRVRPGRDNIPLAAGKFDRELYLIGI 599
L5KA82_Pteropus_alecto                       ---------ICPLKYSSCPGIRTSDHRPVYGLFRVKVRPGRDNIPLAAGKFDRELYLIGI 594
A0A5N4EAQ5_Camelus_dromedarius               ---------IRPIKYASCPGIRTSDHRPVYGLFRVRVRPGRDNIPLAAGKFDRELYLIGI 630
A0A3Q2HCU7_Equus_caballus                    ---------ICPVKYSCCPGIRTSDHRPVYGLFRVKVRPGRDNIPLAAGKFDRELYLIGI 627
A0A1S2ZM74_Erinaceus_europaeus               ---------ICALKYASCPGVKTSDHRPVYGLFRVKVRPGRDNIPLAAGKFDRELYLLGI 621
A0A3Q0DCZ7_Mesocricetus_auratus              ---------ICPMKYSSCPGLKTSDHRPVYGLFRVKVRPGRDNIPLAAGKFDRELYLIGI 613
G3I6Z4_Cricetulus_griseus                    ---------ICPMKYSSCPGIKTSDHRPVYGLFRVKVRPGRDNIPLAAGKFDRELYLIGI 613
INP5E_Rattus_norvegicus                      ---------ICPMKYSSCPGIKTSDHRPVYGLFRVKVRPGRDNIPLAAGKFDRELYLIGI 622
INP5E_Mus_musculus                           ---------ICPMKYSSCPGIKTSDHRPVYGLFQVKVRPGRDNIPLAAGKFDRELYLIGI 621
A0A2Y9NRK2_Delphinapterus_leucas             ---------ICPVKYSSCPGIRTSDHRPVYGLFRVRVRPGRDNIPLAAGKFDRELYLIGI 601
A0A2Y9SXS0_Physeter_macrocephalus            ---------ICPVKYSSCPGIRTSDHRPVYGLFRVRVRPGRDNIPLAAGKFDRELYLIGI 586
E1BAU6_Bos_taurus                            ---------ICPVKYSSCPGVRTSDHRPVYGLFRVKVRPGRDNIPLAAGKFDRDLYLLGI 630
```

```
A0A4W2BWU9_Bos_indicus_x_Bos_taurus         ---------ICPVKYSSCPGVRTSDHRPVYGLFRVKVRPGRDNIPLAAGKFDRDLYLLGI 630
L8IYW6_Bos_mutus                            ---------ICPVKYSSCPGVRTSDHRPVYGLFRVKVRPGRDNIPLAAGKFDRDLYLLGI 630
A0A452DQV4_Capra_hircus                     ---------ICPIKYSSCPGVRTSDHRPVYGLFRVKVRPGRDNIPLAAGKFDRELYLLGI 630
A0A5J5MJZ6_Muntiacus_reevesi                ---------ICPVKYSSCPGIRTSDHRPVYGLFRVKVRPGRDNIPLAAGKFDRELYLLGI 630
A0A5N3WU40_Muntiacus_muntjak                ---------ICPVKYSSCPGIRTSDHRPVYGLFRVKVRPGRDNIPLAAGKFDRELYLLGI 630
A0A3Q7V225_Ursus_arctos_horribilis          ---------ICPVKYSSCSGIRTSDHRPVYGLFRVKVRPGRDNIPLAAGKFDRELYLIGI 616
A0A452Q8J6_Ursus_americanus                 ---------ICPVKYSSCPGIRTSDHRPVYGLFRVKVRPGRDNIPLAAGKFDRELYLIGI 616
A0A452VEA9_Ursus_maritimus                  ---------ICPVKYSSCPGIRTSDHRPVYGLFRVKVRPGRDNIPLAAGKFDRELYLIGI 618
A0A2U3ZR12_Odobenus_rosmarus_divergens      ---------ICPIKYSSCPGIKTSDHRPVYGLFRVKVRPGRDNIPLAAGKFDRELYLIGI 586
A0A3Q7N7C7_Callorhinus_ursinus              ---------ICPIKYSSCPGIKTSDHRPVYGLFRVKVRPGRDNIPLAAGKFDRELYLIGI 586
A0A2Y9HBQ8_Neomonachus_schauinslandi        ---------ICPIKYSSCPGIKTSDHRPVYGLFRVKVRPGRDNIPLAAGKFDRELYLIGI 587
A0A485NC41_Lynx_pardinus                    ---------ICPVKYSSCPGIKTSDHRPVYGLFRVKVRPGRDNIPLAAGKFDRELYLIGI 610
A0A3Q7QUW1_Vulpes_vulpes                    ---------ICPVRYSSCPGIKTSDHRPVYGLFRVKVRPGRDNIPLAAGKFDRELYLIGI 587
F1PDA7_Canis_lupus_familiaris               ---------ICPVRYSSCPGIKTSDHRPVYGLFRVKVRPGRDNIPLAAGKFDRELYLIGI 596
A0A2Y9JFT0_Enhydra_lutris_kenyoni           ---------ICPIKYSSCPGIKTSDHRPVYSLFRVKVRPGRDNIPLAAGKFDRELYLIGI 616
M3YJ90_Mustela_putorius_furo                ---------ICPIKYSSCPGIKTSDHRPVYGLFRVKVRPGRDNIPLAAGKFDRELYLIGI 616
I3JX61_Oreochromis_niloticus                ---------IKTLMYTSCSNIKTSDHRPVIGVFQVKLRPGRDNIPLCAGQFDRSLYLEGI 615
A0A3Q4H5I7_Neolamprologus_brichardi         ---------IKTLMYTSCSNIKTSDHRPVIGVFQVKLRPGRDNIPLCAGQFDRSLYLEGI 615
A0A3B4FRA1_Pundamilia_nyererei              ---------IKTLMYTSCSNIKTSDHRPVIGVFQVKLRPGRDNIPLCAGQFDRSLYLEGI 615
A0A3P8QQM7_Astatotilapia_calliptera         ---------IKTLMYTSCSNIKTSDHRPVIGVFQVKLRPGRDNIPLCAGQFDRSLYLEGI 615
W5M6X5_Lepisosteus_oculatus                 ---------IKVMKYACCPTIKTSDHRPVYAVFQVKVRPGRDNIPLGAGQFDRNLYLEGI 566
A0A3B3RRU9_Paramormyrops_kingsleyae         ---------ITVVKYSCCSSIRTSDHRPVVGIFQVKLRPGRDNIPLGAGQFDRELYLEGI 615
A0A4W4HDW3_Electrophorus_electricus         ---------IKVVKYTSCSSIKTSDHRPVVGMFQVKLRPGRDNIPLGAGQFDRIVYLEGI 613
A0A3Q3IZN0_Monopterus_albus                 ---------IKVVKYTSCSSIKTSDHRPVIGVFRVKLRPGRDNIPLCAGQFDRGLFLEGI 613
A0A3P8VEE4_Cynoglossus_semilaevis           ---------IQVVKYTSCSSIKTSDHRPVIGVFHVKLRPGRDNIPLGAGQFDRALYLEGI 606
A0A3Q1H6D9_Anabas_testudineus               ---------IKVIKYTSCSSIKTSDHRPVIGVFLVKLRPGRDNIPLGAGQFDRGLYLEGI 616
A0A4W6CEK3_Lates_calcarifer                 ---------IKVVKYTSCSSIKTSDHRPVIGVFQVKLRPGRDNIPLGAGQFDRGLYLEGI 616
A0A3B4VK37_Seriola_dumerili                 ---------IKVVKYTSCSSIKTSDHRPVIGVFQVKLRPGRDNIPLGAGQFDRGLYLEGI 616
A0A3Q3GNS7_Labrus_bergylta                  ---------IKVVKYTSCCGIKTSDHRPVIGVFQVKLRPGRDNIPLGAGQFDRGLYLEGI 615
A0A3Q3RRU7_9TELE_Mastacembelus_armatus      ---------IKVVKYTSCSSIKTSDHRPVIGVFHVKLRPGRDNIPLGAGQFDRGLYLEGI 610
A0A4U5UTG8_Collichthys_lucidus              ---------IKVDKYTSCSNIKTSDHRPVIGVFQVKLRPGRDNIPLGAGQFDRGLYLEGI 619
A0A3B4ZI99_Stegastes_partitus               ---------IKVLKYTSCSNIKTSDHRPVIGVFQVKLRPGRDNIPLGAGQFDRGLYLEGI 619
A0A3Q1H1R6_Acanthochromis_polyacanthus      ---------IKVIKYTSCSNIKTSDHRPVIGVFQVKLRPGRDNIPLGAGQFDRGLYLEGI 624
A0A3Q1CXU7_Amphiprion_ocellaris             ---------IKVVKYTSCSNIKTSDHRPVIGVFQVKLRPGRDNIPLGAGQFDRGLYLEGI 617
A0A3P8SMU2_Amphiprion_percula               ---------IKVVKYTSCSNIKTSDHRPVIGVFQVKLRPGRDNIPLGAGQFDRGLYLEGI 620
A0A5J5DH05_Etheostoma_spectabile            ---------IKVVKYTSCSCIKTSDHRPVISVFQVKLRPGRDNIPLGAGQFDRSLYLEGI 617
A0A484DDW2_Perca_flavescens                 ---------IKVVKYTSCSCIKTSDHRPVISVFQVKLRPGRDNIPLGAGQFDRSLYLEGI 617
A0A4Z2H351_Liparis_tanakae                  ---------IQVVKYTSCSSIKTSDHRPVVGVFQVKLRPGRDNIPLGAGQFDRSLYLEGI 615
W5UJJ6_Ictalurus_punctatus                  ---------IKVVKYTSCSSIKTSDHRPVIGMFQVKLRPGRDNIPLGAGLFDRAVYLEGI 617
A0A5N5KDW8_Pangasianodon_hypophthalmus      ---------IKVVKYTSCSSIKTSDHRPVIGMFQVKLRPGRDNIPLGAGLFDRAVYLEGI 585
A0A3P8ZCA3_Esox_lucius                      ---------IKVMKYTSCSTIKTSDHRPVIGMFLVKLRPGRDNIPLGAGQFDRSLYLEGI 622
A0A4W5L9K3_Hucho_hucho                      ---------IKVIKYTSCSMIKTSDHRPVIGMFQVKLRPGRDNIPLGAGQFDRSLYLEGI 623
A0A1S3NKU9_Salmo_salar                      ---------IKVIKYTSCSMIKTSDHRPVIGMFQVKLRPGRDNIPLGAGQFDRSLYLEGI 628
W5L4Z3_Astyanax_mexicanus                   ---------IKVVKYTSCSSIKTSDHRPVIGMFQVKIRPGRDNIPLGAGQFDRPVYLEGI 616
A0A3B4C5P1_Pygocentrus_nattereri            ---------IKVVKYTSCSSIKTSDHRPVIGMFQVKIRPGRDNIPLGAGQFDRPVYLEGI 618
H3BEM7_Latimeria_chalumnae                  ---------IHATKYACCTTFKNSDHRPVYGIFQVKLRPGRDNIPLCAGHFDRVLYLEGI 665
A0A1L8F610_Xenopus_laevis                   ---------IRVLKYASCSLINTSDHRPVFGLFEIRIRPGRDNIPLAAGQFDRELYLLGI 602
K7FPA9_Pelodiscus_sinensis                  ---------IQAVKYSSCSAIRTSDHRPVYGLFRVKVRPGRDNIPLAAGQFDRELYLIGI 630
A0A452GIT0_Gopherus_agassizii               ---------IHAVKYSSCSVVKTSDHRPVYGLFRVKVRPGRDNIPLAAGQFDRELYLIGI 631
G3VXX0_Sarcophilus_harrisii                 ---------IYPVKYSSCPGIKTSDHRPVYGLFRVKVRPGRDNIPLAAGKFDRDLYLLGI 631
A0A4X2KFU4_Vombatus_ursinus                 ---------IYPVKYSSCPGIKTSDHRPVYGLFRVKVRPGRDNIPLAAGKFDRDLYLLGI 631
G1N6K2_Meleagris_gallopavo                  ---------IQAVKYSSCPVIKTSDHRPVFALFRVKVRPGRDNIPLAAGQFDRELYLIGI 565
F1P5J6_Gallus_gallus                        ---------IQAVKYSSCPVIKTSDHRPVFALFRVKVRPGRDNIPLAAGQFDRELYLIGI 633
U3IH52_Anas_platyrhynchos_platyrhynchos     ---------IHAVKYSSCPVIKTSDHRPVFALFRVKVRPGRDNIPLAAGQFDRELYLIGI 613
A0A151MFV8_Alligator_mississippiensis       ---------IHAVKYSSCPIIRTSDHRPVYGLFRVKLRPGRDNIPLAAGQFDRELYLFGI 424
A0A1U7RBH0_Alligator_sinensis               ---------IHAVKYSSCPIIRTSDHRPVYGLFRVKLRPGRDNIPLAAGQFDRELYLIGI 592
U3JGA7_Ficedula_albicollis                  ---------IQAVKYSSCPVIRTSDHRPVFALFRVKVRPGRDNIPLAAGLFDRDLYLIGI 628
A0A3L8SD89_Chloebia_gouldiae                ---------IQAVKYSSCPVIKTSDHRPVFALFRVKVRPGRDNIPLAAGLFDRDLYLIGI 610
H0Z776_Taeniopygia_guttata                  ---------IQAVKYSSCPVIKTSDHRPVFALFRVKVRPGRDNIPLAAGLFDRDLYLIGI 568
A0A218V5X6_Lonchura_striata_domestica       ---------IQAVKYSSCPVIKTSDHRPVFALFRVKVRPGRDNIPLAAGLFDRDLYLIGI 628
A0A0Q3MLL4_Amazona_aestiva                  ---------IHAVKYSSCPVIKTSDHRPVFALFRVKVRPGRDNIPLAAGQFDRELYLIXI 633
A0A1V4J6C2_Patagioenas_fasciata_monilis     ---------IHAVKYSSCPVIKTSDHRPVFALFRVKVRPGRDNIPLAAGQFDRDLYLIGI 630
A0A2I0M3Z8_Columba_livia                    ---------IHAVKYSSCPVIKTSDHRPVFALFRVKVRPGRDNIPLAAGQFDRDLYLIGI 630
INP5E_Drosophila_melanogaster               GVSTPTQPHVQCLLYDSVPSITTSDHKPVWALFRTLIRAGTDAIPLAAGLFSRDIYLEGM 729
                                                     :    * .   . .***:** .:*   :: * * *** ** *.* ::*  :

INP5E_Homo_sapiens                          KR-RISKEIQRQQA-LQSQNSSTICSVS-------------------------------- 644
INP5E_Pan_troglodytes                       KR-RISKEIQRQQA-LQSQNSSTICSVS-------------------------------- 644
A0A2K5K2A0_Colobus_angolensis_palliatus     KR-RISKEIQRQQA-LQSQNSSTICSVS-------------------------------- 573
A0A2K6PY13_Rhinopithecus_roxellana          KR-RISKEIQRQQA-LQSQNSSTICSVS-------------------------------- 645
H2PTZ0_Pongo_abelii                         KR-RISKEIQRQQA-LQSQNSSTICSVS-------------------------------- 641
A0A2K6U2B2_Saimiri_boliviensis_boliviensis  KR-RISKEIQRQQA-LQSQSSSTICSVS-------------------------------- 629
A0A2K6F2I5_Propithecus_coquereli            KR-RISKEIQRQQA-LKNQNSSTICTVS-------------------------------- 642
A0A2K6KCW3_Rhinopithecus_bieti              KR-RISKEIQRQQA-LQSQNSSTICSVS-------------------------------- 537
A0A2K5MDJ3_Cercocebus_atys                  KR-RISKEIQRQQA-LQSQNSSTICSVS-------------------------------- 643
A0A2K5XR75_Mandrillus_leucophaeus           KR-RISKEIQRQQA-LQSQNSSTICSVS-------------------------------- 377
A0A0D9RTF7_Chlorocebus_sabaeus              KR-RISKEIQRQQA-LQSQNSSTICSVS-------------------------------- 644
A0A096NRS4_Papio_anubis                     KR-RISKEIQRQQA-LQSQNSSTICSVS-------------------------------- 643
A0A2K5U0T2_Macaca_fascicularis              KR-RISKEIQRQQA-LQSQNSSTICSVS-------------------------------- 644
```

```
F7DRP8_Macaca_mulatta                      KR-RISKEIQRQQA-LQSQNSSTICSVS-------------------------------- 644
A0A2K5PHS4_Cebus_capucinus_imitator        KR-RISKEIQRQQG-LQSQSSSTICSVS-------------------------------- 638
A0A2K5CN95_Aotus_nancymaae                 KR-RISKEIQRQQA-LQSQSSSTICSVS-------------------------------- 646
A0A3Q0DNV4_Tarsius_syrichta                KR-RISKEIQRQQA-RKTQSSSTVCTVS-------------------------------- 373
H0XE73_Otolemur_garnettii                  KR-RISKEIQRQQA-LKNQSSSTICTVS-------------------------------- 648
I3N2L4_Ictidomys_tridecemlineatus          KR-RISKEIQKQQA-MKNQNPSTICTIS-------------------------------- 573
F7BT66_Callithrix_jacchus                  KR-RISKEIQRQQA-LQNQSSSTICSVS-------------------------------- 646
A0A250Y3V1_Castor_canadensis               KR-RISKEIQKQQA-LKNQNSSTICTVS-------------------------------- 638
A0A1S3G993Dipodomys_ordii                  KR-RISKEMQKQQV-LKNQSSSTVCTVS-------------------------------- 590
H0VEZ3_Cavia_porcellus                     KR-RISKEIQRQQA-LKNQSASAICSVS-------------------------------- 646
G5B6B1_Heterocephalus_glaber               KR-RISKAIQRQQA-LKNQSSSTVCTVS-------------------------------- 650
A0A2Y9E841_Trichechus_manatus_latirostris  KR-RISKEIQRQQA-LKNQNSSAICTVS-------------------------------- 625
L5KA82_Pteropus_alecto                     KR-RISKEMQRQRA-LKTQHSSAVCTVS-------------------------------- 620
A0A5N4EAQ5_Camelus_dromedarius             KR-RISKEMQRQQV-LKNQHSSTICTVS-------------------------------- 656
A0A3Q2HCU7_Equus_caballus                  KR-RISKEIQRQQV-LKNQHSSTVCTVS-------------------------------- 653
A0A1S2ZM74_Erinaceus_europaeus             KR-RISKEIQRQQA-LKSQNSSAICTVS-------------------------------- 647
A0A3Q0DCZ7_Mesocricetus_auratus            KR-RISKELQKQQT-MKNQSSSAVCTVS-------------------------------- 639
G3I6Z4_Cricetulus_griseus                  KR-RISKELQK-QT-MKNQSSSAVCTVS-------------------------------- 638
INP5E_Rattus_norvegicus                    KR-RISKEIQRQEA-LKSQSSSAVCTVS-------------------------------- 648
INP5E_Mus_musculus                         KR-RISKEIQRQEA-LKSQSSSAVCTVS-------------------------------- 647
A0A2Y9NRK2_Delphinapterus_leucas           KR-RISREIQRQQA-LKSQHSSAICTVS-------------------------------- 627
A0A2Y9SXS0_Physeter_macrocephalus          KR-RISREIQRQQA-LKSQHSSAICTVS-------------------------------- 612
E1BAU6_Bos_taurus                          KR-RISREIQRQQA-LKSQHASAICTVS-------------------------------- 656
A0A4W2BWU9_Bos_indicus_x_Bos_taurus        KR-RISREIQRQQA-LKSQHASAICTVS-------------------------------- 656
L8IYW6_Bos_mutus                           KR-RISREIQRQQA-LKSQHASAICTVS-------------------------------- 656
A0A452DQV4_Capra_hircus                    KR-RISREIQRQQA-LKSQHASAICTIS-------------------------------- 656
A0A5J5MJZ6_Muntiacus_reevesi               KR-RISREIQRQQA-LKSQHASAICTVS-------------------------------- 656
A0A5N3WU40_Muntiacus_muntjak               KR-RISREIQRQQA-LKSQHASAICTVS-------------------------------- 656
A0A3Q7V225_Ursus_arctos_horribilis         KR-RISKEIQRQQA-LKNQHSSTICTVS-------------------------------- 642
A0A452Q8J6_Ursus_americanus                RR-RISKEIRRQQA-LKNQHSSTICTVS-------------------------------- 642
A0A452VEA9_Ursus_maritimus                 KR-RISKEIQRQQA-LKNQHSSTICTVS-------------------------------- 644
A0A2U3ZR12_Odobenus_rosmarus_divergens     KR-RISKEIQRQQA-LKNQHSSTICTVS-------------------------------- 612
A0A3Q7N7C7_Callorhinus_ursinus             KR-RISKEIQRQQA-LKNQHSSTICTVS-------------------------------- 612
A0A2Y9HBQ8_Neomonachus_schauinslandi       KR-RISKEIQRQQA-LKNQHSSTICTVS-------------------------------- 613
A0A485NC41_Lynx_pardinus                   KR-RISKEIQQQQV-PKDQHSSTICTVS-------------------------------- 636
A0A3Q7QUW1_Vulpes_vulpes                   KR-RISKEIQRQQA-LKNQHSSTICTVS-------------------------------- 613
F1PDA7_Canis_lupus_familiaris              KR-RISKEIQRQQA-LKNQHSSTICTVS-------------------------------- 622
A0A2Y9JFT0_Enhydra_lutris_kenyoni          KR-RISREIQRQQA-LKAQHSSSICTVS-------------------------------- 642
M3YJ90_Mustela_putorius_furo               KR-RISKEIQRQQA-LKGQHSSSICTVS-------------------------------- 642
I3JX61_Oreochromis_niloticus               RRRRITREIKTKEA-MKNQS-STICTIS-------------------------------- 641
A0A3Q4H5I7_Neolamprologus_brichardi        RRRRITREIKTKEA-MKNQS-STICTIS-------------------------------- 641
A0A3B4FRA1_Pundamilia_nyererei             RRRRITREIKTKEA-MKNQS-STICTIS-------------------------------- 641
A0A3P8QQM7_Astatotilapia_calliptera        RRRRITREIKTKEA-MKNQS-STICTIS-------------------------------- 641
W5M6X5_Lepisosteus_oculatus                RR-RITRELQRKEA-LKNQKNSVVCSVS-------------------------------- 592
A0A3B3RRU9_Paramormyrops_kingsleyae        RR-RISRELKRREA-MKSQKNSTVCSIS-------------------------------- 641
A0A4W4HDW3_Electrophorus_electricus        RR-RMTRELKRREVVMKNQS--AVCSIS-------------------------------- 638
A0A3Q3IZN0_Monopterus_albus                RR-RITRELKR-EA-TKNQSNSTICAIS-------------------------------- 638
A0A3P8VEE4_Cynoglossus_semilaevis          RR-RITRELKRREA-MKNQSNSSICSIS-------------------------------- 632
A0A3Q1H6D9_Anabas_testudineus              RR-RITRELKRREA-MKSQSSSTICTIS-------------------------------- 642
A0A4W6CEK3_Lates_calcarifer                RR-RITRELKRREA-MKNQSSSTICAIA-------------------------------- 642
A0A3B4VK37_Seriola_dumerili                RR-RITRELKRREA-MKNQGSSTICAIS-------------------------------- 642
A0A3Q3GNS7_Labrus_bergylta                 RR-RITRELKRREA-MKNQGSSTICTIS-------------------------------- 641
A0A3Q3RRU7_9TELE_Mastacembelus_armatus     RR-RITRELKRREA-MKSQGSSTICTLS-------------------------------- 636
A0A4U5UTG8_Collichthys_lucidus             RR-RITRELKRREA-MKNQSSSAICAIS-------------------------------- 645
A0A3B4ZI99_Stegastes_partitus              RR-RITRELKRREA-MKNQSSSTICTIS-------------------------------- 645
A0A3Q1H1R6_Acanthochromis_polyacanthus     RR-RITRELKRREA-MKNQSSSTICTIS-------------------------------- 650
A0A3Q1CXU7_Amphiprion_ocellaris            RR-RITRELKRREA-MKNQSSSTICTIS-------------------------------- 643
A0A3P8SMU2_Amphiprion_percula              RR-RITRELKRREA-MKNQSSSTICTIS-------------------------------- 646
A0A5J5DH05_Etheostoma_spectabile           RR-RITRELKRREA-MKNHSSSTICTIS-------------------------------- 643
A0A484DDW2_Perca_flavescens                RR-RITRELKRREA-MKNHSSSAICTIS-------------------------------- 643
A0A4Z2H351_Liparis_tanakae                 RR-RITRELKRREA-MKSQSSSSICTVS-------------------------------- 641
W5UJJ6_Ictalurus_punctatus                 KR-RITRELKRREA-MKNQSNSTVCSIS-------------------------------- 643
A0A5N5KDW8_Pangasianodon_hypophthalmus     RR-RITRELKRREA-MKNQSNSTVCSIS-------------------------------- 611
A0A3P8ZCA3_Esox_lucius                     RR-RITRELKKREA-MKNQASSTICSIS-------------------------------- 648
A0A4W5L9K3_Hucho_hucho                     RR-RITRELKKREA-TKNQGSSTICSIS-------------------------------- 649
A0A1S3NKU9_Salmo_salar                     RR-RITRELKKREA-TKDQGNSTICSIS-------------------------------- 654
W5L4Z3_Astyanax_mexicanus                  RR-RITRELKRREAVMKNQSNSTVCSIS-------------------------------- 643
A0A3B4C5P1_Pygocentrus_nattereri           KR-RITRELKRREAVMKNQSNSTVCSIS-------------------------------- 645
H3BEM7_Latimeria_chalumnae                 KR-RLAREISRSMS-IKSHRDSLICTIA-------------------------------- 691
A0A1L8F610_Xenopus_laevis                  KR-RSTRKVQHRQL-EKNQKTSSVCSIS-------------------------------- 628
K7FPA9_Pelodiscus_sinensis                 KR-RITRELQKRQE-LKDQKSSRVCAVS-------------------------------- 656
A0A452GIT0_Gopherus_agassizii              KR-RITRKLQKRQE-IKDQKSSRVCTVS-------------------------------- 657
G3VXX0_Sarcophilus_harrisii                KR-RISRELQKQQT-LKDQKSSMICTIS-------------------------------- 657
A0A4X2KFU4_Vombatus_ursinus                KR-RISRELQKQQA-LKDQKSSMICTVS-------------------------------- 657
G1N6K2_Meleagris_gallopavo                 KR-RITRELQNRRQ-QKDQKSSSICSIS-------------------------------- 591
F1P5J6_Gallus_gallus                       KR-RITRELQNRRQ-QKDQKSSSICSIS-------------------------------- 659
U3IH52_Anas_platyrhynchos_platyrhynchos    KR-RITRDVQNRRV-QKDQKSSSICSIS-------------------------------- 639
A0A151MFV8_Alligator_mississippiensis      KR-RITRDLKKRQE-LKDQKSSRVCTVS-------------------------------- 450
A0A1U7RBH0_Alligator_sinensis              KR-RITRDLKKRQE-LKDQKSSRVCTVS-------------------------------- 618
U3JGA7_Ficedula_albicollis                 RR-RATRELLKRRE-QKDQRSSSICSIS-------------------------------- 654
A0A3L8SD89_Chloebia_gouldiae               RR-RATRELLKRRE-QKDQRSSSIYYLRKPLGSRPLVPLHAGAQLLELTSSRAGDPRKAC 668
```

```
H0Z776_Taeniopygia_guttata              RR-RATRELLKRRE-QKDQRSSSICSIS-------------------------------- 594
A0A218V5X6_Lonchura_striata_domestica   RR-RATRELLKRRE-QKDQRSSSICSIS-------------------------------- 654
A0A0Q3MLL4_Amazona_aestiva              KR-RITRELQKRKE-QKDQKSSSICSIS-------------------------------- 659
A0A1V4J6C2_Patagioenas_fasciata_monilis KR-RITRELQKRQE-QKDQRSSSICSIS-------------------------------- 656
A0A2I0M3Z8_Columba_livia                KR-RITRELQKRQE-QKDQRSSSICSIS-------------------------------- 656
INP5E_Drosophila_melanogaster           RR-RLNNQYS---------GASAVCVLQ-------------------------------- 747
                                        :* *  .             :  :

INP5E_Homo_sapiens                      ------------------------
INP5E_Pan_troglodytes                   ------------------------
A0A2K5K2A0_Colobus_angolensis_palliatus ------------------------
A0A2K6PY13_Rhinopithecus_roxellana      ------------------------
H2PTZ0_Pongo_abelii                     ------------------------
A0A2K6U2B2_Saimiri_boliviensis_boliviensis ------------------------
A0A2K6F2I5_Propithecus_coquereli        ------------------------
A0A2K6KCW3_Rhinopithecus_bieti          ------------------------
A0A2K5MDJ3_Cercocebus_atys              ------------------------
A0A2K5XR75_Mandrillus_leucophaeus       ------------------------
A0A0D9RTF7_Chlorocebus_sabaeus          ------------------------
A0A096NRS4_Papio_anubis                 ------------------------
A0A2K5U0T2_Macaca_fascicularis          ------------------------
F7DRP8_Macaca_mulatta                   ------------------------
A0A2K5PHS4_Cebus_capucinus_imitator     ------------------------
A0A2K5CN95_Aotus_nancymaae              ------------------------
A0A3Q0DNV4_Tarsius_syrichta             ------------------------
H0XE73_Otolemur_garnettii               ------------------------
I3N2L4_Ictidomys_tridecemlineatus       ------------------------
F7BT66_Callithrix_jacchus               ------------------------
A0A250Y3V1_Castor_canadensis            ------------------------
A0A1S3G993Dipodomys_ordii               ------------------------
H0VEZ3_Cavia_porcellus                  ------------------------
G5B6B1_Heterocephalus_glaber            ------------------------
A0A2Y9E841_Trichechus_manatus_latirostris ------------------------
L5KA82_Pteropus_alecto                  ------------------------
A0A5N4EAQ5_Camelus_dromedarius          ------------------------
A0A3Q2HCU7_Equus_caballus               ------------------------
A0A1S2ZM74_Erinaceus_europaeus          ------------------------
A0A3Q0DCZ7_Mesocricetus_auratus         ------------------------
G3I6Z4_Cricetulus_griseus               ------------------------
INP5E_Rattus_norvegicus                 ------------------------
INP5E_Mus_musculus                      ------------------------
A0A2Y9NRK2_Delphinapterus_leucas        ------------------------
A0A2Y9SXS0_Physeter_macrocephalus       ------------------------
E1BAU6_Bos_taurus                       ------------------------
A0A4W2BWU9_Bos_indicus_x_Bos_taurus     ------------------------
L8IYW6_Bos_mutus                        ------------------------
A0A452DQV4_Capra_hircus                 ------------------------
A0A5J5MJZ6_Muntiacus_reevesi            ------------------------
A0A5N3WU40_Muntiacus_muntjak            ------------------------
A0A3Q7V225_Ursus_arctos_horribilis      ------------------------
A0A452Q8J6_Ursus_americanus             ------------------------
A0A452VEA9_Ursus_maritimus              ------------------------
A0A2U3ZR12_Odobenus_rosmarus_divergens  ------------------------
A0A3Q7N7C7_Callorhinus_ursinus          ------------------------
A0A2Y9HBQ8_Neomonachus_schauinslandi    ------------------------
A0A485NC41_Lynx_pardinus                ------------------------
A0A3Q7QUW1_Vulpes_vulpes                ------------------------
F1PDA7_Canis_lupus_familiaris           ------------------------
A0A2Y9JFT0_Enhydra_lutris_kenyoni       ------------------------
M3YJ90_Mustela_putorius_furo            ------------------------
I3JX61_Oreochromis_niloticus            ------------------------
A0A3Q4H5I7_Neolamprologus_brichardi     ------------------------
A0A3B4FRA1_Pundamilia_nyererei          ------------------------
A0A3P8QQM7_Astatotilapia_calliptera     ------------------------
W5M6X5_Lepisosteus_oculatus             ------------------------
A0A3B3RRU9_Paramormyrops_kingsleyae     ------------------------
A0A4W4HDW3_Electrophorus_electricus     ------------------------
A0A3Q3IZN0_Monopterus_albus             ------------------------
A0A3P8VEE4_Cynoglossus_semilaevis       ------------------------
A0A3Q1H6D9_Anabas_testudineus           ------------------------
A0A4W6CEK3_Lates_calcarifer             ------------------------
A0A3B4VK37_Seriola_dumerili             ------------------------
A0A3Q3GNS7_Labrus_bergylta              ------------------------
A0A3Q3RRU7_9TELE_Mastacembelus_armatus  ------------------------
A0A4U5UTG8_Collichthys_lucidus          ------------------------
A0A3B4ZI99_Stegastes_partitus           ------------------------
A0A3Q1H1R6_Acanthochromis_polyacanthus  ------------------------
A0A3Q1CXU7_Amphiprion_ocellaris         ------------------------
A0A3P8SMU2_Amphiprion_percula           ------------------------
A0A5J5DH05_Etheostoma_spectabile        ------------------------
A0A484DDW2_Perca_flavescens             ------------------------
```

```
A0A4Z2H351_Liparis_tanakae                 ------------------------
W5UJJ6_Ictalurus_punctatus                 ------------------------
A0A5N5KDW8_Pangasianodon_hypophthalmus     ------------------------
A0A3P8ZCA3_Esox_lucius                     ------------------------
A0A4W5L9K3_Hucho_hucho                     ------------------------
A0A1S3NKU9_Salmo_salar                     ------------------------
W5L4Z3_Astyanax_mexicanus                  ------------------------
A0A3B4C5P1_Pygocentrus_nattereri           ------------------------
H3BEM7_Latimeria_chalumnae                 ------------------------
A0A1L8F610_Xenopus_laevis                  ------------------------
K7FPA9_Pelodiscus_sinensis                 ------------------------
A0A452GIT0_Gopherus_agassizii              ------------------------
G3VXX0_Sarcophilus_harrisii                ------------------------
A0A4X2KFU4_Vombatus_ursinus                ------------------------
G1N6K2_Meleagris_gallopavo                 ------------------------
F1P5J6_Gallus_gallus                       ------------------------
U3IH52_Anas_platyrhynchos_platyrhynchos    ------------------------
A0A151MFV8_Alligator_mississippiensis      ------------------------
A0A1U7RBH0_Alligator_sinensis              ------------------------
U3JGA7_Ficedula_albicollis                 ------------------------
A0A3L8SD89_Chloebia_gouldiae               RKLSHISPVRLTVSSFCKALSPSG 692
H0Z776_Taeniopygia_guttata                 ------------------------
A0A218V5X6_Lonchura_striata_domestica      ------------------------
A0A0Q3MLL4_Amazona_aestiva                 ------------------------
A0A1V4J6C2_Patagioenas_fasciata_monilis    ------------------------
A0A2I0M3Z8_Columba_livia                   ------------------------
INP5E_Drosophila_melanogaster              ------------------------
```

Supplementary Fig. 1 INPP5E protein alignment in 100 different species. Evolutionarily conserved residues are indicated at the bottom of the alignment by using the symbols period (.), colon (:), and asterisk (*), consistent with increasing conservation. Clustal Omega predicted domain (residues 297-599 of human INPP5E) is indicated by a dark green bar and it overlaps with the Pfam-predicted phosphatase domain (residues 304-584). However, due to the significant conservation of region encompassing residues 273-296 and 600-621, we redefined the length of the INPP5E functional domain and indicated it by a light green bar. Residues mutated in the IRD probands presented in this study are highlighted in bold red.

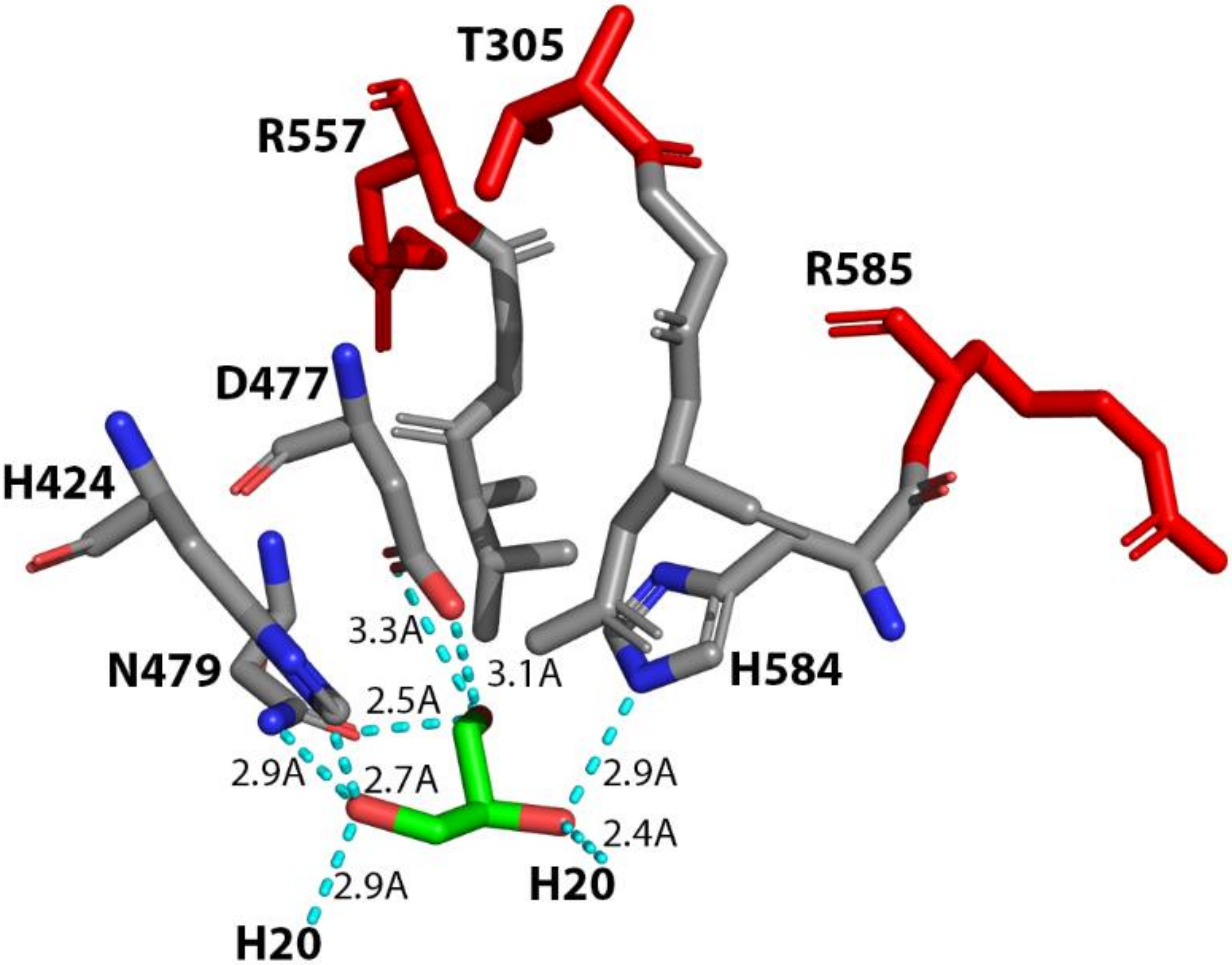

Supplementary Fig. 2 Protein modelling of the known INPP5E catalytic domain.
One glycerol molecule (highlighted in green) is predicted to form polar bonds (cyan dashed lines) with residues His424, Asn479, Asp477, and His584. Three of the likely pathogenic variants identified in this study (highlighted in red) are located either within the catalytic pocket: p.(Arg557His) and p.(Arg585His) or in its proximity: p.(Thr305Ile).

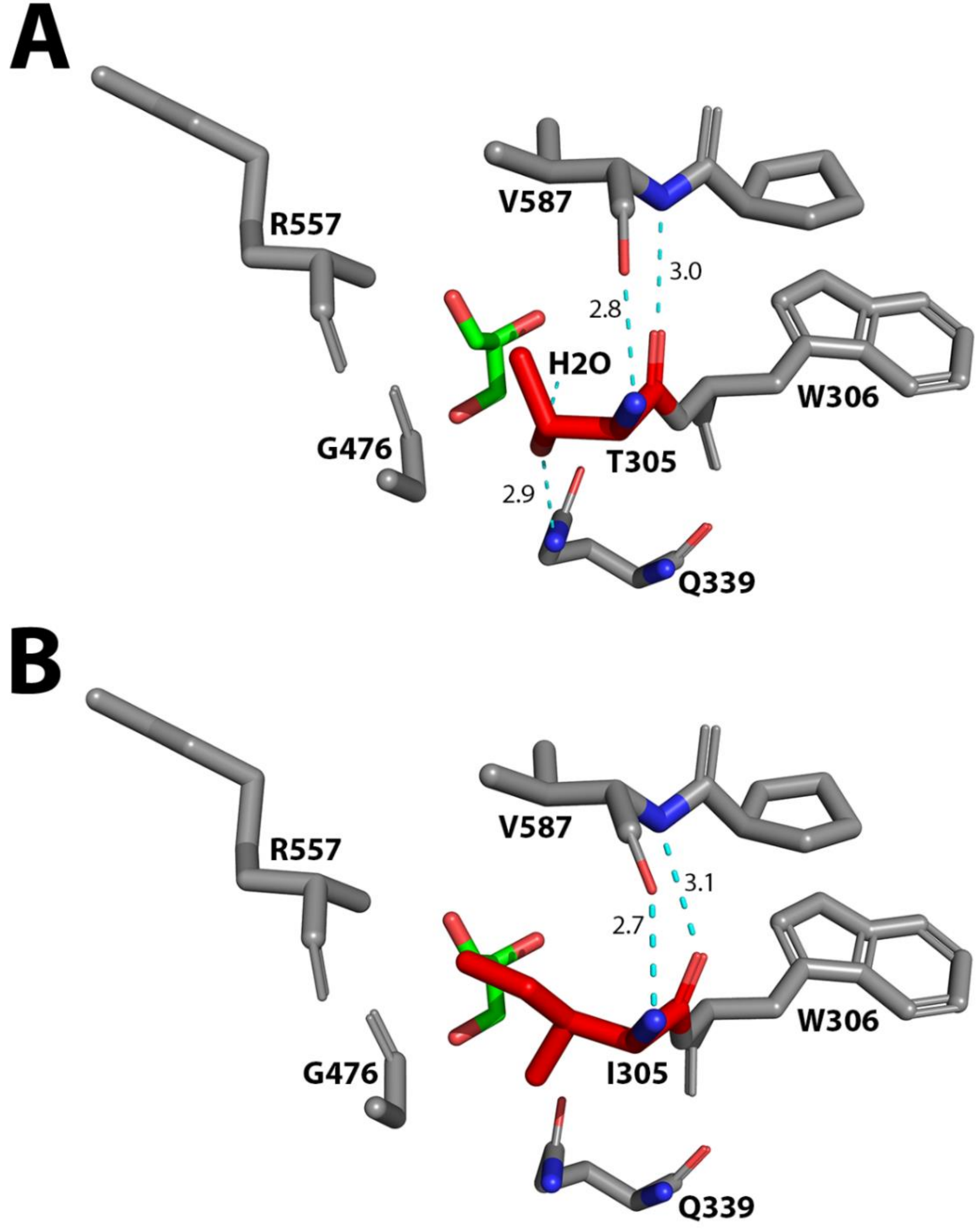

Supplementary Fig. 3 Prediction of the structural changes caused by the p.(Thr305Ile) variant.
(A) The Thr305 is predicted to form polar bonds (cyan dashed lines) with residues Val587 and Gln339. **(B)** The p.(Thr305Ile) variant leads to the disruption of the polar bond with Gln339 but not of the ones with Val587.

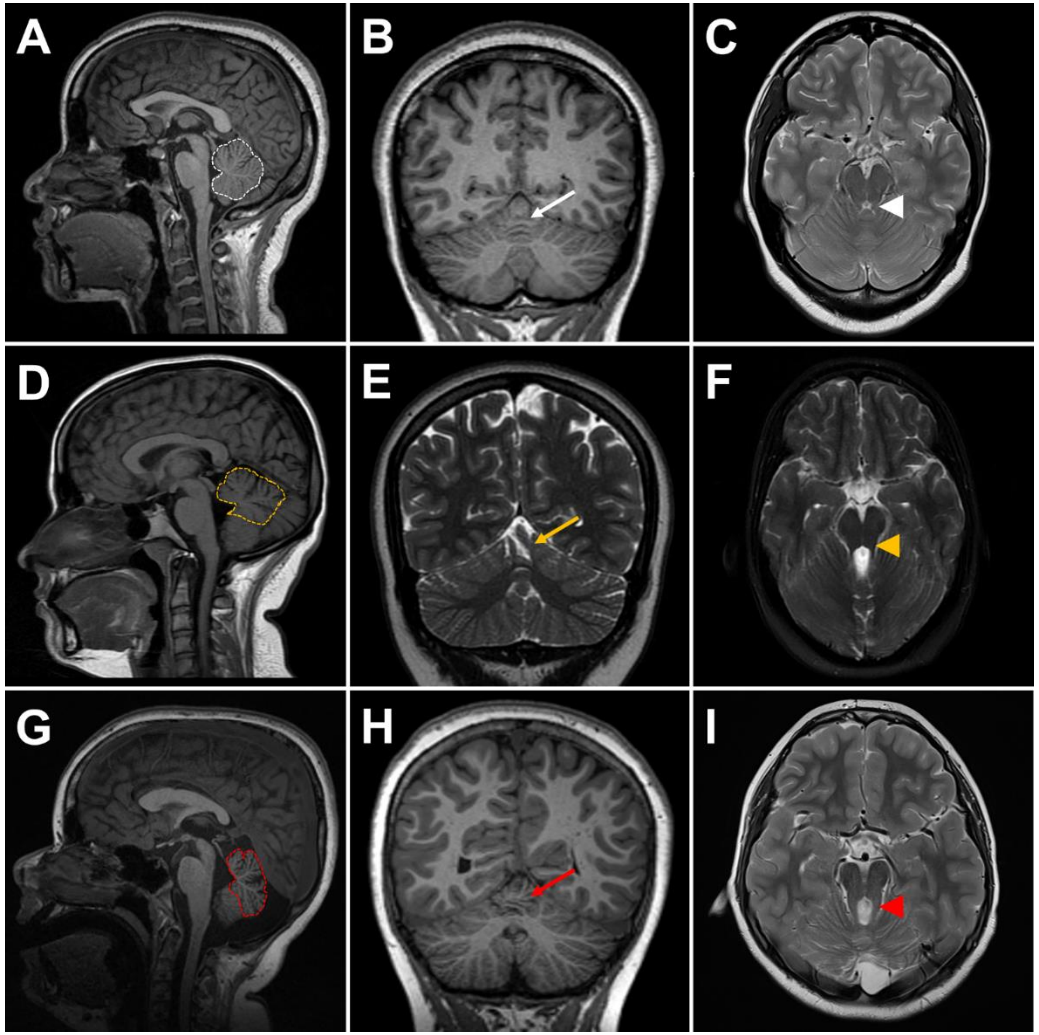

Supplementary Fig. 4 MRI comparison between healthy subject, proband LL135 and patient with Joubert Syndrome (JBTS).

MRI comparison between a healthy age and sex-matched subject **(A-C)**, proband C.II.1 **(D-F),** and one patient with JBTS **(G-I)** demonstrated mild vermian hypoplasia (**D**, orange dashed line) on sagittal plane compared to JBTS patient who had severe vermian hypoplasia, also referred to as molar tooth sign (**G**, red dashed line). On coronal images, a dysplastic appearance of the superior vermian folia (**E**, orange arrow) was additionally seen, as in JBTS patient (**H**, red arrow). Compared to healthy subject who had short vertically oriented superior cerebellar peduncles (SCP, white arrow head), proband C.II-3 presented with thin elongated horizontally-oriented SCP (**F**, orange arrow head) and JBTS patient with very thick elongated horizontally-oriented SCP (**I**, red arrow head).

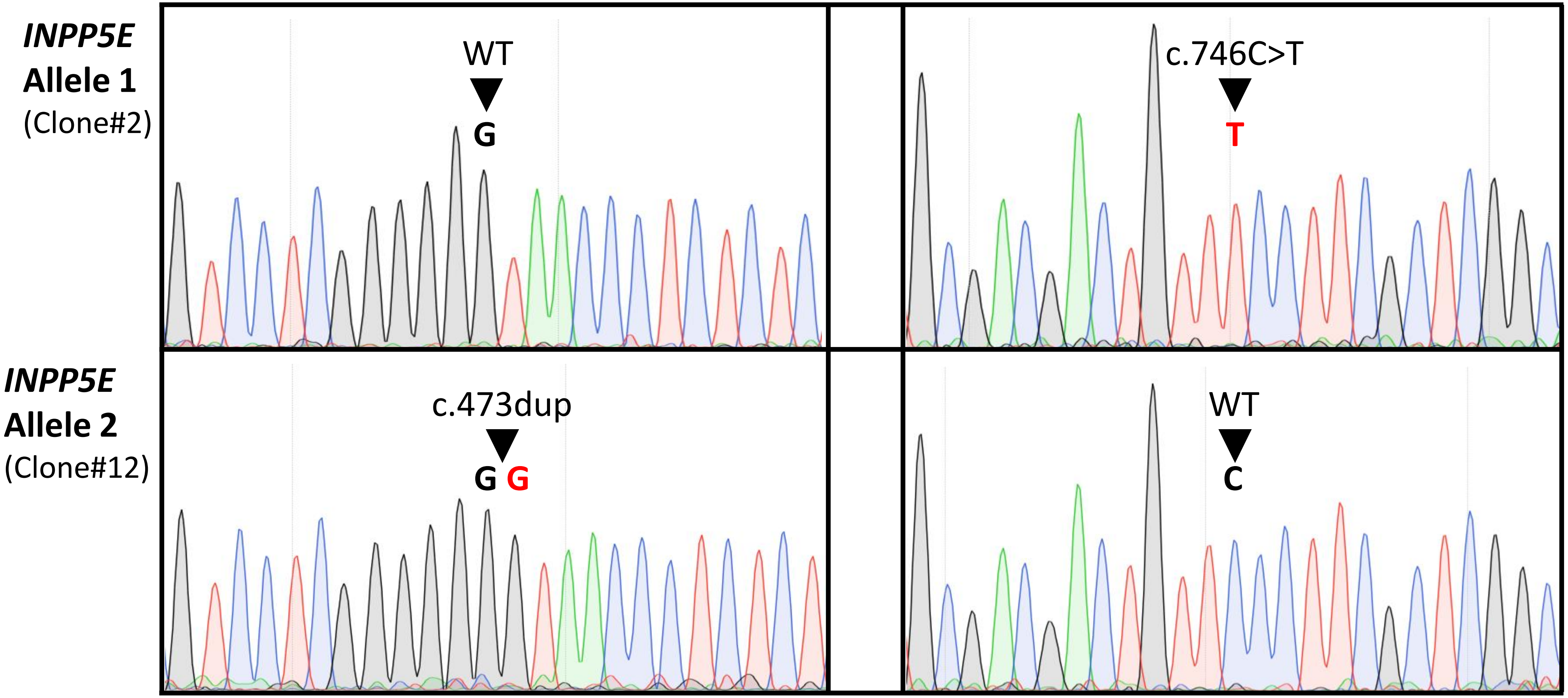

Supplementary Fig. 5 Variant validation and phasing in proband F.II-1

Supplementary Table 1_List of all *INPP5E* variants reported in the literature

INPP5E_g. (hg38)	*INPP5E* _c. (NM_019892.5)	INPP5E_p. (NP_063945.2)	Associated phenotype(s)	CADD Phred score	SuSPect_ Score [0-100]	SIFT_ Score [1-0]	Polyphen_ Score [0-1]	NetPhos score (0-1) / Kinase	Position-specific scoring matrix change	Missense3D	Allele Count (all reported cases)	# Homo	Reference	PMID
chr9:g.136438947dup	c.473dup	p.(Asn159*)	IRD (novel variant)	22.0	n.a.	n.a.	n.a.	n.a.	n.a.	n.a. (stop codon variant)	1	0	This study	n.a.
chr9:g.136438947del	c.473del	p.(Gly158Valfs*40)	JBTS	21.5	n.a.	n.a.	n.a.	n.a.	n.a.	n.a. (stop codon variant)	1	0	Brooks_2018	30055837
chr9:g.136438720dup	c.700dup	p.(Leu234Profs*56)	JBTS	32.0	n.a.	n.a.	n.a.	n.a.	n.a.	n.a. (stop codon variant)	1	0	Tsurusaki_2013	23034536
chr9:g.136438674G>A	c.[**746C>T**; 1787G>C][$]	p.[(**Ser249Phe**); (Arg596Thr)][$]	IRD (novel variant)	29.2	21	0.01	0.999	**0.84 / PKC**	n.a.	n.a. (cannot match position 249 to PDB)	3	1	This study	n.a.
chr9:g.136434832C>T	c.844G>A	p.(Gly282Arg)	IRD (overlooked phenotype expansion)	24.7	12	0.05	0.997	n.a.	n.a.	No structural damage detected	1	0	Birtel_2018	29555955
chr9:g.136434820C>T	c.856G>A	p.(Gly286Arg)	JBTS	24.7	15	0.02	1	n.a.	n.a.	Buried Gly replaced; Buried / exposed switch; Gly in a bend	4	2	Travaglini_2013; Kroes_2016	23386033; 25920555
chr9:g.136434802G>C	c.874C>G	p.(Arg292Gly)	IRD (overlooked phenotype expansion)	24.7	16	0.01	0.889	n.a.	Unfavourable	No structural damage detected	1	0	Stone_2017	28559085
chr9:g.136434769C>T	c.907G>A	p.(Val303Met)	JBTS	23.1	56	0.02	0.981	n.a.	Unfavourable	No structural damage detected	2	0	Travaglini_2013; Toma_2018	23386033; 29987673
chr9:g.136434762G>A	c.914C>T	p.(Thr305Ile)	IRD (novel variant)	27.1	94	0.01	1	0.47 / CaM-II	Unfavourable	Buried H-bond breakage	1	0	This study	n.a.
chr9:g.136434127G>A	c.944C>T	p.(Pro315Leu)	JBTS/JSRD	25.9	19	0.45	0.99	n.a.	Unfavourable	Buried / exposed switch	1	0	Stone_2017	28559085
chr9:g.136433279C>G	c.1035G>C	p.(Arg345Ser)	JBTS/JSRD	32.0	9	0.45	0.949	n.a.	n.a.	No structural damage detected	2	1	Travaglini_2013	23386033
chr9:g.136433250G>A	c.1064C>T	p.(Thr355Met)	JBTS	28.7	23	0.07	1	0.454 / GSK	n.a.	No structural damage detected	2	1	Radha Rama Devi_2020	32139166
chr9:g.136433241G>A	c.1073C>T	p.(Pro358Leu)	IRD (overlooked phenotype expansion)	29.4	23	0.03	1	n.a.	n.a.	No structural damage detected	1	0	Xu_2015	25999675
chr9:g.136433220G>A	c.1094C>T	p.(Ser365Leu)	IRD (novel variant)	24.5	48	0.14	1	0.47 / cdc2	Unfavourable	No structural damage detected	1	0	This study	n.a.
chr9:g.136433182G>A	c.1132C>T	p.(Arg378Cys)	JBTS	27.3	72	0	1	n.a.	Unfavourable	No structural damage detected	4	2	Bielas_2009	19668216
chr9:g.136432958G>T	c.1277C>A	p.(Thr426Asn)	JBTS	25.5	40	0.02	0.994	0.51 / CKI	n.a.	No structural damage detected	2	1	Travaglini_2013	23386033

chr9:g.136432563G>C	c.1303C>G	p.(Arg435Gly)	JBTS	25.4	88	0	1	n.a.	Unfavourable	Buried charge replaced; Buried H-bond breakage	2	1	Sonmez_2014	25818971
chr9:g.136432563G>A	c.1303C>T	p.(Arg435Trp)	JBTS	25.5	92	0	1	n.a.	Unfavourable	Clash; Buried charge replaced; Buried H-bond breakage	2	1	Shetty_2017	29230161
chr9:g.136432562C>T	c.1304G>A	p.(Arg435Gln)	JBTS	28.2	72	0	1	n.a.	Unfavourable	Buried charge replaced	8	3	Bielas_2009; Travaglini_2013; Kroes_2016; Suzuki_2016; Brooks_2018	19668216; 23386033; 25920555; 27434533; 30055837
chr9:g.136431980C>T	c.1393G>A	p.(Val465Ile)	IRD (novel variant)	23.4	14	0.26	0.611	n.a.	n.a.	No structural damage detected	2	1	This study	n.a.
chr9:g.136431971G>A	c.1402C>T	p.(Arg468Cys)	IRD (novel variant)	32	17	0.05	0.998	n.a.	n.a.	No structural damage detected	1	0	This study	n.a.
chr9:g.136431953A>G	c.1420T>C[#]	p.(Trp474Arg)	JBTS	32	63	0	1	n.a.	Unfavourable	Buried hydropilic introduced; Buried charge introduced; Buried H-bond breakage	1	0	Travaglini_2013	23386033
chr9:g.136431947C>T	c.1426G>A	p.(Gly476Arg)	JBTS	33	65	0	1	n.a.	Unfavourable	Clash; Buried charge introduced; Buried Gly repleaced	1	0	Suzuki_2016	27434533
chr9:g.136431917G>A	c.1456C>T	p.(Arg486Cys)	IRD (novel variant)	26.6	24	0.01	1	n.a.	n.a.	No structural damage detected	2	0	This study	n.a.
chr9:g.136431839G>A	c.[**1534C>T**; 1543C>T][¥]	p.**[(Arg512Trp)**; (Arg515Trp)][¥]	JBTS	19.7	34	0.02	0.998	n.a.	Favourable	No structural damage detected, No structural damage detected	4	2	Bielas_2009	19668216
chr9:g.136431830G>A	c.1543C>T[¥]	p.(Arg515Trp)[¥]	IRD (overlooked phenotype expansion)	20.1	58	0.02	0.996	n.a.	Unfavourable	No structural damage detected	1	0	Stone_2017	28559085
chr9:g.136431102C>G	c.1565G>C	p.(Gly522Ala)	JBTS	26.5	83	0.03	0.998	n.a.	Unfavourable	n.a. (cannot match position 552 to PDB)	4	2	Hardee_2017; Brooks_2018	29052317; 30055837
chr9:g.136431090G>A	c.1577C>T	p.(Pro526Leu)	IRD (novel variant)	26.9	39	0.07	0.976	n.a.	Unfavourable	No structural damage detected	1	0	This study	n.a.
chr9:g.136431067A>C	c.1600T>G	p.(Tyr534Asp)	JBTS	27.5	77	0.02	1	0.44 / INSR	Unfavourable	Buried charge introduced	1	0	Travaglini_2013	23386033
chr9:g.136431038G>C	c.1629C>G	p.(Tyr543*)	JBTS	35	n.a.	n.a.	n.a.	n.a.	n.a.	n.a. (stop codon variant)	2	1	Travaglini_2013	23386033
chr9:g.136431038G>T	c.1629C>A	p.(Tyr543*)	IRD (overlooked phenotype expansion)	35	n.a.	n.a.	n.a.	n.a.	n.a.	n.a. (stop codon variant)	1	0	Birtel_2018	29555955

chr9:g.136430410G>A	c.1669C>T	p.(Arg557Cys)	IRD (overlooked phenotype expansion)	29.2	80	0	1	n.a.	Unfavourable	Buried charge replaced; Buried salt bridge breakage	1	0	Xu_2015	25999675
chr9:g.136430409C>T	c.1670G>A	p.(Arg557His)	IRD (novel variant)	25.2	59	0	1	n.a.	Unfavourable	No structural damage detected	1	0	This study	n.a.
chr9:g.136430391C>T	c.1688G>A	p.(Arg563His)	JBTS	31	23	0	1	n.a.	n.a.	Buried H-bond breakage; Buried / exposed switch	4	2	Bielas_2009; Travaglini_2013	19668216; 23386033
chr9:g.136430341T>C	c.1738A>G	p.(Lys580Glu)	JBTS	23.6	34	0.26	0.3	n.a.	Unfavourable	No structural damage detected	2	1	Bielas_2009	19668216
chr9:g.136430326G>A	c.1753C>T	p.(Arg585Cys)	JBTS, IRD (overlooked phenotype expansion)	30	84	0	1	n.a.	Unfavourable	Buried H-bond breakage	3	0	Travaglini_2013; Toma_2018; Stone_2017	23386033; 29987673; 28559085
chr9:g.136430325C>T	c.1754G>A	p.(Arg585His)	IRD (novel variant)	28.4	67	0.01	1	n.a.	Unfavourable	No structural damage detected	2	0	This study	n.a.
chr9:g.136430319del	c.1760del	p.(Val587Glyfs*7)	IRD (novel variant)	33	n.a.	n.a.	n.a.	n.a.	n.a.	n.a. (stop codon variant)	1	0	This study	n.a.
chr9:g.136430316T>C	c.1763A>G	p.(Tyr588Cys)	JBTS	24.9	49	0.01	0.985	0.42 / SRC	n.a.	Buried H-bond breakage	2	1	de Goede_2016	26748598
chr9:g.136430305G>C	c.1774C>G	p.(Arg592Gly)	JBTS	25.1	36	0.08	0.688	n.a.	Unfavourable	No structural damage detected	1	0	Stone_2017	28559085
chr9:g.136430292_ 136430295del	c.1784_1787del	p.(Val595Glyfs*21)	JBTS	34	n.a.	n.a.	n.a.	n.a.	n.a.	n.a. (stop codon variant)	1	0	Brooks_2018	30055837
chr9:g.136430292C>G	c.[746C>T; **1787G>C**][$]	p.[(Ser249Phe); **(Arg596Thr)**][$]	IRD (novel variant)	25.9	29	0.03	0.881	n.a.	Unfavourable	No structural damage detected	3	1	This study	n.a.
chr9:g.136430279G>C	c.1800C>G	p.(Asp600Glu)	IRD (novel variant)	24.6	23	0.77	0.054	n.a.	n.a.	No structural damage detected	1	0	This study	n.a.
chr9:g.136429749G>A	c.1861C>T	p.(Arg621Trp)	IRD (overlooked phenotype expansion)	29.8	22	0.01	1	n.a.	Unfavourable	No structural damage detected	3	1	Wang_2013; this study	23847139; n.a.
chr9:g.136429748C>T	c.1862G>A	p.(Arg621Gln)	JBTS, IRD (overlooked phenotype expansion)	27.0	39	0.09	1	n.a.	Unfavourable	No structural damage detected	12	4	Tsurusaki_2013; Travaglini_2013; Stone_2017; Brooks_2018; Radha Rama Devi_2020; this study	23034536; 23386033; 28559085; 30055837; 32139166; n.a.
chr9:g.136429731G>A	c.1879C>T	p.(Gln627*)	MORM	44.0	n.a.	n.a.	n.a.	n.a.	n.a.	n.a. (stop codon variant)	4	2	Jacoby_2009; Khan_2019	19668215; 31173343
chr9:g.136429689A>G	c.1921T>C	p.(Cys641Arg)	JBTS	25.3	73	0.31	1	n.a.	Unfavourable	n.a. (cannot match position 641 to PDB)	4	2	Travaglini_2013	23386033

Variants belonging to the same complex alleles were indicated by symbols (¥ or $) and described separately (described variant in bold). IRD, inherited retinal degeneration; JBTS, Joubert Syndrome; (# Homo), number of homozygotes; n.a., not applicable; # : The INPP5E variant was found as a single hit in a mono-allelic JBTS case
The NetPhos 3.1 server predicts serine, threonine or tyrosine phosphorylation sites in eukaryotic proteins, and predictions are considered significant for scores >0.5
Variant deleteriousness is indicated by either low SIFT score or high Polyphen score

Supplementary Table 2: Genes targeted by the Genetic Eye Disease (GEDi) panel v6

Ensembl_ID	Gene name	Alternative gene name	Description
ENSG00000198691	*ABCA4*		ATP binding cassette subfamily A member 4
ENSG00000091262	*ABCC6*		ATP binding cassette subfamily C member 6
ENSG00000100997	*ABHD12*		abhydrolase domain containing 12
ENSG00000107897	*ACBD5*		acyl-CoA binding domain containing 5
ENSG00000100412	*ACO2*		aconitase 2
ENSG00000168615	*ADAM9*		ADAM metallopeptidase domain 9
ENSG00000140873	*ADAMTS18*		ADAM metallopeptidase with thrombospondin type 1 motif 18
ENSG00000152990	*ADGRA3*	*GPR125*	adhesion G protein-coupled receptor A3
ENSG00000164199	*ADGRV1*	*GPR98*	adhesion G protein-coupled receptor V1
ENSG00000135541	*AHI1*		Abelson helper integration site 1
ENSG00000129221	*AIPL1*		aryl hydrocarbon receptor interacting protein like 1
ENSG00000116127	*ALMS1*		ALMS1 centrosome and basal body associated protein
ENSG00000169379	*ARL13B*		ADP ribosylation factor like GTPase 13B
ENSG00000102931	*ARL2BP*		ADP ribosylation factor like GTPase 2 binding protein
ENSG00000113966	*ARL6*		ADP ribosylation factor like GTPase 6
ENSG00000162174	*ASRGL1*		asparaginase like 1
ENSG00000118217	*ATF6*		activating transcription factor 6
ENSG00000163635	*ATXN7*		ataxin 7
ENSG00000214413	*BBIP1*		BBSome interacting protein 1
ENSG00000174483	*BBS1*		Bardet-Biedl syndrome 1
ENSG00000179941	*BBS10*		Bardet-Biedl syndrome 10
ENSG00000181004	*BBS12*		Bardet-Biedl syndrome 12
ENSG00000125124	*BBS2*		Bardet-Biedl syndrome 2
ENSG00000140463	*BBS4*		Bardet-Biedl syndrome 4
ENSG00000163093	*BBS5*		Bardet-Biedl syndrome 5
ENSG00000138686	*BBS7*		Bardet-Biedl syndrome 7
ENSG00000122507	*BBS9*		Bardet-Biedl syndrome 9
ENSG00000167995	*BEST1*		bestrophin 1
ENSG00000223953	*C1QTNF5*		C1q and TNF related 5
ENSG00000160226	*C21orf2*		chromosome 21 open reading frame 2
ENSG00000179270	*C2orf71*		chromosome 2 open reading frame 71

ENSG00000197603	*C5orf42*		chromosome 5 open reading frame 42
ENSG00000156172	*C8orf37*		chromosome 8 open reading frame 37
ENSG00000167434	*CA4*		carbonic anhydrase 4
ENSG00000175544	*CABP4*		calcium binding protein 4
ENSG00000102001	*CACNA1F*		calcium voltage-gated channel subunit alpha1 F
ENSG00000151062	*CACNA2D4*		calcium voltage-gated channel auxiliary subunit alpha2delta 4
ENSG00000149260	*CAPN5*		calpain 5
ENSG00000048342	*CC2D2A*		coiled-coil and C2 domain containing 2A
ENSG00000107736	*CDH23*		cadherin related 23
ENSG00000062038	*CDH3*		cadherin 3
ENSG00000148600	*CDHR1*		cadherin related family member 1
ENSG00000110274	*CEP164*		centrosomal protein 164
ENSG00000198707	*CEP290*		centrosomal protein 290
ENSG00000106477	*CEP41*		centrosomal protein 41
ENSG00000148019	*CEP78*		centrosomal protein 78
ENSG00000173588	*CEP83*	*CCDC41*	centrosomal protein 83
ENSG00000188452	*CERKL*		ceramide kinase like
ENSG00000188419	*CHM*		CHM Rab escort protein 1
ENSG00000136425	*CIB2*		calcium and integrin binding family member 2
ENSG00000188603	*CLN3*		CLN3 battenin
ENSG00000102805	*CLN5*		CLN5 intracellular trafficking protein
ENSG00000128973	*CLN6*		CLN6 transmembrane ER protein
ENSG00000182372	*CLN8*		CLN8 transmembrane ER and ERGIC protein
ENSG00000163646	*CLRN1*		clarin 1
ENSG00000198515	*CNGA1*		cyclic nucleotide gated channel alpha 1
ENSG00000144191	*CNGA3*		cyclic nucleotide gated channel alpha 3
ENSG00000070729	*CNGB1*		cyclic nucleotide gated channel beta 1
ENSG00000170289	*CNGB3*		cyclic nucleotide gated channel beta 3
ENSG00000158158	*CNNM4*		cyclin and CBS domain divalent metal cation transport mediator 4
ENSG00000060718	*COL11A1*		collagen type XI alpha 1 chain
ENSG00000139219	*COL2A1*		collagen type II alpha 1 chain
ENSG00000112280	*COL9A1*		collagen type IX alpha 1 chain
ENSG00000134376	*CRB1*		crumbs 1 cell polarity complex component
ENSG00000105392	*CRX*		cone-rod homeobox

ENSG00000104218	*CSPP1*		centrosome and spindle pole associated protein 1
ENSG00000145476	*CYP4V2*		cytochrome P450 family 4 subfamily V member 2
ENSG00000117682	*DHDDS*		dehydrodolichyl diphosphate synthase subunit
ENSG00000140829	*DHX38*		DEAH-box helicase 38
ENSG00000156171	*DRAM2*		DNA damage regulated autophagy modulator 2
ENSG00000197057	*DTHD1*		death domain containing 1
ENSG00000115380	*EFEMP1*		EGF containing fibulin like extracellular matrix protein 1
ENSG00000118402	*ELOVL4*		ELOVL fatty acid elongase 4
ENSG00000127463	*EMC1*		ER membrane protein complex subunit 1
ENSG00000225830	*ERCC6*		ERCC excision repair 6 chromatin remodeling factor
ENSG00000188107	*EYS*		eyes shut homolog (Drosophila)
ENSG00000170264	*FAM161A*		family with sequence similarity 161 member A
ENSG00000162769	*FLVCR1*		feline leukemia virus subgroup C cellular receptor 1
ENSG00000174804	*FZD4*		frizzled class receptor 4
ENSG00000156466	*GDF6*		growth differentiation factor 6
ENSG00000114349	*GNAT1*		G protein subunit alpha transducin 1
ENSG00000134183	*GNAT2*		G protein subunit alpha transducin 2
ENSG00000111664	*GNB3*		G protein subunit beta 3
ENSG00000090581	*GNPTG*		N-acetylglucosamine-1-phosphate transferase gamma subunit
ENSG00000101850	*GPR143*		G protein-coupled receptor 143
ENSG00000188888	*GPR179*		G protein-coupled receptor 179
ENSG00000185974	*GRK1*		G protein-coupled receptor kinase 1
ENSG00000113262	*GRM6*		glutamate metabotropic receptor 6
ENSG00000030582	*GRN*		granulin precursor
ENSG00000048545	*GUCA1A*		guanylate cyclase activator 1A
ENSG00000132518	*GUCY2D*		guanylate cyclase 2D retinal
ENSG00000170445	*HARS*		histidyl-tRNA synthetase
ENSG00000112855	*HARS2*		histidyl-tRNA synthetase 2 mitochondrial
ENSG00000068024	*HDAC4*		histone deacetylase 4
ENSG00000215612	*HMX1*		H6 family homeobox 1
ENSG00000101365	*IDH3B*		isocitrate dehydrogenase 3 (NAD(+)) beta
ENSG00000163913	*IFT122*		intraflagellar transport 122
ENSG00000187535	*IFT140*		intraflagellar transport 140
ENSG00000138002	*IFT172*		intraflagellar transport 172

ENSG00000100360	*IFT27*		intraflagellar transport 27
ENSG00000119650	*IFT43*		intraflagellar transport 43
ENSG00000068885	*IFT80*		intraflagellar transport 80
ENSG00000032742	*IFT88*		intraflagellar transport 88
ENSG00000073009	*IKBKG*		inhibitor of nuclear factor kappa B kinase subunit gamma
ENSG00000106348	*IMPDH1*		inosine monophosphate dehydrogenase 1
ENSG00000112706	*IMPG1*		interphotoreceptor matrix proteoglycan 1
ENSG00000081148	*IMPG2*		interphotoreceptor matrix proteoglycan 2
ENSG00000148384	*INPP5E*		inositol polyphosphate-5-phosphatase E
ENSG00000119509	*INVS*		inversin
ENSG00000173226	*IQCB1*		IQ motif containing B1
ENSG00000136156	*ITM2B*		integral membrane protein 2B
ENSG00000101384	*JAG1*		jagged canonical Notch ligand 1
ENSG00000115474	*KCNJ13*		potassium voltage-gated channel subfamily J member 13
ENSG00000168263	*KCNV2*		potassium voltage-gated channel modifier subfamily V member 2
ENSG00000243335	*KCTD7*		potassium channel tetramerization domain containing 7
ENSG00000122778	*KIAA1549*		KIAA1549
ENSG00000138160	*KIF11*		kinesin family member 11
ENSG00000088970	*KIZ*	*PLK1S1*	kizuna centrosomal protein
ENSG00000122550	*KLHL7*		kelch like family member 7
ENSG00000135338	*LCA5*		LCA5 lebercilin
ENSG00000121207	*LRAT*		lecithin retinol acyltransferase (phosphatidylcholine--retinol O-acyltransferase)
ENSG00000183423	*LRIT3*		leucine rich repeat Ig-like and transmembrane domains 3
ENSG00000162337	*LRP5*		LDL receptor related protein 5
ENSG00000163818	*LZTFL1*		leucine zipper transcription factor like 1
ENSG00000111837	*MAK*		male germ cell associated kinase
ENSG00000114738	*MAPKAPK3*		mitogen-activated protein kinase-activated protein kinase 3
ENSG00000153208	*MERTK*		MER proto-oncogene tyrosine kinase
ENSG00000116688	*MFN2*		mitofusin 2
ENSG00000235718	*MFRP*		membrane frizzled-related protein
ENSG00000164073	*MFSD8*		major facilitator superfamily domain containing 8
ENSG00000125863	*MKKS*		McKusick-Kaufman syndrome
ENSG00000011143	*MKS1*		Meckel syndrome type 1
ENSG00000198899	*MT-ATP6*		mitochondrially encoded ATP synthase 6

ENSG00000228253	*MT-ATP8*		mitochondrially encoded ATP synthase 8
ENSG00000198804	*MT-CO1*		mitochondrially encoded cytochrome c oxidase I
ENSG00000198712	*MT-CO2*		mitochondrially encoded cytochrome c oxidase II
ENSG00000198938	*MT-CO3*		mitochondrially encoded cytochrome c oxidase III
ENSG00000198727	*MT-CYB*		mitochondrially encoded cytochrome b
ENSG00000198888	*MT-ND1*		mitochondrially encoded NADH:ubiquinone oxidoreductase core subunit 1
ENSG00000198763	*MT-ND2*		mitochondrially encoded NADH:ubiquinone oxidoreductase core subunit 2
ENSG00000198840	*MT-ND3*		mitochondrially encoded NADH:ubiquinone oxidoreductase core subunit 3
ENSG00000198886	*MT-ND4*		mitochondrially encoded NADH:ubiquinone oxidoreductase core subunit 4
ENSG00000212907	*MT-ND4L*		mitochondrially encoded NADH:ubiquinone oxidoreductase core subunit 4L
ENSG00000198786	*MT-ND5*		mitochondrially encoded NADH:ubiquinone oxidoreductase core subunit 5
ENSG00000198695	*MT-ND6*		mitochondrially encoded NADH:ubiquinone oxidoreductase core subunit 6
ENSG00000210176	*MT-TH*		mitochondrially encoded tRNA histidine
ENSG00000209082	*MT-TL1*		mitochondrially encoded tRNA leucine 1 (UUA/G)
ENSG00000210196	*MT-TP*		mitochondrially encoded tRNA proline
ENSG00000210184	*MT-TS2*		mitochondrially encoded tRNA serine 2 (AGU/C)
ENSG00000138823	*MTTP*		microsomal triglyceride transfer protein
ENSG00000110921	*MVK*		mevalonate kinase
ENSG00000137474	*MYO7A*		myosin VIIA
ENSG00000124479	*NDP*		NDP norrin cystine knot growth factor
ENSG00000117650	*NEK2*		NIMA related kinase 2
ENSG00000162992	*NEUROD1*		neuronal differentiation 1
ENSG00000173614	*NMNAT1*		nicotinamide nucleotide adenylyltransferase 1
ENSG00000144061	*NPHP1*		nephrocystin 1
ENSG00000113971	*NPHP3*		nephrocystin 3
ENSG00000131697	*NPHP4*		nephrocystin 4
ENSG00000126368	*NR1D1*		nuclear receptor subfamily 1 group D member 1
ENSG00000031544	*NR2E3*		nuclear receptor subfamily 2 group E member 3
ENSG00000129535	*NRL*		neural retina leucine zipper
ENSG00000171773	*NXNL1*		nucleoredoxin like 1
ENSG00000188937	*NYX*		nyctalopin
ENSG00000065154	*OAT*		ornithine aminotransferase
ENSG00000104044	*OCA2*		OCA2 melanosomal transmembrane protein
ENSG00000046651	*OFD1*		OFD1 centriole and centriolar satellite protein

ENSG00000198836	*OPA1*		OPA1 mitochondrial dynamin like GTPase
ENSG00000125741	*OPA3*		OPA3 outer mitochondrial membrane lipid metabolism regulator
ENSG00000102076	*OPN1LW*		opsin 1 long wave sensitive
ENSG00000147380	*OPN1MW*		opsin 1 medium wave sensitive
ENSG00000128617	*OPN1SW*		opsin 1 short wave sensitive
ENSG00000165588	*OTX2*		orthodenticle homeobox 2
ENSG00000125779	*PANK2*		pantothenate kinase 2
ENSG00000075891	*PAX2*		paired box 2
ENSG00000150275	*PCDH15*		protocadherin related 15
ENSG00000161217	*PCYT1A*		phosphate cytidylyltransferase 1 choline alpha
ENSG00000132915	*PDE6A*		phosphodiesterase 6A
ENSG00000133256	*PDE6B*		phosphodiesterase 6B
ENSG00000095464	*PDE6C*		phosphodiesterase 6C
ENSG00000156973	*PDE6D*		phosphodiesterase 6D
ENSG00000185527	*PDE6G*		phosphodiesterase 6G
ENSG00000139053	*PDE6H*		phosphodiesterase 6H
ENSG00000186862	*PDZD7*		PDZ domain containing 7
ENSG00000127980	*PEX1*		peroxisomal biogenesis factor 1
ENSG00000157911	*PEX10*		peroxisomal biogenesis factor 10
ENSG00000142655	*PEX14*		peroxisomal biogenesis factor 14
ENSG00000121680	*PEX16*		peroxisomal biogenesis factor 16
ENSG00000162735	*PEX19*		peroxisomal biogenesis factor 19
ENSG00000164751	*PEX2*		peroxisomal biogenesis factor 2
ENSG00000139197	*PEX5*		peroxisomal biogenesis factor 5
ENSG00000124587	*PEX6*		peroxisomal biogenesis factor 6
ENSG00000112357	*PEX7*		peroxisomal biogenesis factor 7
ENSG00000107537	*PHYH*		phytanoyl-CoA 2-hydroxylase
ENSG00000127472	*PLA2G5*		phospholipase A2 group V
ENSG00000032444	*PNPLA6*		patatin like phospholipase domain containing 6
ENSG00000139323	*POC1B*		POC1 centriolar protein B
ENSG00000131238	*PPT1*		palmitoyl-protein thioesterase 1
ENSG00000214140	*PRCD*		photoreceptor disc component
ENSG00000112238	*PRDM13*		PR/SET domain 13
ENSG00000007062	*PROM1*		prominin 1

ENSG00000117360	*PRPF3*		pre-mRNA processing factor 3
ENSG00000105618	*PRPF31*		pre-mRNA processing factor 31
ENSG00000136875	*PRPF4*		pre-mRNA processing factor 4
ENSG00000101161	*PRPF6*		pre-mRNA processing factor 6
ENSG00000174231	*PRPF8*		pre-mRNA processing factor 8
ENSG00000112619	*PRPH2*		peripherin 2
ENSG00000157869	*RAB28*		RAB28 member RAS oncogene family
ENSG00000173976	*RAX2*		retina and anterior neural fold homeobox 2
ENSG00000107618	*RBP3*		retinol binding protein 3
ENSG00000138207	*RBP4*		retinol binding protein 4
ENSG00000136144	*RCBTB1*		RCC1 and BTB domain containing protein 1
ENSG00000198570	*RD3*		retinal degeneration 3
ENSG00000139988	*RDH12*		retinol dehydrogenase 12 (all-trans/9-cis/11-cis)
ENSG00000135437	*RDH5*		retinol dehydrogenase 5
ENSG00000115255	*REEP6*		receptor accessory protein 6
ENSG00000148604	*RGR*		retinal G protein coupled receptor
ENSG00000108370	*RGS9*		regulator of G protein signaling 9
ENSG00000186326	*RGS9BP*		regulator of G protein signaling 9 binding protein
ENSG00000163914	*RHO*		rhodopsin
ENSG00000140522	*RLBP1*		retinaldehyde binding protein 1
ENSG00000104237	*RP1*		RP1 axonemal microtubule associated
ENSG00000183638	*RP1L1*		RP1 like 1
ENSG00000102218	*RP2*		RP2 ARL3 GTPase activating protein
ENSG00000164610	*RP9*		RP9 pre-mRNA splicing factor
ENSG00000116745	*RPE65*		RPE65 retinoid isomerohydrolase
ENSG00000156313	*RPGR*		retinitis pigmentosa GTPase regulator
ENSG00000092200	*RPGRIP1*		retinitis pigmentosa GTPase regulator interacting protein 1
ENSG00000103494	*RPGRIP1L*		RPGRIP1 like
ENSG00000102104	*RS1*		retinoschisin 1
ENSG00000130561	*SAG*		S-antigen visual arrestin
ENSG00000054282	*SDCCAG8*		serologically defined colon cancer antigen 8
ENSG00000196189	*SEMA4A*		semaphorin 4A
ENSG00000074621	*SLC24A1*		solute carrier family 24 member 1
ENSG00000164209	*SLC25A46*		solute carrier family 25 member 46

ENSG00000164175	*SLC45A2*		solute carrier family 45 member 2
ENSG00000013293	*SLC7A14*		solute carrier family 7 member 14
ENSG00000144028	*SNRNP200*		small nuclear ribonucleoprotein U5 subunit 200
ENSG00000042317	*SPATA7*		spermatogenesis associated 7
ENSG00000072080	*SPP2*		secreted phosphoprotein 2
ENSG00000128039	*SRD5A3*		steroid 5 alpha-reductase 3
ENSG00000187079	*TEAD1*		TEA domain transcription factor 1
ENSG00000126953	*TIMM8A*		translocase of inner mitochondrial membrane 8A
ENSG00000100234	*TIMP3*		TIMP metallopeptidase inhibitor 3
ENSG00000171202	*TMEM126A*		transmembrane protein 126A
ENSG00000205084	*TMEM231*		transmembrane protein 231
ENSG00000155755	*TMEM237*		transmembrane protein 237
ENSG00000164953	*TMEM67*		transmembrane protein 67
ENSG00000197579	*TOPORS*		TOP1 binding arginine/serine rich protein
ENSG00000166340	*TPP1*	*TPPA*	tripeptidyl peptidase 1
ENSG00000213689	*TREX1*		three prime repair exonuclease 1
ENSG00000119401	*TRIM32*		tripartite motif containing 32
ENSG00000134160	*TRPM1*		transient receptor potential cation channel subfamily M member 1
ENSG00000106025	*TSPAN12*		tetraspanin 12
ENSG00000123607	*TTC21B*		tetratricopeptide repeat domain 21B
ENSG00000100154	*TTC28*		tetratricopeptide repeat domain 28
ENSG00000165533	*TTC8*		tetratricopeptide repeat domain 8
ENSG00000119685	*TTLL5*		tubulin tyrosine ligase like 5
ENSG00000137561	*TTPA*		alpha tocopherol transfer protein
ENSG00000166402	*TUB*		tubby bipartite transcription factor
ENSG00000137822	*TUBGCP4*		tubulin gamma complex associated protein 4
ENSG00000112041	*TULP1*		tubby like protein 1
ENSG00000077498	*TYR*		tyrosinase
ENSG00000107165	*TYRP1*		tyrosinase related protein 1
ENSG00000006611	*USH1C*		USH1 protein network component harmonin
ENSG00000182040	*USH1G*		USH1 protein network component sans
ENSG00000042781	*USH2A*		usherin
ENSG00000038427	*VCAN*		versican
ENSG00000132549	*VPS13B*		vacuolar protein sorting 13 homolog B

ENSG00000143951	*WDPCP*		WD repeat containing planar cell polarity effector
ENSG00000157796	*WDR19*		WD repeat domain 19
ENSG00000119333	*WDR34*		WD repeat domain 34
ENSG00000118965	*WDR35*		WD repeat domain 35
ENSG00000109501	*WFS1*		wolframin ER transmembrane glycoprotein
ENSG00000095397	*WHRN*	*DFNB31*	whirlin
ENSG00000175213	*ZNF408*		zinc finger protein 408
ENSG00000102935	*ZNF423*		zinc finger protein 423 (AD also mentioned in OMIM for Joubert…)
ENSG00000163795	*ZNF513*		zinc finger protein 513

Supplementary Table 3: Clinical characteristics of IRD probands carrying *INPP5E* variants

Family_ Proband	Proband_ Research ID (Sex, age at last exam)	Diagnosis (Onset age & symptoms)	Visual acuity	Goldmann perimetry	Fundus [d]	Autofluorescence (AF)	OCT	Electroretinogram [e]
A.II-1	OGI3559_5164 (M, 8 years)	LCA (infancy; nystagmus, visual inattention)	OD 20/100<br>OS 20/100	Severely constricted (V4e 30-40°); Inferior mid peripheral islands	Parafoveal atrophy; extramacular mottling; fine clumps of pigment	Parafoveal hyperAF	Foveal preservation of ONL; extrafoveal loss	2 months:<br>Scotopic response WNL<br>30 Hz flicker severely depressed<br>4 years:<br>All responses severely depressed
B.II-4	M0L0641-1 (F, 16 years) [a]	LCA (infancy; nystagmus, poor vision)	16 years:<br>OD 20/120<br>OS 20/100	Severely constricted (IV < 10°)	Parafoveal atrophy; macular staphyloma; midperipheral atrophy and bone spicules	Parafoveal hyperAF ring; peripheral hypoAF	Fovea preserved; parafoveal loss of EZ	4.5 years:<br>Rods: ND<br>Cones: ND
C.II-1	LL135 (F, 20 years)	LCA (infancy; nystagmus)	OD 20/200<br>OS 20/200	12 years: Severely constricted (V4 ~50-60°)	Macula preserved; midperipheral atrophy and bone spicules	Not interpretable	Fovea preserved; peripheral EZ loss	14 years:<br>Rods: ND<br>Cones: ND
D.II-4	LL105 (F, 55 years) [a]	LCA (infancy; nystagmus, oculodigital sign)	OD NLP<br>OS NLP	n/a	Macular and peripheral atrophy with bone spicules	n/a	Diffuse PR and RPE atrophy	n/a
E.II-4	OGI2307_3818 (F, 44 years) [a]	RCD (teens; nyctalopia)	OD 20/20<br>OS 20/20	Mildly constricted (I4e 30-40°; V4e full)	Midperipheral atrophy and bone spicules	Para- and perifoveal hyperAF rings; midperipheral hypoAF	EZ present throughout	24 years:<br>Dim scotopic flash ~20% of NL;<br>30 Hz flicker ~50% of NL<br>44 years:<br>Rods: ND<br>30 Hz flicker ~20% of NL

F.II-1	OGI1819_3159 (F, 16 years)	RCD (teens; nyctalopia)	OD 20/40 OS 20/60[b]	Mildly constricted (I2e 10°; I4e 70°; V4e full)	Macular staphyloma; foveal hypopigmentation; midperipheral atrophy and rare bone spicules	Foveal and parafoveal hyperAF; midperipheral hypoAF	Foveal EZ interruption; parafoveal EZ loss	Rods: ND<br>30 Hz flicker ~ 25% of NL
G.II-1	OGI2386_3945 (F, 50 years)	RCD (childhood; nyctalopia)	OD HM OS HM	n/a	Macular atrophy; midperipheral bone spicules	n/a	n/a	50 years:<br>Rods: ND<br>Cones: ND
H.III-1	LL235 (F, 55 years)	RCD (teens; nyctalopia, photophobia)	OD 20/50 OS 20/25	n/a	Macular edema; midperipheral atrophy and rare bone spicules	n/a	Cystoid macular edema OU; foveal EZ intact; peripheral EZ loss	56 years:<br>Rods: ND<br>Cones: ND
I.II-1	GC19652 (M, 22 years) [a]	RCD (childhood; nyctalopia)	OD 20/20 OS 20/20	Mildly constricted (details n/a)	Parafoveal atrophy; midperipheral atrophy and rare bone spicules	Foveal and parafoveal hyperAF, midperipheral hypoAF	Fovea preserved; parafoveal EZ loss	16 years:<br>Dim scotopic flash: ND<br>30 Hz flicker ~15-25% of normal
J.II-1	GC16358 (M, 41 years)	RCD (teens; dark adaptation)	OD 20/40 OS 20/30	n/a	Midperipheral bone spicules	Parafoveal hyperAF ring; midperipheral hypoAF with paravascular sparing	Fovea preserved; parafoveal EZ loss	n/a
K.II-1	GC22740 (F, 31 years) [c]	RCD (childhood; nyctalopia)	Age 23 OD 20/40, OS 20/400	n/a	Subtle macular pigment abnormalities; midperipheral atrophy and bone spicules	Perifoveal hyperAF ring; midperipheral hypoAF	Fovea preserved; parafoveal EZ loss	Rods: ND<br>30 Hz flicker: Reduced

L.II-1	TB315_R693<br>(M, 34 years) [a]	RCD<br>(23 years;<br>nyctalopia,<br>visual field<br>constriction)	OD 20/30<br>OS 20/40	n/a	Peripheral bone<br>spicules	n/a	n/a	Dim scotopic flash: ND<br>30 Hz flicker: ~ 20% of NL

Imaging and testing correspond to age from most recent visual acuity reported unless otherwise noted.
Abbreviations: AF – autofluorescence; EZ – ellipsoid zone; HM – hand motion visual acuity; LCA – Leber congenital amaurosis; n/a – not available; ND – nondetectable; NL – normal; NLP – no light perception; OD – right eye; ONL – outer nuclear layer; OS – left eye; OU – both eyes; PR – photoreceptor; RCD – rod-cone dystrophy; RPE – retinal pigment epithelium

[a] Proband has affected sibling not represented in this cohort

[b] Visual acuity limited by amblyopia

[c] Proband showed congenital oculomotor apraxia

[d] Disc pallor and vascular attenuation seen in all patients

[illegible]

Supplementary Table 4_Pathogenicity scores for the INPP5E variants summed by patient reported in this study and before

Allele1_c. (NM_019892.5)	Allele1_p. (NP_063945.2)	Allele2_c. (NM_019892.5)	Allele2_p. (NP_063945.2)	Ocular Phenotype*	Non-Neurological Organ Involvement	Syndromic Diagnosis	Reference	Combined CADD Phred-score for both alleles**	Combined SusPect score for both alleles***	Combined SIFT Score for both alleles	Combined Polyphen Score for both alleles	Number of Affected Individuals in the Family (inlcuding proband)
c.473dup	p.(Asn159*)	c.[746C>T; 1787G>C]	p.[(Ser249Phe); (Arg596Thr)]	IRD	None	None	this study	51.2	n.a.	n.a.	n.a.	1
c.[746C>T; 1787G>C]	p.[(Ser249Phe); (Arg596Thr)]	c.[746C>T; 1787G>C]	p.[(Ser249Phe); (Arg596Thr)]	IRD	None	None	this study	58.4	42	0.02	1.998	1
c.844G>A	p.(Gly282Arg)	c.1629C>A	p.(Tyr543*)	IRD	None	None	Birtel_2018	59.7	n.a.	n.a.	n.a.	1
c.874C>G	p.(Arg292Gly)	c.1753C>T	p.(Arg585Cys)	IRD	None	None	Stone_2017	54.7	100	0.01	1.889	1
c.914C>T	p.(Thr305Ile)	c.1456C>T	p.(Arg486Cys)	IRD	None	None	this study	53.7	118	0.02	2	1
c.1073C>T	p.(Pro358Leu)	c.1669C>T	p.(Arg557Cys)	IRD	None	None	Xu_2015	58.6	103	0.03	2	1
c.1094C>T	p.(Ser365Leu)	c.1800C>G	p.(Asp600Glu)	IRD	None	None	this study	49.1	71	0.91	1.054	1
c.1393G>A	p.(Val465Ile)	c.1393G>A	p.(Val465Ile)	IRD	None	None	this study	46.8	28	0.52	1.222	3
c.1402C>T	p.(Arg468Cys)	c.1861C>T	p.(Arg621Trp)	IRD	None	None	this study	61.8	39	0.06	1.998	2
c.1456C>T	p.(Arg486Cys)	c.1577C>T	p.(Pro526Leu)	IRD	None	None	this study	58.9	56	0.08	1.976	1
c.1543C>T	p.(Arg515Trp)	c.1862G>A	p.(Arg621Gln)	IRD	None	None	Stone_2017	47.1	97	0.11	1.996	1
c.1670G>A	p.(Arg557His)	c.1754G>A	p.(Arg585His)	IRD	None	None	this study	53.6	126	0.01	2	2
c.1754G>A	p.(Arg585His)	c.1760del	p.(Val587Glyfs*7)	IRD#	None	Some JBTS clinical features, not diagnosed as JBTS or MORM	this study	61.4	n.a.	n.a.	n.a.	1
c.1861C>T	p.(Arg621Trp)	c.1861C>T	p.(Arg621Trp)	IRD	None	None	Wang_2013	59.6	44	0.02	2	1
c.1862G>A	p.(Arg621Gln)	c.1862G>A	p.(Arg621Gln)	IRD	None	None	this study	54	78	0.18	2	2
c.1862G>A	p.(Arg621Gln)	c.1862G>A	p.(Arg621Gln)	IRD	None	None	this study	54	78	0.18	2	2
c.1862G>A	p.(Arg621Gln)	c.1862G>A	p.(Arg621Gln)	IRD#	None	Some JBTS clinical features, not diagnosed as JBTS or MORM	this study	54	78	0.18	2	1
c.473del	p.(Gly158Valfs*40)	c.1304G>A	p.(Arg435Gln)	Coloboma	Kidney	JBTS	Brooks_ 2018	49.7	n.a.	n.a.	n.a.	1
c.700dup	p.(Leu234Profs*56)	c.1862G>A	p.(Arg621Gln)	IRD	Craniofacial	JBTS	Tsurusaki_ 2013	59	n.a.	n.a.	n.a.	2

c.856G>A	p.(Gly286Arg)	c.856G>A	p.(Gly286Arg)	Coloboma	None	JBTS	Travaglini_ 2013	49.4	30	0.04	2	1
c.856G>A	p.(Gly286Arg)	c.856G>A	p.(Gly286Arg)	IRD	unknown	JBTS	Kroes_2016	49.4	30	0.04	2	1
c.907G>A	p.(Val303Met)	c.1753C>T	p.(Arg585Cys)	IRD in both and coloboma in 1 of 2 sibs	None reported	JBTS	Toma_2018	53.1	140	0.02	1.981	2
c.907G>A	p.(Val303Met)	c.1753C>T	p.(Arg585Cys)	IRD in both and coloboma in 1 of 2 sibs	None	JBTS	Travaglini_ 2013	53.1	140	0.02	1.981	2
c.944C>T	p.(Pro315Leu)	c.1774C>G	p.(Arg592Gly)	IRD	unknown	JBTS	Stone_2017	51	55	0.53	1.678	2
c.1035G>C	p.(Arg345Ser)	c.1035G>C	p.(Arg345Ser)	IRD	None	JBTS	Travaglini_ 2013	64	18	0.9	1.898	1
c.1064C>T	p.(Thr355Met)	c.1064C>T	p.(Thr355Met)	None	None	JBTS	Radha Rama Devi_2020	57.4	46	0.14	2	1
c.1132C>T	p.(Arg378Cys)	c.1132C>T	p.(Arg378Cys)	IRD	None	JBTS	Bielas_2009	54.6	144	0	2	2
c.1132C>T	p.(Arg378Cys)	c.1132C>T	p.(Arg378Cys)	IRD	Liver (elevated transaminases, fibrosis/cirrhosis)	JBTS	Bielas_2009	54.6	144	0	2	1
c.1277C>A	p.(Thr426Asn)	c.1277C>A	p.(Thr426Asn)	None	None	JBTS	Travaglini_ 2013	51	80	0.04	1.988	3
c.1303C>G	p.(Arg435Gly)	c.1303C>G	p.(Arg435Gly)	None	Kidney cysts, arterial hypertension, macrocephaly, post-axial polydactily	JBTS	Sonmez_ 2014	50.8	176	0	2	1
c.1303C>T	p.(Arg435Trp)	c.1303C>T	p.(Arg435Trp)	None	Kidney	JBTS	Shetty_2017	51	184	0	2	1
c.1304G>A	p.(Arg435Gln)	c.1304G>A	p.(Arg435Gln)	None	Kidney cysts; microcephaly	JBTS	Bielas_2009	56.4	144	0	2	2
c.1304G>A	p.(Arg435Gln)	c.1304G>A	p.(Arg435Gln)	IRD	None	JBTS	Travaglini_ 2013	56.4	144	0	2	1
c.1304G>A	p.(Arg435Gln)	c.1304G>A	p.(Arg435Gln)	Retinal dysplasia	Kidney (nephronophthisis); Skin (porokeratosis)	JBTS	Kroes_2016	56.4	144	0	2	1
c.1304G>A	p.(Arg435Gln)	c.1426G>A	p.(Gly476Arg)	None	Kidney; Liver	JBTS	Suzuki_2016	61.2	137	0	2	1
c.1534C>T; 1543C>T	p.[(Arg512Trp); (Arg515Trp)]	c.1534C>T; 1543C>T	p.[(Arg512Trp); (Arg515Trp)]	None	None	JBTS	Bielas_2009	40.2	68	0.04	1.996	1
c.1534C>T; 1543C>T	p.[(Arg512Trp); (Arg515Trp)]	c.1534C>T; 1543C>T	p.[(Arg512Trp); (Arg515Trp)]	IRD	None	JBTS	Bielas_2009	40.2	68	0.04	1.996	5
c.1600T>G	p.(Tyr534Asp)	c.1862G>A	p.(Arg621Gln)	None	Kidney (ectopia)	JBTS	Travaglini_ 2013	54.5	116	0.11	2	2

c.1629C>G	p.(Tyr543*)	c.1629C>G	p.(Tyr543*)	IRD and coloboma	None	JBTS	Travaglini_ 2013	70	n.a.	n.a.	n.a.	1
c.1565G>C	p.(Gly522Ala)	c.1565G>C	p.(Gly522Ala)	IRD	Facial features; short stature	JBTS	Hardee_201 7	53	166	0.06	1.996	2
c.1565G>C	p.(Gly522Ala)	c.1565G>C	p.(Gly522Ala)	IRD	None	JBTS	Brooks_ 2018	53	166	0.06	1.996	2
c.1688G>A	p.(Arg563His)	c.1688G>A	p.(Arg563His)	IRD	Plagiocephaly	JBTS	Bielas_2009	62	46	0	2	4
c.1688G>A	p.(Arg563His)	c.1688G>A	p.(Arg563His)	unknown	None	JBTS	Travaglini_ 2013	62	46	0	2	1
c.1738A>G	p.(Lys580Glu)	c.1738A>G	p.(Lys580Glu)	None	Liver (elevated transaminases and fibrosis)	JBTS	Bielas_2009	47.2	68	0.52	0.6	3
c.1763A>G	p.(Tyr588Cys)	c.1763A>G	p.(Tyr588Cys)	None	limb anomalies in 1; Craniofacial dysmorphic features in 4	JBTS	de Goede_ 2016	49.8	98	0.02	1.97	6
c.1784_1787del	p.(Val595Glyfs*21)	c.1862G>A	p.(Arg621Gln)	IRD	Kidney; Liver	JBTS	Brooks_ 2018	61	n.a.	n.a.	n.a.	1
c.1862G>A	p.(Arg621Gln)	c.1862G>A	p.(Arg621Gln)	None	None	JBTS	Radha Rama Devi_2020	54	78	0.18	2	1
c.1879C>T	p.(Gln627*)	c.1879C>T	p.(Gln627*)	IRD	Hipogonadism	MORM	Jacoby_ 2009	88	n.a.	n.a.	n.a.	14
c.1879C>T	p.(Gln627*)	c.1879C>T	p.(Gln627*)	IRD in 7 of 9 affected family members	Hipogonadism in 6; Renal impairment in 2	MORM	Khan_2019	88	n.a.	n.a.	n.a.	9
c.1921T>C	p.(Cys641Arg)	c.1921T>C	p.(Cys641Arg)	IRD and coloboma	None	JBTS	Travaglini_ 2013	50.6	146	0.62	2	2
c.1921T>C	p.(Cys641Arg)	c.1921T>C	p.(Cys641Arg)	IRD in 1 of 2 sibs	None	JBTS	Travaglini_ 2013	50.6	146	0.62	2	2

* If not specified, the phenotype was present in all of the affected family members

**for complex alleles, higher CADD Phred and SusPect scores of the two variants were taken

*** SuSpect does not score null alleles, therefore in patients with one null allele the combines score is not avaialbe

IRD# The allelic combination was detected in an IRD with some clinical features of Joubert Syndrome

Supplementary Table 5: Primers for variant validation and phasing

INPP5E _c.473dup_c.746C>T_Forward	GCCAGGAGGACCTGGAAG	Used for TA-cloning
INPP5E _c.473dup_c.746C>T_Reverse	GCCTGAACACTACAACGAAGG	Used for TA-cloning
INPP5E _c.914C>T_Forward	GTTAGCAGTGGGGTGCACC	
INPP5E _c.914C>T_Reverse	GGATGTGTCCTGTGCCTGG	
INPP5E _c.1094C>T_Forward	GTTTCCCTGGTAGCTCCTCG	
INPP5E _c.1094C>T_Reverse	CTCAGCTCACCTGTGGGAC	
INPP5E _c.1453C>T_Forward	ATCTCCCGGATGAGCTGGT	
INPP5E _c.1453C>T_Reverse	CAGGTGCCTTCTGGAACTGT	
INPP5E _c.1670G>A / c.1754G>A / c.1800C>G_Forward	GGCGCTTGAGAACAGCAG	
INPP5E _c.1670G>A / c.1754G>A / c.1800C>G_Reverse	CCTCTTAGCTCATGGGACGAC	
INPP5E _c.1862G>A_Forward	CTGCCTCTGAATCTCCTTCG	
INPP5E _c.1862G>A_Reverse	CCACCTAACAGCCCCTCAT	
